# *Atractylodes macrocephala* polysaccharide orchestrates anti-tumor immunity via a dual-network mechanism targeting the gut microbiota and spleen

**DOI:** 10.1038/s41522-026-01013-8

**Published:** 2026-06-26

**Authors:** Yajing Shuai, Jieyu Xing, Xiaoxian Liu, Zijing Song, Siqi Lin, Chengyu Lu, Weiwei Zeng, Guixiang Wang

**Affiliations:** 1 School of Pharmacy, Guangdong Pharmaceutical University, Guangzhou, Guangdong China; 2 Department of Gynecology, Shenzhen Hospital of Southern Medical University, Shenzhen, Guangdong China; 3 Department of Pharmacy, Shenzhen Longgang Second People’s Hospital, Shenzhen, Guangdong China

**Keywords:** Cancer, Computational biology and bioinformatics, Immunology

## Abstract

Colorectal cancer (CRC) is a common global malignancy, and its advanced stage is closely linked to a gut microbiota-metabolism-immunity vicious cycle requiring early intervention. In this study, anti-CRC effects of PAMK in CT26 tumor-bearing mice were explored. Results showed that PAMK significantly inhibited tumor growth and improved the quality of life of tumor-bearing mice by enhancing antitumor immunity, including increased NK cell infiltration and NKG2D expression, elevated CD4⁺:CD8⁺ ratios, and higher serum IFN-γ levels. However, therapeutic effects of PAMK were not observed in antibiotic‑induced microbiota depletion (AIMD) mice. Notably, following fecal microbiota transplantation (FMT), therapeutic effects of PAMK were largely restored. PAMK alleviated tumor-induced gut microbiota dysbiosis characterized by enriched *g_Alistipes*, and remodeled fatty acid and steroid metabolism, which was closely associated with enhanced antitumor immunity and a potential microbiota–metabolism–immunity axis. Meanwhile, PAMK modulated multiple metabolic, circadian and immune pathways in the spleen as verified by transcriptomics and qPCR. Integrative multi-omics analysis indicated that the gut microbiota–metabolite–spleen gene axis may act synergistically to mediate anti-CRC effects of PAMK in tumor-bearing mice. This study highlights the potential of PAMK in CRC tumor immune adjuvants, providing experimental and theoretical support for its clinical translation and novel tumor immunotherapies.

## Introduction

Colorectal cancer (CRC) ranks as the third most common malignant neoplasm worldwide and the second leading cause of cancer-related mortality globally, with its high incidence and mortality posing a major global public health challenge^1^. More critically, over 80% of patients are diagnosed at the advanced stage of the disease, missing the optimal window for radical treatment^2^. The treatment of advanced CRC faces multiple bottlenecks: on one hand, tumor progression significantly reduces the rate of surgical resection; although radiotherapy and chemotherapy can prolong patients’ survival to a certain extent, they tend to induce severe side effects such as immunosuppression and gut microbiota dysbiosis, which in turn impair patients’ quality of life^3^. On the other hand, the vicious cycle of “gut microbiota dysbiosis-metabolic disorder-immunosuppression” within the tumor microenvironment further accelerates tumor progression and therapeutic resistance, which constitutes a core obstacle limiting therapeutic efficacy^4,5^. Therefore, exploring early intervention strategies and novel therapeutic strategies with low toxicity and high efficacy that target and break this cycle has become an urgent need in the current field of CRC research.

In the field of adjuvant CRC therapy, natural polysaccharides have emerged as a research hotspot in recent years due to their unique advantages of low toxicity, multi-target regulation, and immunomodulation. Existing clinical practice and basic research have confirmed: Lentinan can enhance the body’s anti-tumor immune response by activating the functions of NK cells and T cells, while alleviating gastrointestinal toxicity induced by oxaliplatin-based chemotherapy^6^; Ginseng Polysaccharide Injection can significantly reduce the proportion of Treg cells, effectively mitigating the immunosuppressive state of advanced CRC patients after radiotherapy and chemotherapy^7^; Coriolus versicolor polysaccharide can regulate the Toll-like receptor 4/nuclear factor-κB (TLR4/NF-κB) signaling pathway to enhance the activity of macrophages, thereby strengthening immune killing capacity ^8^. Additionally, the candidate drug “BG136 for Injection” (a polysaccharide derived from fungi) has entered the phase Ⅱ clinical trial stage for advanced solid tumors, and preliminary data have shown a certain objective response rate in CRC, further confirming the therapeutic potential of polysaccharide-based substances^9^. However, most current studies on polysaccharides are still limited to exploring the regulation of a single immune pathway (e.g., TLR4 or NF-κB pathway), with relatively insufficient attention paid to the synergistic modulation of the “gut microbiota-metabolism-immunity” integrated axis^10,11^. In particular, systematic analyses are lacking on the regulatory mechanisms underlying the “metabolism-circadian rhythm-immunity” homeostasis in core immune organs such as the spleen, which greatly restricts the comprehensive understanding of the mechanism of action of polysaccharide-based substances.

*Atractylodes macrocephala Koidz*., a plant belonging to the genus *Atractylodes* in the Asteraceae family, has its dried rhizome as an important component of traditional Chinese medicine, exhibiting various pharmacological activities including anti-tumor, anti-inflammatory, gastric mucosa protection, and anti-depressant effects^12^. Its main bioactive constituents include polysaccharides, polyacetylenes, and sesquiterpenoids. Among them, polysaccharide of *Atractylodes macrocephala Koidz* (PAMK) has become a research focus in recent years due to its immunomodulatory and gut microbiota-regulating activities. Existing studies have shown that PAMK can effectively increase the spleen index in mice, improve the morphological structure of splenic cells, and reduce abnormal cell death^13^, while promoting the secretion of key cytokines such as IFN-γ, IL-1α, IL-21, and IFN-α^14^. Meanwhile, PAMK can regulate the balance between helper T cells and cytotoxic T cells, alleviate lipopolysaccharide (LPS)-induced inflammatory responses and oxidative stress damage in mice^14^, and achieve beneficial remodeling of the gut microecology by increasing gut microbial diversity and inhibiting the proliferation of harmful bacteria^15^. Although PAMK has demonstrated potential in regulating gut microbiota and improving immunosuppressive states in mice in previous studies^16^, its specific anti-tumor effect on CRC, whether it exerts its function through gut microbiota mediation, and how it reverses the tumor-induced pathological state by regulating the “metabolism-rhythm-immunity” network have not yet been systematically reported. This research gap seriously hinders the clinical transformation and application of PAMK.

In this study, mice bearing CT26 CRC subcutaneous syngrafts—a model widely used in the research of CRC immunoregulation mechanisms^17^—were employed, and the anti-CRC effect and potential mechanism of PAMK were systematically investigated by integrating transcriptomics (RNA-seq), untargeted metabolomics, 16S rRNA gut microbiota sequencing, and molecular docking technology, with the goal of providing detailed and reliable animal experimental data support for the clinical transformation of PAMK as an immune adjuvant for CRC.

## Results

### PAMK suppressed tumor growth in CT26 tumor-bearing mice

To clarify the intervention value of PAMK in CT26 tumors, the therapeutic efficacy of two different administration timings on tumor growth: one received treatment starting at the time of tumor inoculation (PAMK groups), and the other received treatment from 14 days prior to tumor inoculation (Pre-PAMK groups), were evaluated before this study. The results revealed that two interventions of PAMK exhibited certain anti-CRC effects, accompanied by an enhancement of the immune response. However, the anti-tumor efficacy of PAMK pre-intervention (Pre-PAMK groups) was more superior to concurrent intervention (PAMK groups, Supplementary Fig. 1).

**Fig. 1:**
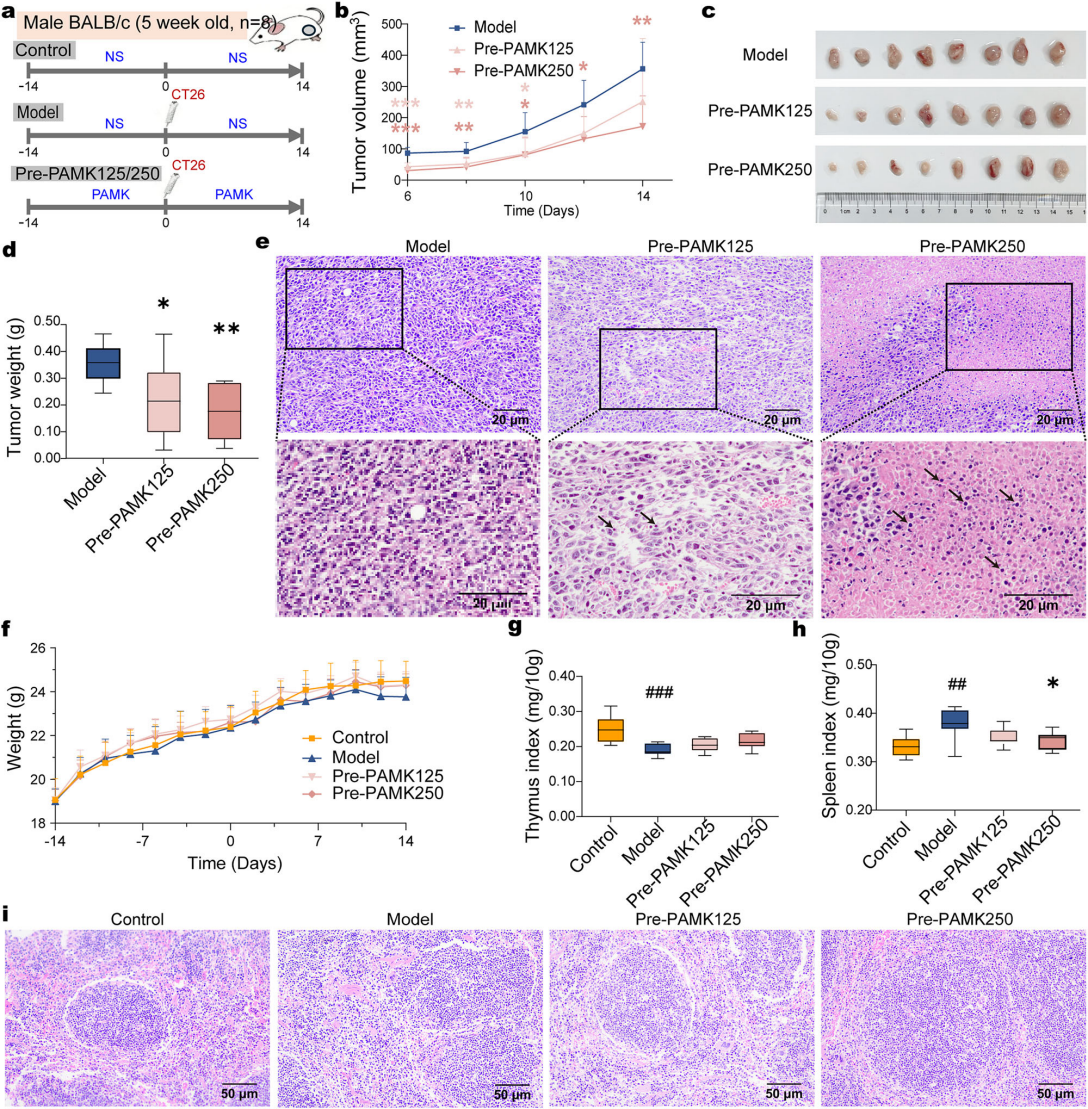
PAMK suppressed colorectal cancer growth in CT26 tumor-bearing mice (*n* = 8). **a** Experimental process overview; **b** Mice tumor volume changes; **c** Image of mice-derived tumor tissues; **d** Weight of tumor tissues; **e** Mice tumor microstructure (scale bar = 20 μm); **f** Mice body weight changes; **g** Thymus indices of mice; **h** Spleen indices of mice; **i** Mice splenic microstructure (scale bar = 50 μm). Statistical significance was indicated as: ^#^/*, *P* < 0.05; ^##^/**, *P* < 0.01; ^###^/***, *P* < 0.001. ^#^ vs. Control group; * vs. Model group.

To fully assess the anti-CRC effect of PAMK pre-intervention and classify its mechanism, the doses of 125 mg/kg and 250 mg/kg were selected (Fig. 1a) based on our preliminary experiments, which demonstrated favorable anti-tumor efficacy, mild effects on body weight, and improved immune organ indices compared with the 500 mg/kg dose (Supplementary Fig. 1). First, regarding tumor growth of mice in Pre-PAMK groups, compared with the Model group (characterized by uncontrolled tumor proliferation), the tumor growth rate was significantly inhibited (Fig. 1b), and the final tumor volume and tumor weight were obviously reduced (Fig. 1c, d, all *P* < 0.05). A distinct dose-dependent trend was observed: substantially stronger anti-tumor activity was exhibited in the Pre-PAMK250 group than in the Pre-PAMK125 group. Specifically, the tumor weight of the Pre-PAMK250 group decreased by 6.95% compared with the Model group, and its average tumor volume (183.06 mm3) was significantly smaller than that of the Model group (356.25 mm^3^, *P* < 0.01). Although tumor growth was also suppressed in the Pre-PAMK125 group, its inhibitory effect was less prominent than that of the Pre-PAMK250 group. HE staining results of tumors also revealed that compared with the Model group, tumor tissues in the Pre-PAMK groups exhibited reduced tumor cell density, widened stroma, irregular morphology, extensive vacuolation and expanded necrotic areas (indicated by black box, Fig. 1e). Concurrently, compared with the Model group, the gradual recovery of body weight was observed in mice from the Pre-PAMK groups. No significant difference in weigh status was found between the Pre-PAMK250 group and the Control group (*P* > 0.05, Fig. 1f); compared with the Control group, a significant decreased thymus index was observed in the Model group (*P* < 0.001), while the Pre-PAMK groups showed respective increase of 7.19% and 8.91% relative to the Model group, neither of which was statistically significant (*P* > 0.05; Fig. 1g); the Model group exhibited a 23.76% increase in the spleen index compared with the Control group (*P* < 0.01), whereas the Pre-PAMK groups showed reduced spleen indices relative to the Model group—with a significant difference observed in the Pre-PAMK250 group (*P* < 0.05; Fig. 1h); HE staining results of spleen pathological sections indicated disrupted structure and increased necro and increased necrotic foci in the Model group (Fig. 1i), suggesting tumor-induced immune tolerance in mice. In contrast, the structural damage of the spleen was alleviated in the Pre-PAMK groups, with the Pre-PAMK250 group showing a more obvious recovery trend (Fig. 1i).

Collectively, these results confirmed that PAMK pretreatment can not only effectively suppressed CRC tumor growth but also mitigated tumor-induced systemic damage, and this effect may be closely related to immune regulation mechanisms.

### PAMK pretreatment enhanced immune function in CT26 tumor-bearing mice

To verify the immune regulatory effects of PAMK pretreatment, analyses of immune cells and immune effector molecules were performed in mouse spleen and tumor tissues.

**Fig. 2:**
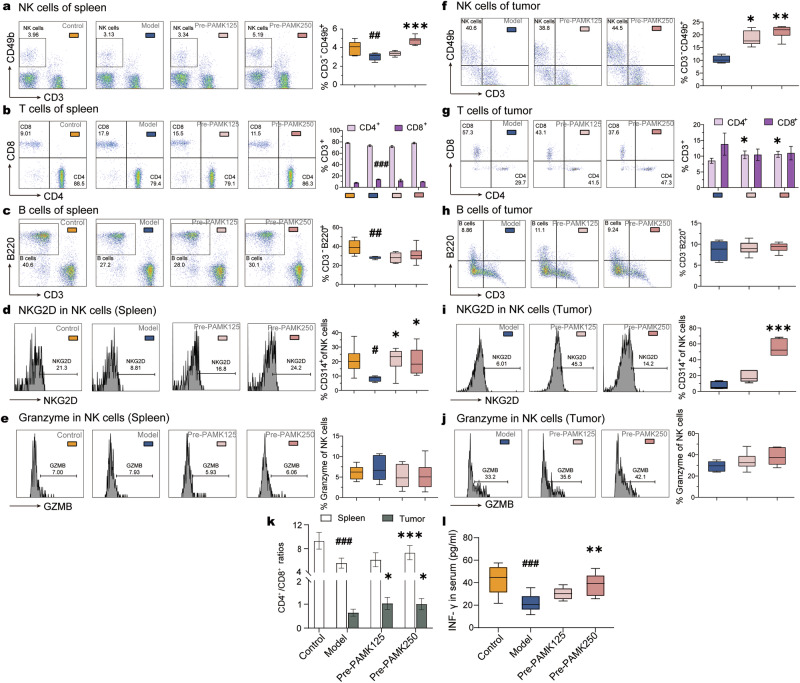
PAMK pretreatment modulated immune function in CT26 tumor-bearing mice (*n* = 6). **a** NK cells in spleen; **b** CD4⁺ T & CD8⁺ T cells in spleen; **c** B cells in spleen; **d** NKG2D expression on NK cells from spleen; **e** Granzyme B expression in NK cells from spleen; **f** NK cells in tumor; **g** CD4⁺ T & CD8⁺ T cells in tumor; **h** B cells in tumor; **i** NKG2D expression on NK cells from tumor; **j** Granzyme B expression in NK cells from tumor; **k** CD4⁺/CD8⁺ T cells ratio in spleen and tumor; **l** IFN-γ levels in serum. Statistical significance was indicated as: ^#^/*/^&^, *P* < 0.05; ^##^/**/^& &^, *P* ^<^ 0.01^; ###^/***/^& & &^, *P* < 0.001. ^#^ vs. Control group; * vs. Model group.

Immune cell subset profiling of mouse spleen tissues revealed that the distribution of immune cell subsets in the Model group differed significantly from that in the Control group: NK cells were significantly decreased (*P* < 0.01, Fig. 2a); CD4⁺ T cells (key immune helper and regulatory cells) exhibited a decreasing trend, albeit without reaching statistical significance (*P* > 0.05), CD8⁺ T cells (core effector cells that directly kill tumor cells) were significantly increased (*P* < 0.001, Fig. 2b); leading to a significantly decreased CD4⁺/CD8⁺ T cell ratio (*P* < 0.001, Fig. 2k); also B cells (core cells of humoral immunity responsible for antibody production) were significantly downregulated (*P* < 0.01, Fig. 2c). However, compared with the Model group, improvements were observed in the Pre-PAMK groups: NK cells were upregulated with a 54.63% increase in the Pre-PAMK 250 group (*P* < 0.001, Fig. 2a); CD4⁺ T cells was upregulated with a 4.24% increase in the Pre-PAMK 250 group, while CD8⁺ T cells were downregulated, with a 19.72% decrease in the Pre-PAMK 250 group with neither change reaching statistical significance (*P* > 0.05, Fig. 2b), while the CD4⁺/CD8⁺ T cell ratio was upregulated with a 54.63% increase in the Pre-PAMK 250 group (*P* < 0.001, Fig. 2k); the proportion of B cells exhibited a non-significant upregulation trend (*P* > 0.05, Fig. 2c). Furthermore, NKG2D (a key activating receptor) on NK cells were significantly downregulated in the Model group relative to the Control group, whereas Pre-PAMK125 and Pre-PAMK250 groups exhibited marked upregulation by 158.46% and 152.96%, respectively, compared with the Model group (all *P* < 0.05, Fig. 2d); Granzyme B of NK cells was slightly upregulated in the Model group compared with the Control group, while it was slightly downregulated in the Pre-PAMK groups compared with the Model group, neither comparison yielded statistically significant differences (*P* > 0.05, Fig. 2e).

Immune cell subset profiling of tumor tissues revealed that, compared with the Model group, NK cells were significantly increased in Pre-PAMK groups (*P* < 0.05, Fig. 2f); CD4⁺ T cells were significantly upregulated (*P* < 0.05, Fig. 2g), CD8⁺ T cells displayed a decreasing trend that did not reach statistical significance (*P* > 0.05, Fig. 2g)—thereby the CD4⁺/CD8⁺T cell ratio was significantly increased (*P* < 0.05, Fig. 2k); No significant difference in the proportion of B cells was observed (*P* > 0.05, Fig. 2h). However, the upregulating effect of PAMK pre-intervention on NKG2D in tumor tissue was more prominent, with an increase of 655.85% in the Pre-PAMK250 group (*P* < 0.001, Fig. 2i); A slight upregulation of Granzyme B was observed in the Pre-PAMK groups without reaching statistical significance (*P* > 0.05, Fig. 2j). IFN-γ (interferon-γ), a core antitumor cytokine secreted by various immune cells, was significantly downregulated in the Model group relative to the Control group (*P* < 0.001); while compared with the Model group, the Pre-PAMK groups exhibited marked upregulation with the Pre-PAMK125 group showing a 8.36% elevation and the Pre-PAMK250 group a 16.61% elevation (*P* < 0.01; Fig. 2l).

These findings indicated that CT26 tumors induced antitumor immune microenvironment dysregulation by suppressing innate immunity (reduced NK cells) and triggering adaptive immune dysfunction (ineffective CD8⁺ T cell proliferation, imbalanced T cell subsets), thereby fostering an immunosuppressive microenvironment that facilitated tumor progression. Consistently, PAMK pretreatment mitigated splenic immune dysregulation, enhanced tumor immune cell infiltration, effectively activated NK cells and specifically upregulated the expression of their activating receptor NKG2D in tumor tissues and enhanced the body’s antitumor immune response by upregulating the level of the core antitumor cytokine IFN-γ in serum. Collectively, while significantly inhibiting the growth of CT26 tumors and alleviating tumor-induced weight gain retardation in mice, PAMK also substantially mitigated tumor-induced functional impairment of immune organs (thymus and spleen), thereby enhancing the antitumor immune response.

### PAMK pretreatment failed to suppress tumor growth in CT26 tumor-bearing AIMD mice

**Fig. 3:**
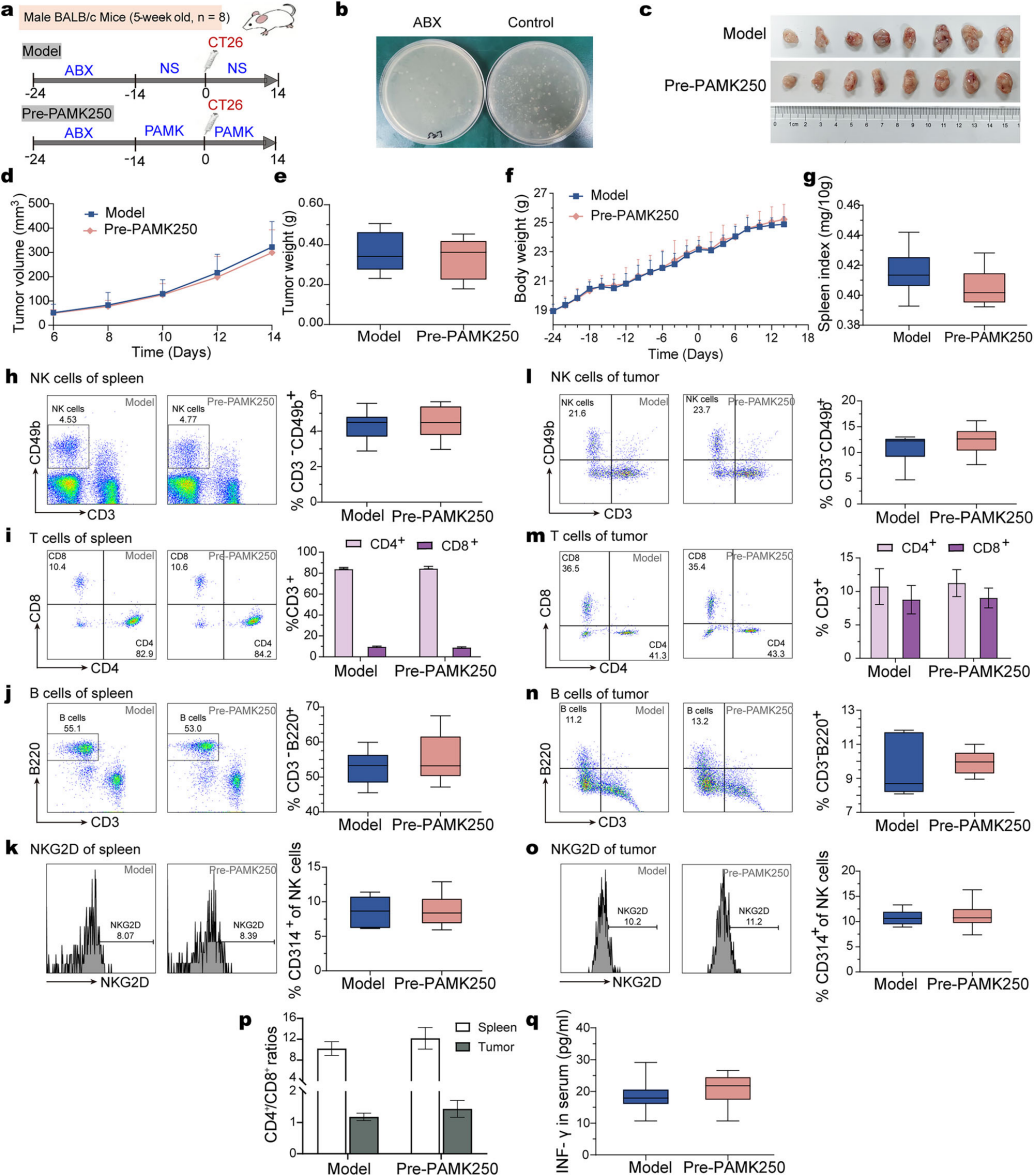
PAMK pretreatment failed to suppress tumor growth in antibiotic-induced microbiota depleted (AIMD) mice. **a** Experimental process overview; **b** Spread plate validation of gut microbiota depletion in AIMD mice (Representative images); **c** Image of mice-derived tumor tissues; **d** Mice tumor volume changes; **e** Weight of mice-derived tissues; **f** Mice body weight changes; **g** Spleen indices; **h–k** Immune cells (NK, CD4⁺ T, CD8⁺ T & B cells, NKG2D expression) in spleen (*n* = 6); **l–o** Immune cells (NK, CD4⁺ T, CD8⁺ T & B cells, NKG2D expression) in tumor (*n* = 6); **p** CD4⁺/CD8⁺ T cells ratio in the spleen and tumor tissues; **q** IFN-γ levels in serum.

PAMK has been proved to possess gut microbiota-regulating potential. To clarify the core role of the gut microbiota in its antitumor mechanism, an AIMD model was constructed and employed to verify whether PAMK’s antitumor efficacy is mediated by the gut microbiota (Fig. 3a).

After 10 consecutive days of antibiotic intervention, AIMD model mice exhibited a typical microbiota depletion phenotype: lose body weight, rough and yellowish fur, and diarrhea in some individuals. Bacterial culture results further confirmed the successful model establishment—compared with healthy mice, the area and number of bacterial colonies in the feces of antibiotic-treated mice showed a significant decreasing trend after 24-h culture (Fig. 3b). On this basis, compared with the Model group, the Pre-PAMK250 group had no statistically significant differences in core indicators of its antitumor efficacy including tumor growth kinetics and gross tumor morphology (Fig. 3c–e), body weight growth rate (Fig. 3f), spleen index (Fig. 3g), composition of immune cells subsets and expression level of the activating receptor NKG2D on NK cells in the spleen (Fig. 3h-k) and tumor immune microenvironment (Fig. 3l–o), as well as the ratio of CD4^+^/CD8^+^ T cells (Fig. 3p) and secretion level of the key cytokine IFN-γ (Fig. 3q).

These findings indicated that PAMK pretreatment failed to suppress tumor growth and also failed to enhance immune function in AIMD mice, suggesting that the gut microbiota may be required for the antitumor and immunoregulatory effects of PAMK.

### PAMK pretreatment exerted anti-tumor efficacy in CT26-tumor bearing AIMD mice via fecal microbiota transplantation

**Fig. 4:**
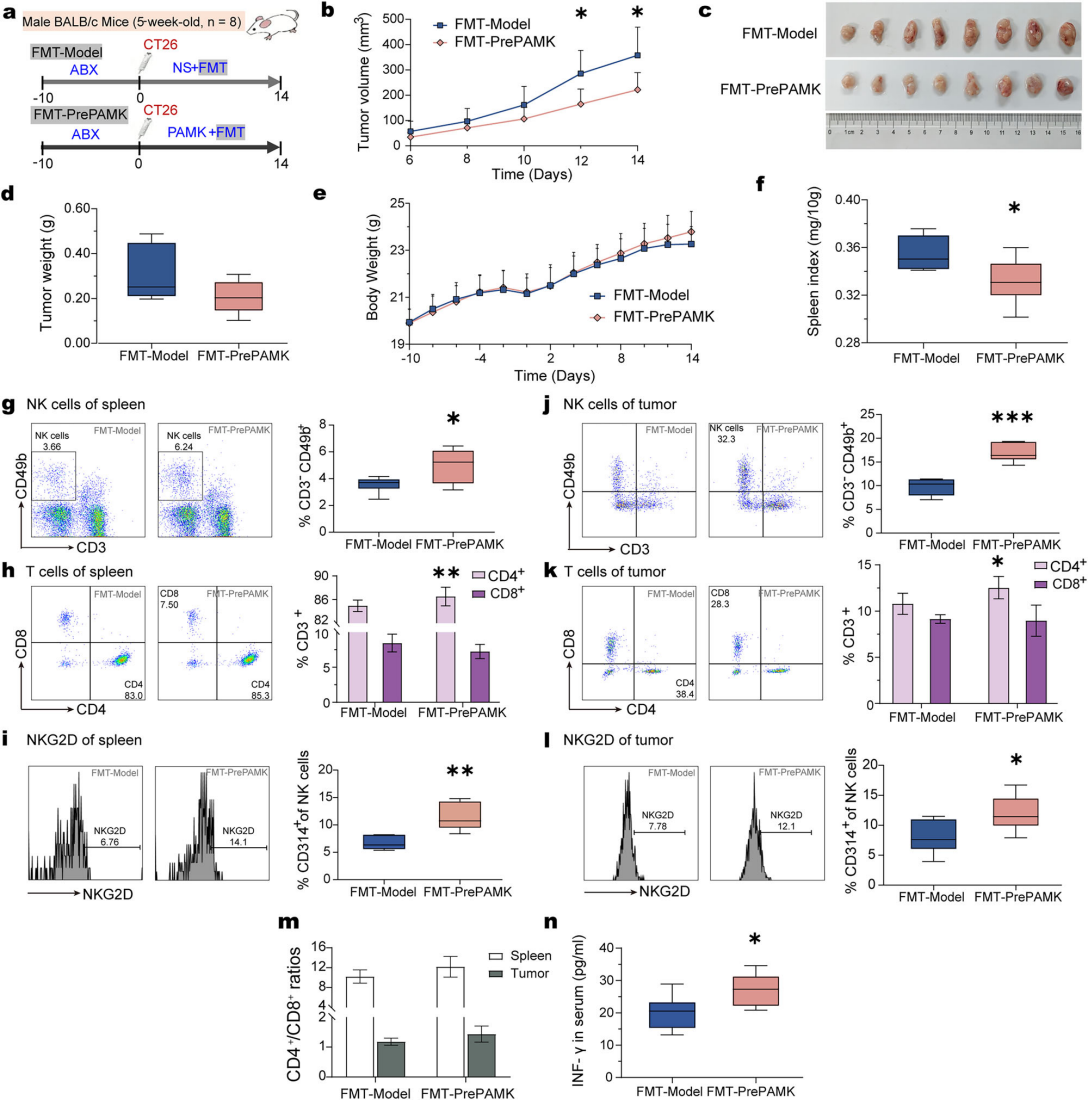
PAMK pretreatment exerted anti-tumor efficacy in AIMD CT26-tumor bearing mice via fecal microbiota transplantation. **a** Experimental process overview; **b** Mice tumor volume changes; **c** Image of mice-derived tumor tissues; **d** Weight of mice-derived tissues; **e** Mice body weight changes; **f** Spleen indices of mice; **g–i** Immune cells (NK, CD4⁺ T, CD8⁺ T cells, NKG2D expression) in spleen (*n* = 6); **j–l** Immune cells (NK, CD4⁺ T, CD8⁺ T cells, NKG2D expression) in tumor (*n* = 6); **m** CD4⁺/CD8⁺ T cell ratios in spleen and tumor tissues; **n** IFN-γ levels in serum (*n* = 8). Statistical significance was indicated as: *P < 0.05, **P < 0.01, ***P < 0.001 vs. Model group.

To confirm the dependence of PAMK’s antitumor efficacy on the gut microbiota, this study specifically designed a FMT experiment (Fig. 4a), aiming to verify whether restoring a specific microbial community can effectively restore the antitumor activity of PAMK. Results showed that after receiving FMT intervention, the general physiological status of mice were significantly improved, and significant differences were observed between the two groups: the Pre-PAMK250 group reexhibited a prominent antitumor effect—the tumor growth rate was significantly slowed (*P* < 0.05; Fig. 4b), and both the tumor volumes and weights demonstrated a decreasing trend, though these reductions did not achieve statistical significance (*P* > 0.05; Fig. 4c, d); Meanwhile, the Pre-PAMK250 group effectively reversed the declining trend of tumor induced mouse body weight (Fig. 4e), and the tumor induced increased spleen index (*P* < 0.05; Fig. 4f). Immune status in spleen was changed: the Pre-PAMK250 treatment group showed a significant increase of NK cells by 1.40% (*P* < 0.05), a higher ratio of CD4⁺/CD8⁺ T cells (Fig. 4g) with the proportion of CD4⁺ T cells increased by 1.57% (*P* < 0.01) and CD8⁺ T cells decreased by 1.20% (Fig. 4h); the Pre-PAMK250 treatment group also showed a significant increase of the NKG2D expression (*P* < 0.01, Fig. 4i); immune status in tumor tissue was also changed: the Pre-PAMK250 treatment group showed a significant increase in NK cells (*P* < 0.001, Fig. 4j), a higher ratio of CD4⁺/CD8⁺ T cells (Fig. 4m) with an significant increase in CD4⁺ T cells by 16.16% (*P* < 0.05) and a non-significant decreasing trend in CD8⁺ T cells by 1.98% (*P* > 0.05; Fig. 4k); the Pre-PAMK250 group also showed a significant increase of the NKG2D expression (*P* < 0.05, Fig. 4l). In addition, serological analysis further revealed a significant increase in IFN-γ levels in the Pre-PAMK250 group compared with the Model group (*P* < 0.05; Fig. 4n).

These findings demonstrated that FMT treatment reinstated PAMK’s antitumor efficacy in a manner consistent with observations in normal mice, supporting the critical role of gut microbiota in host immune function and PAMK-mediated antitumor activity. Specifically, gut microbiota-enhanced immune responses were associated with alleviated tumor-induced immunosuppression, particularly that of NK cells and CD4⁺ T cells in the tumor microenvironment, thereby contributing to enhanced antitumor capacity in mice. These results support that gut microbiota may serve as a key mediator of PAMK’s antitumor effects.

### PAMK pretreatment remodulated aberrant gut microbiota structure in CT26 tumor‑bearing mice

To evaluate the impact of PAMK pretreatment on the gut microbiota in CT26 tumor-bearing mice, mouse fecal samples (groups of Control, Model and Pre-PAMK250) were collected and microbial community analysis was performed using 16S rRNA sequencing technology.

**Fig. 5:**
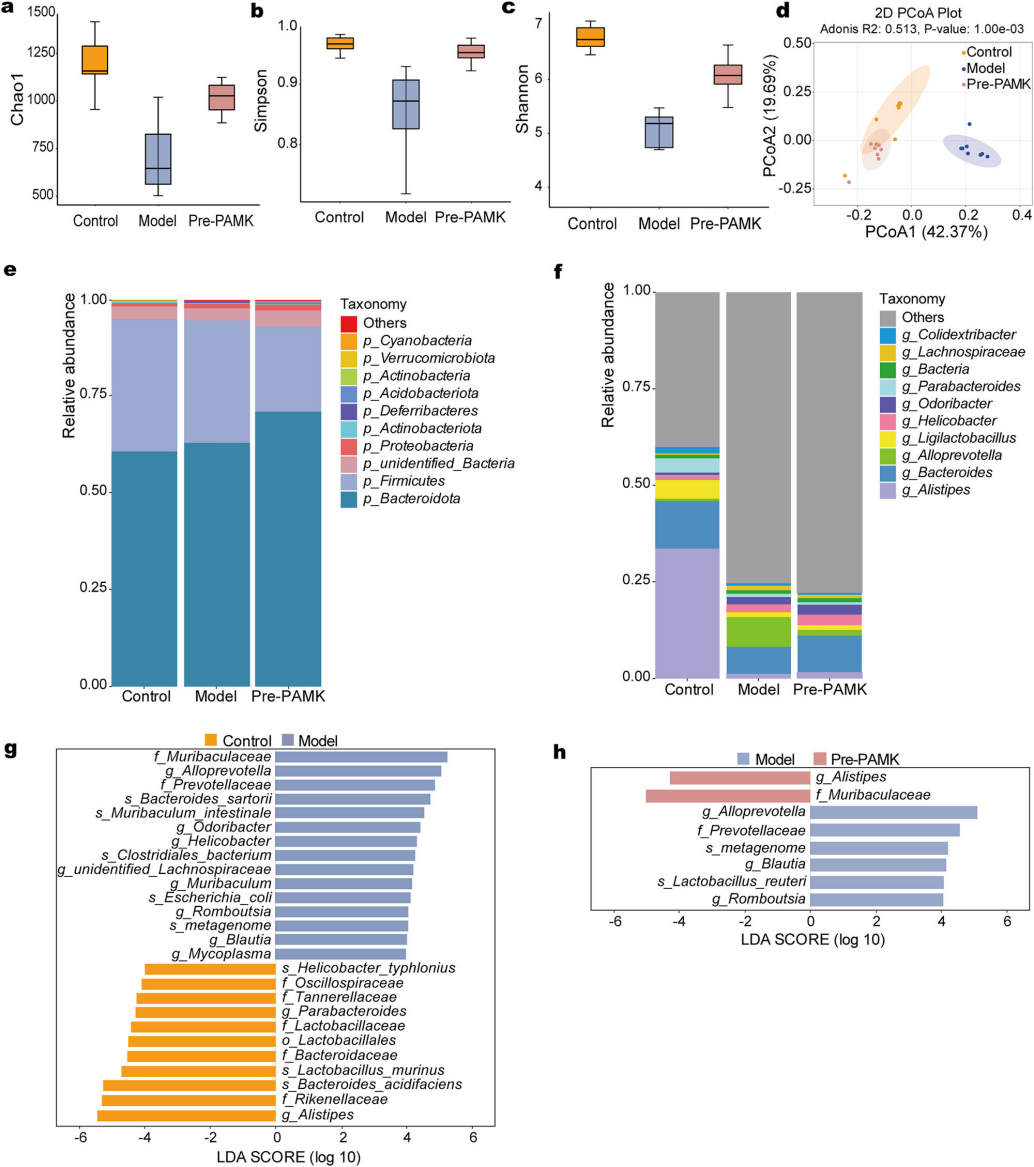
PAMK pretreatment remodulated aberrant gut microbiota structure in CT26 tumor-bearing mice (*n* = 8). **a–c** Gut microbiota α-diversity indices (Chao1, Shannon, Simpson). **d** PCoA analysis of gut microbiota β-diversity. **e**, **f** Taxonomic abundance profiles at the phylum/genus levels. **g**, **h** LEfSe analysis of differentially abundant taxa (LDA score > 3.8).

For microbial diversity, the Control group exhibited high indices of Chao1, Shannon, and Simpson, while the Model group exhibited significantly lower indices, which were elevated in the Pre-PAMK250 group (Fig. 5a–c). Moreover, the gut microbiota of the Model group was scattered and distant from the Control group cluster, while that of the Pre-PAMK250 group and the Control group clustered closely (Fig. 5d). Regarding the structure of microbial community, at the phylum level, although the main microbial phyla in all groups were *Bacteroidota* and *Firmicutes*, followed by *undefined Bacteria*, *Proteobacteria*, and *Actinobacteria*, compared with the Control group, the Model group exhibited significantly increased abundances of *Deferribacteres* (3.21-fold), *Verrucomicrobiota* (6.39-fold), *Cyanobacteria* (10.35-fold), and significantly decreased abundances of *Actinobacteriota* (0.42-fold), *Actinobacteria* (0.60-fold), and *Acidobacteriota* (0.01-fold); Compared with the Model group, the Pre-PAMK250 group showed significantly decreased *Deferribacteres* (0.33-fold), *Verrucomicrobiota* (0.23-fold) and significantly increased *Actinobacteria* (2.39-fold) and *Acidobacteriota* (190.43-fold) (Fig. 5e). At the genus level, the Control group exhibited a high ratio of *Alistipes* and *Bacteroides* and *Ligilactobacillus* and a small portion of *Alloprevotella* and unidentified_*Lachnospiraceae*; compared with the Control group, the Model group exhibited exhibited significantly increased abundances of *unidentified_Lachnospiraceae* (2.43-fold)*, Alloprevotella* (13.01-fold)*, Odoribacter* (2.57-fold) and significantly decreased abundances of *Colidextribacter* (0.35-fold), *Parabacteroides* (0.21-fold), *Ligilactobacillus* (0.23-fold), *Bacteroides* (0.58-fold), *Alistipes* (0.03-fold), however, the Pre-PAMK250 group was observed to upregulate the first two increased ones and downregulate the later three decreased ones (Fig. 5f), indicating that PAMK modulated gut microbiota structure and attenuated tumor-induced microbial dysbiosis.

LEfSe analysis (screening criteria: LDA [Linear Discriminant Analysis] score > 3.8) was used to identify group-specific differential taxa of gut microbiota. As shown in Fig. 5g, h, the Model group was characterized by specific enrichment of six dysbiosis-related taxa across multiple taxonomic levels: *f_Prevotellaceae* (a classic marker of gut mucosal damage and metabolic imbalance); *g_Alloprevotella*, *g_Blautia*, and *g_Romboutsia* (associated with gut inflammation and barrier dysfunction); *s_Lactobacillus_reuteri*; and metagenome-related taxa (*s_metagenome*, indicating functional microbial imbalance). The Control group was characterized by the enrichment of health-promoting taxa such as *g_Alistipes*, *f_Lactobacillaceae*, *s_Lactobacillus_murinus*, and *f_Oscillospiraceae*, which are known to maintain gut homeostasis through short-chain fatty acid production and anti-inflammatory effects. Meanwhile, the Pre-PAMK250 group was characterized by *g_Alistipes*, a genus-level core health-promoting taxon known to maintain gut homeostasis through short-chain fatty acid synthesis, anti-inflammatory effects, and gut barrier protection.

Collectively, these results indicated that treatment with PAMK was associated with the amelioration of tumor-induced abnormalities in gut microbiota structure, characterized by the specific enrichment of *g_Alistipes*, which may contribute to the maintainance of gut homeostasis.

### PAMK pretreatment affected the metabolites of gut microbiota in CT26 tumor-bearing mice

To clarify the underlying mechanism of PAMK, untargeted metabolomics analysis was performed on fecal samples collected from relevant mice. Intergroup differential metabolites were identified with screening thresholds set as |Log₂FC | > 1, VIP > 1, and *P* < 0.05.

**Fig. 6:**
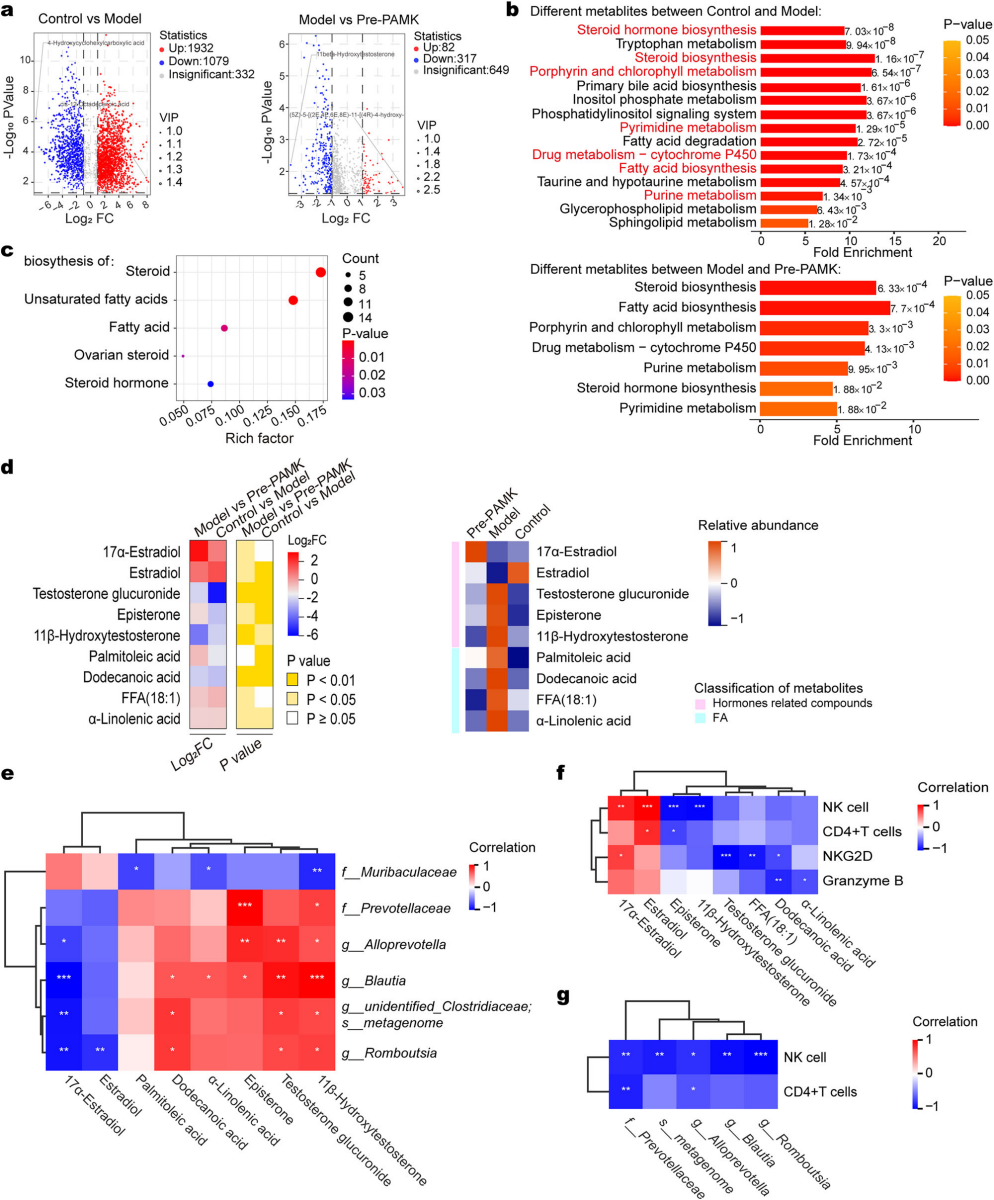
PAMK pretreatment coordinately regulated metabolic, circadian, and immunological processes in CT26 tumor-bearing mice. **a** Volcano plots of differential metabolites (*n* = 8); **b** MSEA enrichment analysis; **c** KEGG enrichment analysis; **d** Cluster analysis; **e** Correlation heatmap of differential metabolites and microbes; **f** Correlation heatmap of differential metabolites and immune markers; **g** Correlation heatmap of differential microbes and immune cells.

It was found that compared with the Model group, the Pre-PAMK250 group had 317 upregulated and 82 downregulated metabolites (Fig. 6a), which largely enriched in 5 pathways including the fatty acid biosynthesis and degradation, steroid and steroid hormone biosynthesis, and porphyrin and chlorophyll metabolism by MSEA analysis (Fig. 6b), of which the first 4 pathways was also confirmed by KEGG analysis (Fig. 6c); compared with the Control group, the Model group had 1,932 upregulated metabolites and 1,079 downregulated metabolites, which were involved in much more pathways including the above 5 pathways; suggesting tumors induced systemic metabolic disorders via microbiota; and PAMK exerted a regulatory effect on them (Fig. 6a–c). It also found that the Model group exhibited the significantly high levels of fatty acid substances (including dodecanoic acid, palmitoleic acid, α-linolenic acid, and free fatty acid [FFA, 18:1]) and androgen-related metabolites (including 11β-hydroxytestosterone, testosterone glucuronide, and epitestosterone) compared with the Control group and Pre-PAMK250 group, but the significantly low levels of estrogen-related substances (including estradiol and 17α-estradiol); in contrast, the Pre-PAMK250 group upregulated the decreased estrogen-related substances, and downregulated the increased fatty acid substances and androgen-related metabolites (Fig. 6d).

Associations between specific gut microbiota and characterized metabolites were also found: for the Pre-PAMK250 group, specifically enriched *f_Muribaculaceae* was positively correlated with the upregulated estradiol and 17α-estradiol; for the Model group, specifically enriched *f_Prevotellaceae*, *g_Alloprevotella*, *g_Romboutsia*, *g_Blautia* and *s__metagenome* were positively correlated with the upregulated palmitoleic acid, testosterone glucuronide, 11β-hydroxytestosterone and epitestosterone, respectively (Fig. 6e). Furthermore, a “microbiota-metabolite-immunity” regulatory network was also constructed: elevated NK cells were positively correlated with upregulated 17α-estradiol and estradiol, and negatively correlated with downregulated 11β-hydroxytestosterone and epitestosterone; elevated CD4⁺ T cells were positively correlated with upregulated estradiol and negatively correlated with downregulated episterone; elevated NKG2D (a key activating receptor of NK cells) was positively correlated with upregulated 17α-estradiol and negatively correlated with downregulated testosterone glucuronide, FFA (18:1), and dodecanoic acid; elevated Granzyme B was negatively correlated with downregulated dodecanoic acid and α-linolenic acid (Fig. 6f). Meanwhile, decreased NK cells and CD4⁺ T cells in the Model group were negatively correlated with the upregulated palmitoleic acid, testosterone glucuronide, 11β-hydroxytestosterone, and epitestosterone, along with enriched *f_Prevotellaceae, g_Alloprevotella, g_Romboutsia*, and *g_Blautia*, respectively (Fig. 6g).

Collectively, these results showed that PAMK was significantly associated with the regulation of gut microbiota (mainly involved in fatty acid and steroid metabolism pathways), and was closely correlated with enhanced immune function (including upregulated NK and CD4⁺ T cells and elevated expression of key immune molecules), thereby contributing to the formation of a potential“microbiota-metabolism-immunity” regulatory network.

### PAMK pretreatment modulated gene expression profiles in spleen in CT26 tumor-bearing mice

As the largest immune organ of the body, the spleen stores a large number of immune cells such as NK cells, T cells, and B cells, and serves as the core support for tumor immune responses. To further clarify the underlying mechanism of PAMK, RNA-seq analysis was performed on mouse spleen tissues to map gene expression profiles, and DEG analysis was conducted using screening thresholds of |Fold Change | ≥ 1.5 and *P* < 0.05.

**Fig. 7:**
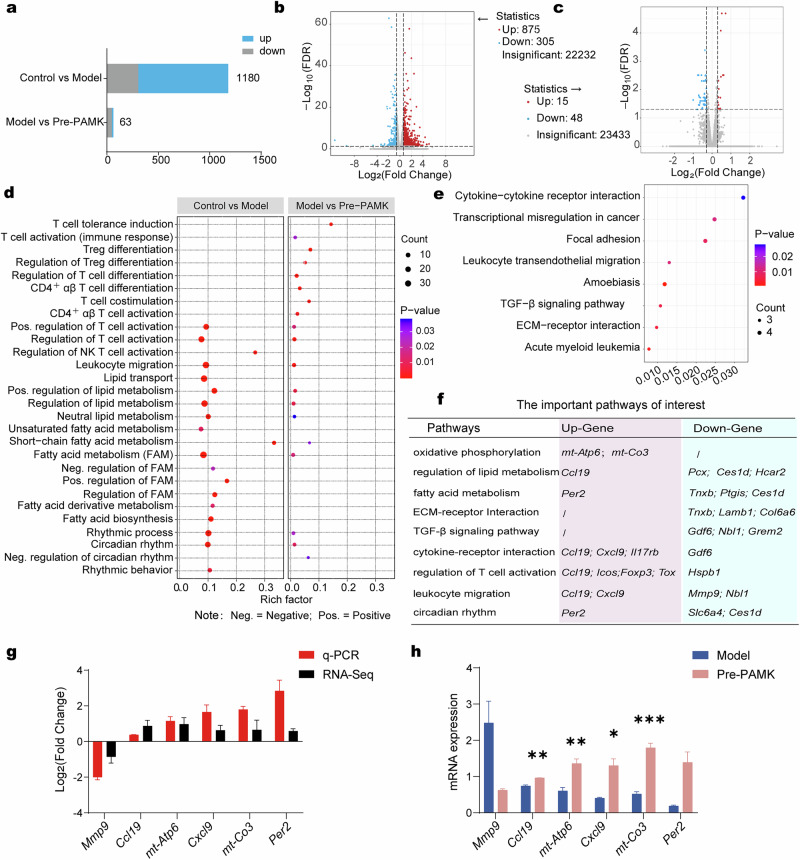
PAMK pretreatment modulated gene expression profiles in spleen of CT26-tumor bearing mice. **a** Statistics of DEGs (*n* = 3); **b** Volcano plot of DEGs between the Control and Model groups. **c** Volcano plot of DEGs between the Model and Pre-PAMK250 groups. **d** KEGG pathway enrichment profile of DEGs: Model vs PrePAMK250; **e** GO functional terms and genes enriched in Model vs. Pre-PAMK250; **f** GO analysis of DEGs between Model and Pre-PAMK250 groups; **g** RNA expression of selected genes in Pre-PAMK250 group (RNA-Seq & RT-qPCR); **h** mRNA expression of selected genes in the Model and Pre-PAMK250 groups (RT-qPCR). Statistical significance was indicated as: *P < 0.05, **P < 0.01, ***P < 0.001 vs. Model group.

It was found that a total of 1180 DEGs were identified in the Model group when compared with the Control group, including 875 upregulated and 305 downregulated genes (Fig. 7a, b), which were enriched in fatty acid metabolism, lipid metabolism-related pathways, and circadian rhythm-related pathways (*P* < 0.05, Fig. 7d); compared with the Model group, 63 DEGs (15 upregulated and 48 downregulated) were identified in the Pre-PAMK250 group (Fig. 7a, c), which were also highly enriched in biological rhythm-related terms (such as circadian rhythm), metabolism-related functional terms (such as regulation of lipid metabolic processes), and multiple T cell-related functional terms (*P* < 0.05, Fig. 7d). The latter two enrichments were significant in both group comparisons, indicating the core role of T cells in immune responses and PAMK can significantly reverse tumor-induced dysregulation of gene expression in the spleen. Moreover, the DEGs in the Pre-PAMK250 group were significantly enriched in key pathways of immune regulation and cellular microenvironment including core immune pathways such as cytokine-cytokine receptor interaction and TGF-β signaling pathway, as well as the ECM-receptor interaction pathway, which co-regulate immune responses, cell growth, and metabolic functions (Fig. 7e, f).

To verify these regulatory effects, RT-qPCR was performed. The expression trends of immune regulation-related target genes [chemokine (C-C motif) ligand 19 (*Ccl19*), chemokine (C-X-C motif) ligand 9 (*Cxcl9*)], mitochondrially encoded ATP synthase 6 (*mt-Atp6*)], and metabolism-related target genes [mitochondrially encoded cytochrome c oxidase III (*mt-Co3*) in the spleen tissues of the Pre-PAMK250 group were consistent with RNA-seq results, showing significant upregulation or downregulation compared with the Model group. However, the expression changes of matrix metallopeptidase 9 (*Mmp9*) and circadian rhythm-related target gene [period circadian regulator 2 (*Per2*)] were not statistically significant (Fig. 7g, h).

Collectively, these findings indicated that PAMK modulated multiple pathways in the spleen, including the fatty acid/lipid metabolism, circadian rhythm, and immune-related pathways such as cytokine-cytokine receptor interaction, and TGF-β signaling. These results, further validated by qPCR, suggested that such regulations may contribute to the tumor growth inhibition observed in CT26 tumor-bearing mice.

### Correlations between differential metabolites and DEGs were validated by the Pearson correlation coefficient, molecular dynamics simulations, and molecular docking

DEGs of spleen tissues revealed that PAMK significantly enhances immunity via regulatory effects on circadian rhythm, fatty acid metabolism, and immune response-related pathways, while gut microbiota metabolites revealed that PAMK significantly regulate core metabolic pathways of gut microbiota including fatty acid biosynthesis, fatty acid degradation, steroid and steroid hormone biosynthesis, and porphyrin and chlorophyll metabolism, suggesting that the antitumor effect of PAMK may depend on a synergistic regulatory network of metabolism-immunity-rhythm. To systematically elucidate the antitumor molecular mechanism of PAMK, multi-dimensional integrated analysis of differential metabolites of gut microbiota (screening criteria: VIP > 1, *P* < 0.05) and DEGs in spleen tissues (screening criteria: |FC | ≥ 1.5, *P* < 0.05) from three mice were performed in this part (*n* = 3).

**Fig. 8:**
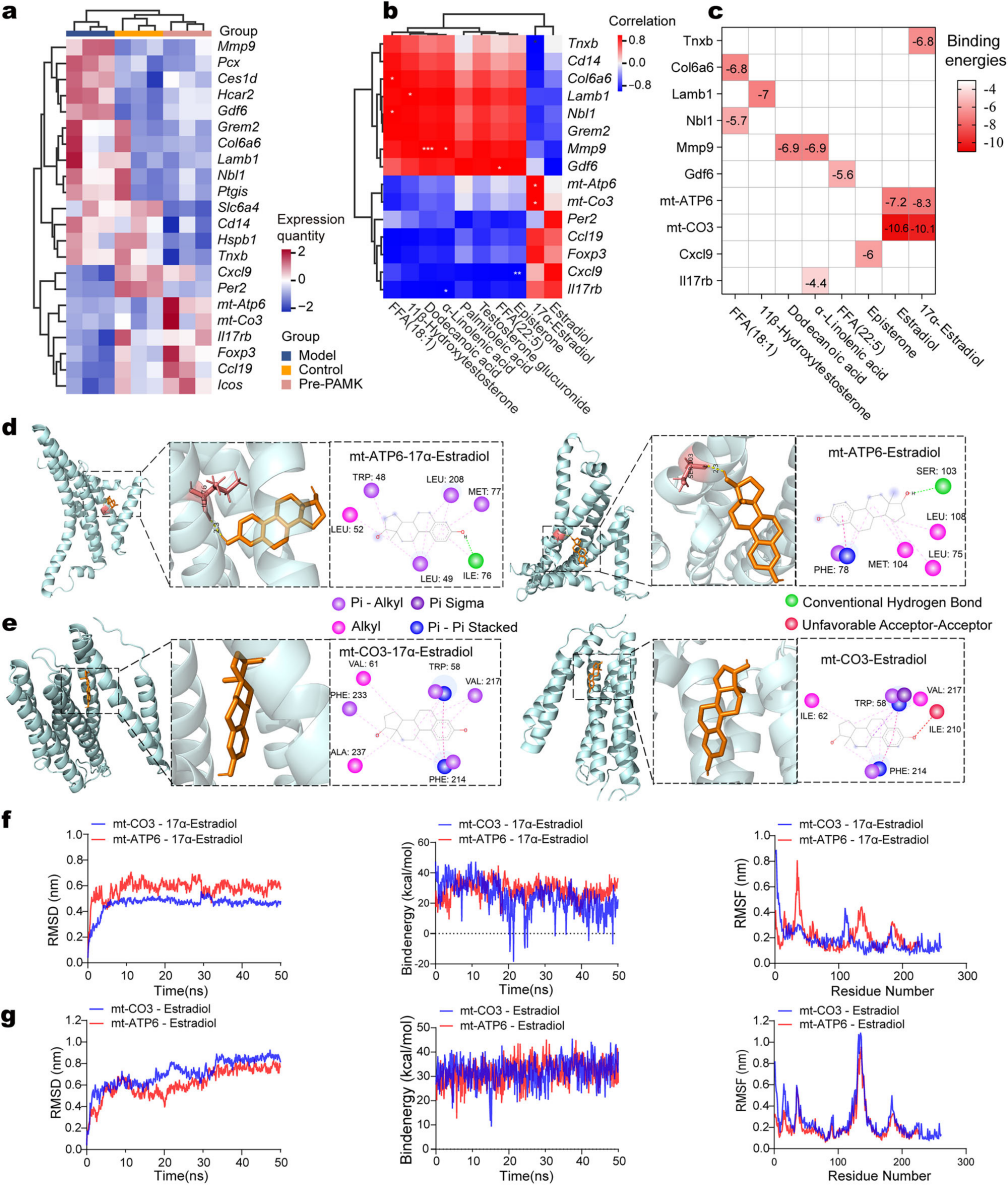
Validation of differential metabolite-DEG correlations via pearson correlation, MD Simulations and Docking. **a** Splenic expression of key genes; **b** Pearson correlation analysis between key genes and metabolites; **c** Binding energies of significantly correlated gene-metabolite pairs; **d**, **e** Molecular docking of representative gene-metabolite pairs using PyMOL; **f**, **g** Molecular dynamics simulations of representative gene-metabolite pairs.

Firstly, the expression characteristics of key genes in differential pathways were analyzed (Fig. 8a): Compared with the Model group, the Pre-PAMK250 group significantly downregulated fatty acid metabolism-related genes such as *Ces1d* and *Ptgis*, effectively correcting tumor-induced dysregulation of the expression of key lipid metabolism-related genes such as *Ccl19*, *Pcx*, and *Hcar2*. Meanwhile, it achieved precise regulation of circadian rhythm-related genes by upregulating the core clock gene *Per2* and downregulating *Slc6a4*, and significantly upregulated T cell function-related genes such as *Foxp3, Cxcl9*, and *Icos*, reversing tumor-induced abnormalities in immune-related gene expression. Additionally, the Pre-PAMK250 group specifically regulated inflammation-related pathway genes: downregulating *Tnxb, Lamb1*, and *Col6a6* in the ECM-receptor interaction pathway, downregulating *Gdf6, Nbl1*, and *Grem2* in the TGF-β signaling pathway, and upregulating *Ccl19, Cxcl9*, and *Il17rb* in the cytokine-cytokine receptor interaction pathway. These regulatory effects may be the core mechanisms underlying the alleviation of splenomegaly and restoration of immune function, ultimately enhancing the host antitumor response. Pearson correlation analysis (Fig. 8b) revealed significant correlations between multiple gene-metabolite pairs, including *Mmp9* and dodecanoic acid (*P* < 0.001), *Cxcl9* and epitestosterone (*P* < 0.01), as well as *mt-Atp6/mt-Co3* and 17α-estradiol (*P* < 0.05).

To verify the interaction between DEGs and differential metabolites, molecular docking experiments were performed on the screened significantly correlated gene-metabolite pairs, and visualization was conducted using PyMOL (Version 2.4.0) and Discovery Studio Visualizer (Version 2019). Results (Fig. 8c–e) showed that 17α-estradiol had strong binding affinity with mt-ATP6 (binding energy: −8.3 kcal/mol) and mt-CO3 (binding energy: −10.1 kcal/mol), while estradiol exhibited binding energies of −7.2 kcal/mol and −10.6 kcal/mol with these two target genes, respectively. The visualization using PYMOL and Discovery Studio Visualizer revealed that two metabolites had hydrogen bonds and other forces for binding with mt-ATP6, while the binding with mt-CO3 lacked hydrogen bonds but had more other forces, such as Pi-Pi stacking and Alkyl. In molecular dynamics simulations, the RMSD values of protein-metabolite complexes tended to stabilize after 35 ns, and the binding free energies were all greater than 0 and relatively stable^18,19^. RMSF analysis (Fig. 8f, g) clarified the flexible and rigid region characteristics of the complexes, confirming the stable binding conformation of the two, which provides computational evidence for the functional interaction between genes and metabolites.

These correlation analyses revealed potential associations supporting that PAMK may exert its antitumor effects via a synergistic regulatory network of “gut microbiota - differential metabolites - spleen genes - antitumor immunity”. Gut microbiota modulated the levels of differential metabolites, which further influenced the expression of immune-related genes in the spleen, and collectively regulated antitumor immune responses, with all components interacting and functioning synergistically. Significant correlations were identified between key metabolites and target genes, and their binding affinity and conformational stability were predicted by molecular docking.

## Discussion

In this study, a mouse model of CT26 subcutaneous syngraft tumors was used as the object to systematically explore the anti-CRC effect and underlying mechanism of *Atractylodes macrocephala* polysaccharides (PAMK).

First, in vivo experiments confirmed the significant anti-tumor value and immune regulatory mechanism of PAMK in CT26 tumor-bearing mice. PAMK could effectively inhibit tumor growth, which was specifically manifested by a significant reduction in tumor volume and weight, as well as the induction of tumor tissue necrosis. Meanwhile, it improved the quality of life of tumor-bearing mice, alleviating tumor-induced slow weight gain and functional disorders of immune organs such as thymus and spleen (Fig. 1). Its core mechanism of action focuses on reshaping the anti-tumor immune response: by increasing the infiltration level and proportion of NK cells and CD4⁺ T cells in tumor tissues and the spleen, specifically enhancing the expression of NKG2D (a key activating receptor on the surface of NK cells), and elevating the concentration of systemic core anti-tumor cytokine IFN-γ, PAMK comprehensively improved the local and systemic immune microenvironment of the body and reversed the tumor-induced immunosuppressive state (Fig. 2). These findings was consistent with the previous study indicating that “tumor-induced immune tolerance/suppression is a key factor in the escape and progression of colorectal cancer”^20^, providing solid support for the immune regulatory effect of PAMK.

Gut microbiota dysbiosis, such as an elevated *Firmicutes* to *Bacteroidetes* (F/B) ratio^21^ and the enrichment of *Prevotellaceae, Alloprevotella*, and *Romboutsia*, and its metabolic abnormalities are closely associated with the formation of immune tolerance in CRC and its progression^22^. In the present study, our results demonstrated that PAMK exerts anti-CRC activity by regulating this core axis and constructing a “microbiota-metabolism-immunity” tripartite regulatory network, as evidenced by the following: ① Verification of microbiota dependence: PAMK failed to inhibit the growth of CT26 tumors in AIMD mice (Fig. 3). However, after FMT, its anti-tumor effect was reactivated (Fig. 4). Moreover, the changes in immune indices in recipient mice were highly consistent with those in donor mice. Such phenotypic transmission following FMT is generally considered to depend on the colonization and functional effects of gut microbiota. These observations support that the immunoregulatory and antitumor effects of PAMK are largely dependent on the gut microbiota; ② Regulation of gut microbiota structure (Fig. 5): 16S rRNA sequencing results showed that the gut microbiota composition of mice intervened with PAMK was closer to that of the healthy Control group. Specifically, it was characterized by a reduced F/B ratio, decreased abundance of potential pathogenic bacteria, and a significant increase in the abundance of the beneficial bacterium *Alistipes*^23^—directly demonstrating PAMK’s role in reshaping the gut microbiota structure; ③ Correction of gut metabolic disorders (Fig. 6): Analyses revealed that PAMK regulated characteristic metabolites predominantly enriched in fatty acid (e.g., dodecanoic acid, palmitoleic acid) and steroid metabolism pathways (e.g., 11β-hydroxytestosterone, estradiol), and effectively reversed the Model group’s metabolic disorders (elevated fatty acids and androgens^24^, reduced estrogens^25^). This correction was not only consistent with the subsequent splenic transcriptomic findings (Fig. 7) but also with those reported in the literatures: PAMK regulates lipid/fatty acid metabolism-related genes such as *Pcx* and *Ces1d*; dodecanoic acid activates TLR4 to promote colitis^26^; FFA (18:1) promotes the development of colorectal tumors^27^; ④ Construction of the “microbiota-metabolism-immunity” network: Integrated analysis clarified the key relationships among the three components. *Alistipes*^23^ enriched in the Pre-PAMK250 group was positively correlated with estradiol, while pathogenic bacteria such as *Prevotellaceae*^28,29^ enriched in the Model group were positively correlated with pro-carcinogenic metabolites such as palmitoleic acid^30,31^. Moreover, estradiol and 17α-estradiol^32,33^ were positively correlated with the levels of NK cells, CD4⁺ T cells, and NKG2D expression, while pro-carcinogenic metabolites including 11β-hydroxytestosterone and dodecanoic acid were negatively correlated with the above immune parameters (Fig. 6f). These observations suggested that PAMK enhanced immune function via the microbiota-metabolism-immunity axis. Further validation using germ‑free mice, targeted bacterial colonization, and metabolite supplementation will be required to establish a definitive causal relationship.

The spleen, as a core immune organ of the body, relies on its functional homeostasis as a key target for evaluating tumor immune responses^34^. Through transcriptomic analysis and validation experiments, this study further revealed the multi-dimensional regulatory effects of PAMK on splenic function (Fig. 7): Tumor burden induced 1180 differentially expressed genes in the spleen, leading to significant disorders in the gene expression profile and thereby enabling tumor immune escape^35^. However, after PAMK intervention, the tumor-induced transcriptomic imbalance was reversed—consistent with the transcriptional remodeling characteristics of effective anti-tumor interventions (Fig. 7b, c). KEGG enrichment analysis identified the core pathway network through which PAMK regulates splenic function: it corrected lipid metabolism disorders by restoring the expression of metabolic genes such as *mt-Co3* and *mt-Atp6*, restored circadian rhythm homeostasis^36^ by regulating core clock genes such as *Per2*^37^, and simultaneously modulated immune-related pathways^28,38^. Specifically, it recruited effector T cells by regulating the *Cxcl9* and *Ccl19* genes in the cytokine-cytokine receptor interaction pathway, and inhibited the activation of regulatory T cells (Treg) by modulating the TGF-β pathway^39^. Combined with the significant enrichment of T cell-related functional terms in GO analysis, these findings revealed that PAMK can specifically enhance T cell-mediated specific immune responses. In addition, RT-qPCR validation confirmed the reliability of the expression trends of most target genes related to immunity, circadian rhythm, and metabolism. Although the downregulation of *Mmp9* was not statistically significant by RT-qPCR, the trend was consistent with RNA-seq results. Based on transcriptomic analysis and previous literature, it is speculated that the downregulation of *Mmp9* may further inhibit tumor invasion, while the regulation of *mt-Co3* and *mt-Atp6* improved mitochondrial energy metabolism in splenic cells^40–42^. Together, these findings suggested the presence of a coordinated regulatory network of “PAMK-metabolism-circadian rhythm-immunity”.

Finally, multi-dimensional integrated analyses were conducted to systematically clarify the molecular details of PAMK’s anti-tumor effect, further verifying the molecular basis of the core regulatory network. The enrichment analysis results of transcriptomics and metabolomics were highly consistent (Fig. 8a, b): in the two comparison groups (Control group vs. Model group, Model group vs. Pre-PAMK250 group), the differentially expressed genes in the spleen and the differential metabolites in the intestine were both significantly enriched in pathways related to circadian rhythm, fatty acid metabolism, and immune response, confirming the core position of the “metabolism-immunity-rhythm” coordinated regulatory network. Analysis of key gene expression characteristics (Fig. 7f) showed that the Pre-PAMK250 group achieved multi-pathway coordination through precise regulation: it downregulated fatty acid metabolism genes such as *Ces1d*^43^ and *Ptgis*^44^, as well as inflammation-related pathway genes such as *Tnxb*^45^ and *Gdf6*^46^; meanwhile, it upregulated rhythm genes such as *Per2* and T cell function-related genes such as *Foxp3*^47^ and *Cxcl9*. This correction of tumor-induced multi-pathway disorders was likely the core molecular mechanism underlying the alleviation of splenomegaly and the recovery of immune function. Pearson correlation analysis (Fig. 8b) revealed significant correlations between multiple gene-metabolite pairs, such as *Mmp9* and dodecanoic acid, *Cxcl9* and epitestosterone, as well as *mt-Atp6/mt-Co3* and 17α-estradiol. Molecular docking and molecular dynamics simulations (Fig. 8c–g) further provided computational evidence: 17α-estradiol and estradiol both exhibited strong binding affinity with mt-ATP6 and mt-CO3 (with the lowest binding energy reaching -10.6 kcal/mol). Moreover, the RMSD values of the protein-metabolite complexes tended to be stable after 35 ns, and the binding free energies were all greater than 0, confirming the stable binding conformation of the two. This provides molecular-level theoretical support for the potential association between genes and metabolites.

Comprehensively, the above research results indicated that PAMK exerted anti-CRC effects through multi-level and multi-pathway coordinated regulation via a dual-network mechanism targeting the gut microbiota and spleen, and its core mechanism could be summarized as “dual-network coordination and multi-target regulation”. On the one hand, PAMK treatment was associated with the attenuation of tumor‑induced the gut microbiota structure (characterized by reduced potentially pathogenic bacteria, enriched beneficial *g-Alistipes*, and modulated the F/B ratio), as well as the alleviation of dysregulated fatty acid and steroid metabolism. These changes were accompanied by enhanced the functions of immune cells such as NK cells and CD4⁺ T cells, supporting a potential peripheral microbiota–metabolism-immunity regulatory axis. On the other hand, PAMK was associated with the mitigation of tumor-induced transcriptomic disorder in the spleen, concurrently modulating metabolic pathways (fatty acid/lipid metabolism), circadian rhythm, which may contribute to the restoration of the immune homeostasis of the spleen, suggesting a potential splenic metabolism-rhythm-immunity axis. Meanwhile, molecular docking analysis supported the potential stable binding between key metabolites such as 17α-estradiol and gene-encoded proteins like mt-ATP6, which provided theoretical support from computational simulations for the cascade regulation of “metabolism-gene-immunity” axis. However, molecular docking and molecular dynamics simulations only provided in silico predictive evidence for interactions between key genes and metabolites. Thus, further experimental validation using biophysical approaches such as isothermal titration calorimetry (ITC) and surface plasmon resonance (SPR), as well as in vivo functional studies, will be necessary to confirm their direct binding and clarify the underlying mechanisms.

This study uncovers PAMK’s potential for CRC prevention and immune adjuvant therapy, offering important experimental and theoretical foundations for its clinical application, and the advancement of spleen-targeted and gut microbiota -oriented tumor immunotherapy.

## Methods

### Chemicals and reagents

All chemicals and reagents were obtained from commercial suppliers and used as received. polysaccharide of *Atractylodes macrocephala Koidz* (PAMK, assay content 90.1%, endotoxin: 0.001907 ng/mg = 0.01907 EU/mg) was sourced from Shaanxi Mufei Biotech Co., Ltd. (China). The following cell culture reagents were acquired from Thermo Fisher Scientific (USA): penicillin-streptomycin, fetal bovine serum (FBS), trypsin, RPMI 1640 medium, and DMEM high-glucose medium. Collagenase IV and DNase I were obtained from Sigma-Aldrich (Germany). Additional materials and their sources: Trypan Blue, paraffin, 4% paraformaldehyde (Guangzhou Xiangbo Biotech Co., Ltd., China); xylene, ethanol, hydrochloride, sodium chloride (Guangzhou Chemical Reagent Factory, China); isoflurane (Shenzhen Ward Life Technology Co., Ltd., China); neutral gum (Shanghai Zhanyun Chemical Co., Ltd., China); murine IFN-γ ELISA kit (R&D Systems, Inc., USA); murine 1× lymphocyte separation medium (Dakewe Biotech Co., Ltd., China); calcium-magnesium-free and phenol red-free HBSS (Wuhan Servicebio Technology Co., Ltd., China); all flow cytometry antibodies (Biolegend, Inc., USA); Percoll isolate (GE Healthcare, USA); ampicillin, metronidazole, neomycin sulfate, vancomycin (Shanghai Mai Lin Biochemical Co., Ltd., China).

### Cell line

The murine colon cancer cell line CT26.WT (catalog number:CL-0071) was acquired from Wuhan Pricella Biotechnology Co., Ltd. (Wuhan, Hubei, China) and maintained in RPMI 1640 medium supplemented with 15% (FBS).

### Animal experiments and ethics statement

Male BALB/c mice (5-week-old; Guangdong Provincial Laboratory Animal Center, Certification No. SCXK(Yue)2022-0002) were housed in the SPF Laboratory Animal Center of Guangdong Pharmaceutical University (20–25°C, 50–70% humidity adequate ventilation) and acclimatized for 7 days before experimentation. All studies were approved by the University’s Animal Ethics Committee (Approval No. gdpulacspf2022020) and conducted in accordance with its Institutional Animal Care and Use Guidelines.

### Preliminary experiment in CT26 tumor-bearing mice

40 Mice were weighed, labeled, and randomly allocated to 8 groups using a random number table with body weight stratification to ensure comparable baseline body weights across groups (n = 5 per group): PAMK groups (125, 250, 500 mg/kg/day, doses), Pre-PAMK groups (125, 250, 500 mg/kg/day doses), ASP (Aspirin, 100 mg/kg/day), Model group (equivalent volume of sterile normal saline, NS), and Control group (equivalent volume of NS). The same grouping method was used in subsequent experiments. On the first 14 days, mice in the Pre-PAMK groups were administered with the corresponding doses of PAMK via oral gavage once daily. On day 15, CT26 cells were harvested, counted and resuspended in serum-free RPMI 1640 medium to a final concentration of 2×10⁷ cells/ml. The same procedure was used for tumor cell preparation and model establishment in subsequent experiments. All groups except the Control group were subcutaneously injected with 0.1 ml of the prepared CT26 cell suspension into the right anterior axillary region to establish syngraft tumors. 2 h later, all mice including the PAMK and ASP groups were administered with the respective doses of PAMK/ASP solution or NS for an additional 14 days. Throughout the experimental period, mouse body weight and tumor volume were measured every 48 h; tumor volume was assessed in a blinded manner to avoid observer bias. Fecal consistency, mobility and feeding behavior were also closely monitored and recorded. Tumor volumes were calculated as length × width^2^ / 2. The same monitoring procedure was applied in subsequent experiments.

### Formal experiment in CT26 tumor-bearing mice

Based on the findings of preliminary experiment (Supplementary Fig. 1), two doses of PAMK (125 mg/kg and 250 mg/kg) were selected to investigate its anti-tumor efficacy in this experiment. A total of 32 mice were randomly assigned to 4 groups (*n* = 8): Pre-PAMK125, Pre-PAMK250, Model group, and Control group. Over the first two weeks (day −14 to day −1), Pre-PAMK125 and Pre-PAMK250 groups were administered the corresponding PAMK doses via oral gavage once daily, while other groups received an equivalent volume of sterile normal saline (0.1 mL/10 g body weight) following the same schedule. On day 0, CT26 cells were harvested, counted and resuspended in serum-free RPMI 1640 medium to a final concentration of 2 × 10⁷ cells/mL. All groups except the Control group were subcutaneously injected with 0.1 mL of the prepared CT26 cell suspension into the right anterior axillary region to establish syngeneic subcutaneous tumors. Subsequently, the respective doses of PAMK solution or sterile normal saline were administered for an additional 14 days. All other operational steps were performed as described above.

Fresh fecal samples were collected from mice in the Control, Model, and Pre-PAM250 groups on the 14th day post-tumor implantation. Briefly, each mouse was individually housed in a sterile cage lined with a sterile pad to absorb urine and prevent sample contamination. Fecal pellets were immediately collected using sterile forceps, transferred to pre-labeled sterile tubes, and stored at -80°C until further processing.

### Experiment in antibiotic-induced microbiota depleted (AIMD) tumor-bearing mice

Based on the findings of the above experiment, one dose of PAMK (250 mg/kg) was selected in this experiment. Before the experiment, an antibiotic cocktail (ABX) was prepared by dissolving the following in 50 mL of normal saline under vigorous agitation: vancomycin (25 mg, 0.5 mg/mL); ampicillin (50 mg, 1 mg/mL); metronidazole (50 mg, 1 mg/mL); neomycin (50 mg, 1 mg/mL)^48,49^. During antibiotic treatment, all mice were administered 0.2 mL of ABX daily via gavage for 10 consecutive days to induce gut microbiota dysbiosis and housed together under identical standardized conditions (4 mice per cage). Fresh fecal samples were collected aseptically, and colony counting was performed using the spread plate technique (as described in the following).

For the experiment, 16 AIMD mice were randomly assigned to two groups (n = 8 per group): one group received PAMK (Pre-PAMK250), while the other received normal saline (Model). To eliminate the homogenization of gut microbiota caused by social contact and coprophagy among animals, all subjects were individually caged according to their treatment groups with consistent housing conditions across all cages. All other operational steps were performed as described above.

### Experiment in AIMD-FMT (fecal microbiota transplantation) tumor-bearing mice

After completion of antibiotic treatment (prepared as described above), 16 AIMD mice were randomly assigned to two groups (*n* = 8 per group) and housed separately by group with consistent housing conditions across all cages (1 mice per cage): one group received PAMK (Pre-PAMK250), while the other received normal saline (Model). On the second day after the last administration of antibiotics, CT26 tumor cells were subcutaneously inoculated into the mice (steps were performed as described above.). Concurrently, mice in the Model group received daily gavage of 0.2 mL fecal microbiota suspension derived from pooled fecs of multiple conventional normal saline-treated CT26-bearing mice, while those in the Pre-PAMK250 group were given the same volume of fecal suspension from pooled feces of multiple Pre-PAMK250-treated CT26-bearing mice; which lasted for 14 days as PAMK or normal saline treatment. Measurements and observations were performed as described above. Preparation of Fecal Microbiota Suspension: Frozen fecal samples were thawed on ice, weighed, and resuspended in sterile 0.9% normal saline at a concentration of 125 mg/mL. The mixture was thoroughly homogenized using a vortex mixer. The resulting suspension was centrifuged at 2300 rpm for 5 min at 4 °C. The upper bacterial supernatant was carefully aspirated, placed on ice, and immediately used for oral gavage.

### Spread plate technique and colony counting

Upon completion of the 10-day ABX gavage regimen, fresh fecal samples were aseptically collected from each mouse as described above. Fecal pellets suspension (125 mg/mL) were prepared as described above centrifuged at 2300 rpm for 5 min at room temperature. The upper bacterial supernatant was gently aspirated to avoid pellet disturbance. Serial dilutions (10⁻¹ to 10⁻⁴) of the bacterial solution were prepared, and 0.1 mL aliquots of each dilution were plated onto sterile nutrient agar plates. The inoculum was evenly spread across the agar surface using a sterile L-shaped spreader, and the plates were incubated in a 37 °C anerobic incubator (Thermo Fisher, USA) for 24 h. Microbiota depletion efficacy was assessed by comparing colony-forming units (CFU) per gram of feces between ABX-treated mice and age-matched normal untreated control mice.

### Experimental endpoint and tissue harvesting

24 h after the final administration, mice were anesthetized with 4% isoflurane, and blood samples were collected via the retro-orbital venous plexus. The collected blood was centrifuged at 3000 rpm for 15 min at 4 °C to obtain serum, which was aliquoted and stored at –80°C until analysis. Following this, all mice were sacrificed via cervical dislocation. Tumors, spleens and thymuses were promptly harvested and weighed. Spleen and thymus indices (organ weight/body weight ×100%) were determined. All harvested tissues were snap-frozen in liquid nitrogen and stored at –80°C.

### Histopathology evaluation via HE staining

Spleen and tumor tissues were harvested and immediately fixed in 4% PFA at 4 °C for 24 h. Tissues were then dehydrated using a graded ethanol series, cleared in xylene, infiltrated with molten paraffin, and embedded in paraffin blocks. Serial sections were cut, mounted onto glass slides, and dried at 45 °C for 2 h. For staining, sections were deparaffinized in xylene (twice for 10 min), rehydrated through a descending ethanol gradient (100% to 70%, 5 min each), and rinsed with distilled water. Sections were stained with hematoxylin for 5 min, differentiated with 1% hydrochloric acid in ethanol for 30 s, rinsed with tap water, counterstained with eosin for 2 min, and dehydrated through an ascending ethanol gradient. After clearing in xylene, sections were mounted with neutral gum and covered with protective coverslips. Morphological evaluation was carried out under an upright fluorescence microscope (Nikon, Japan), and images were captured for analysis.

### Measurement of serum IFN-γ levels by ELISA

Serum IFN-γ was quantified according to the instructions of the specific ELISA kit. A Multiskan FC microplate photometer (Thermo Fisher, USA) was used to measure the absorbance of each well at 450 nm, with a reference wavelength of 630 nm to correct for background noise. IFN-γ concentrations in the samples were calculated by fitting the absorbance values to a standard curve generated with serial dilutions of recombinant IFN-γ standards.

### Flow cytometric analysis of immune cells in tissues

Flow cytometric analysis was carried out using a Attune NxT flow cytometer (Thermo Fisher, USA). Murine tumor and spleen tissues were minced and digested in a solution containing collagenase IV (0.2 mg/mL) and DNase I (0.010 mg/mL) at 37 °C for 60 min. The resulting cell suspensions were filtered through a 200-μm mesh and resuspended in complete high-glucose DMEM (supplemented with 1% BSA and 10% FBS). Aliquots (0.1 mL, 1×10⁷ cells/mL.) were stained with the following antibody panels: splenic NK cells (CD3⁻CD49b⁺) with αCD3-FITC and αCD49b-PE; T cell subsets (CD3⁺CD4⁺/CD8⁺) with αCD3-APC, αCD4-PE, and αCD8a-FITC; and B cells (CD3⁻B220⁺) with αCD3-APC and αB220-AF488. For tumor-infiltrating immune cells, all staining panels included αCD45-PerCP/Cy5.5, with NK cells (CD45⁺CD3⁻CD49b⁺), T cell subsets (CD45⁺CD3⁺CD4⁺/CD8⁺), and B cells (CD45⁺CD3⁻B220⁺) being identified by the addition of the aforementioned spleen-targeting antibody combinations, respectively.

For intracellular granzyme B detection in NK cells (CD3⁻CD49b⁺GranzymeB⁺), surface staining was followed by fixation with 0.25 mL/tube of FluoroFix™ buffer. After permeabilization with 1× permeabilization wash buffer, cells were incubated with an anti-mouse Granzyme B antibody (APC/Fire™ 750, 0.005 mL) for intracellular staining. For natural killer group 2D (NKG2D) expression analysis on NK cells, 1.25× 10⁻³ mL of APC anti-mouse CD314 (NKG2D) antibody was added per tube following surface staining.

### 16S rRNA sequencing for gut microbiota profiling

Mouse fecal samples were retrieved from a − 80 °C freezer and genomic DNA was extracted from mouse fecal samples (stored at −80 °C) using a commercial kit. The V3–V4 hypervariable region of the 16S rRNA gene was amplified by PCR using the primer set 341 F (CCTAYGGGRBGCASCAG) and 806 R (GGACTACNNGGGTATCTAAT). Quantified and qualified PCR products were used to construct sequencing libraries. Paired-end sequencing (250 bp) was performed on an Illumina NovaSeq 6000 platform (Illumina, San Diego, CA, USA).

Raw sequencing reads were quality-filtered using Trimmomatic 0.39. Reads shorter than 200 bp, with an average quality score below 20, or containing ambiguous N bases were removed. High-quality clean tags were clustered into operational taxonomic units (OTUs) at 97% sequence similarity using UPARSE (USEARCH v7). Taxonomic assignment was performed against the SILVA 138.1 SSU rRNA database using Mothur v1.48.

α-Diversity indices (Chao1, Shannon, and Simpson) and β-diversity based on unweighted UniFrac distances were calculated with the phyloseq v1.40.0 and vegan v2.6-2 packages in R v4.2.0. Principal coordinate analysis (PCoA) was performed to reveal β-diversity patterns. The relative abundances of the top 10 phyla and genera were visualized as stacked bar plots. LEfSe analysis (LDA score > 3.8) was used to identify discriminative taxa between groups.

### Integrated metabolomics analysis of fecal metabolites

Mouse fecal samples were retrieved from a − 80 °C freezer and thawed on ice. All subsequent procedures were performed on ice to maintain sample stability. Briefly, 20 mg of each sample was transferred into a sterile centrifuge tube, and 0.40 mL of 70% methanol aqueous solution containing internal standards was added. The mixture was vortexed for 3 min, sonicated for 10 min, vortexed for an additional 1 min, and then incubated at −20 °C for 30 min. After centrifugation at 12,000 rpm for 10 min at 4 °C, 0.30 mL of the supernatant was collected and centrifuged again at 12,000 rpm for 3 min at 4 °C. Finally, 0.20 mL of the supernatant was analyzed using ultra-high-performance liquid chromatography-tandem mass spectrometry (UHPLC-MS/MS).

Quality control (QC) samples were inserted every 12 test samples, and blank samples were interspersed throughout the analysis. Raw mass spectrometry data were converted to mzXML format using ProteoWizard software. Peak extraction, alignment, and retention time correction were performed using the XCMS program. Peak areas were normalized using the support vector regression (SVR) method, and features with a missing value rate >50% were filtered out to obtain reliable metabolite identification information.

The stability and reliability of the analytical method were evaluated using two strategies: overlap analysis of total ion current (TIC) chromatograms and calculation of Pearson correlation coefficients among QC samples. A supervised orthogonal projections to latent structures discriminant analysis (OPLS-DA) model was constructed, and a permutation test with 200 random permutations was performed to validate the effectiveness and robustness of the model. Differential metabolites between groups were identified using the following criteria: |log₂ fold change (log₂FC)| > 1, variable importance in projection (VIP) > 1 derived from OPLS-DA, and *P* < 0.05 by Student’s t-test. The differential metabolites were further subjected to metabolite set enrichment analysis (MSEA), Kyoto Encyclopedia of Genes and Genomes (KEGG) pathway enrichment analysis (*P* < 0.05). Correlation analysis between differential metabolites and gut microbiota was performed using the ComplexHeatmap package in R software, with a correlation coefficient |r | > 0.6 as the cutoff.

### RNA sequencing (RNA-Seq) analysis of murine splenic tissues

Total RNA was isolated from murine splenic tissues with Trizol reagent (Vazyme, R401-01) according to the manufacturer’s instructions. RNA quality and integrity were verified by Qubit 4.0 Fluorometer (Thermo Fisher Scientific, USA) and 1% agarose gel electrophoresis, respectively. Poly(A)+ mRNA was enriched from total RNA using oligo(dT)-coated magnetic beads, then fragmented into 150–200 bp short segments in fragmentation buffer at 94°C for 8 min. The resulting fragments were reverse-transcribed into first-strand cDNA using random hexamer primers, followed by second-strand cDNA synthesis. The double-stranded cDNA was subjected to end repair (filling in sticky ends), 3′ adenylation (adding a single A base), and ligation to Illumina-compatible sequencing adapters. Following PCR amplification (15 cycles) and purification with AMPure XP beads, the resulting ligation products were used to construct a cDNA sequencing library. Library quality was assessed using an Qsep400 Bio-Fragment Analyzer (BiOptic Inc., China), and sequencing was performed on an Illumina NovaSeq 6000 platform (paired-end 150 bp reads). Raw sequencing data were filtered to remove low-quality reads, adapter sequences, and contaminating sequences. Clean reads were mapped to the mouse reference genome (GRCm39) using HISAT2 software, and gene expression levels were quantified as reads per million (RPM) using featureCounts. Differentially expressed genes (DEGs) were identified with DESeq2 software using criteria of |Fold Change | > 1.5 and adjusted P (Padj) < 0.05. GO (Gene Ontology) functional annotation and KEGG pathway enrichment analyses were conducted for DEGs using clusterProfiler R package (*P* < 0.05). Finally, the transcriptomic dataset was integrated with the untargeted metabolomics data for conjoint bioinformatic analysis to reveal potential molecular mechanisms.

### Quantitative Real-Time PCR (RT-qPCR) analysis

Total RNA was extracted from cells using Trizol reagent (Vazyme, R401-01), and 1 μg of RNA was reverse-transcribed into cDNA using the HiScript II Q RT SuperMix kit (Vazyme, R223-01) to remove genomic DNA interference. The synthesized cDNA was diluted 4-fold with nuclease-free water and used as the template for qPCR. Each reaction was performed in triplicate (technical replicates) for each sample, with three independent biological replicates per group. qPCR was conducted on a StepOnePlus Real-Time PCR System (Analytik Jena, Germany) using Taq Pro Universal SYBR qPCR Master Mix (Vazyme, Cat. No. Q712-02) following the kit protocol: 95 °C for 30 s, followed by 40 cycles of 95 °C for 10 seconds and 60 °C for 30 seconds. A melting curve analysis (60–95 °C) was included to confirm amplicon specificity. Relative gene expression levels were calculated using the 2^(-ΔΔCt) method, with β-actin as the housekeeping gene for normalization. Primers sequences were listed in Table 1.

**Table 1:** List of Primers Used in RT-qPCR

Gene	Forward primer (5’-3’)	Reverse primer (5’-3’)
*β-actin*	GTGCTATGTTGCTCTAGACTTCG	ATGCCACAGGATTCCATACC
*Ccl19*	GCTAATGATGCGGAAGACTG	ACTCACATCGACTCTCTAGG
*Cxcl9*	CCTAGTGATAAGGAATGCACGATG	CTAGGCAGGTTTGATCTCCGTTC
*Mmp9*	GGTACTGGAAGATGTCGTGT	TGAAGTCTCAGAAGGTGGAT
*mt-Atp6*	CAGTCCCCTCCCTAGGACTT	TCAGAGCATTGGCCATAGAA
*mt-Co3*	TAGCCTCGTACCAACACATGA	AGTGGTGAAATTCCTGTTGGA
*Per2*	TCATCATTGGGAGGCACAAA	GCATCAGTAGCCGGTGGATT

This study is a preclinical mechanistic study in mice.

CONSORT, PRISMA, and PRISMA‑SCR checklists are not applicable.

### Molecular docking analysis

3D structures of metabolite ligands were downloaded from the PubChem database in SDF format and converted to mol2 format using OpenBabel software (version 2.4.1) for preprocessing (including hydrogen addition and charge calculation). Receptor proteins structures were obtained from the AlphaFold Protein Structure Database (for predicted structures) or RCSB PDB database (for experimentally resolved structures). Using PyMOL (Version 2.4.0), non-specific bound ligands, water molecules, and cofactors were removed from the receptor proteins, and polar hydrogens were added. Molecular docking simulations were conducted with AutoDock Tools (v1.5.7) using the Lamarckian genetic algorithm: the grid box was set to cover the active pocket of the receptor (grid center: X, Y, Z; size: 20×20×20 Å; spacing: 0.375 Å), and 100 independent docking runs were conducted per ligand-receptor pair. The conformation with the lowest binding free energy (kcal/mol) was selected as the optimal docking pose and visualized in PyMOL and Discovery Studio Visualizer (Version 2019) for interaction analysis (e.g., hydrogen bonds, hydrophobic interactions).

### Molecular dynamic simulation

Molecular dynamics (MD) simulation was performed using the YASARA platform. Initial structures of the receptor-ligand complexes were taken from molecular docking results. The simulation system was solvated in a cubic box filled with TIP3P visible water molecules, with a minimum buffer zone of 0.5 nm (5 Å) maintained between the protein surface and the box boundary to avoid periodic image interactions. Simulations employed the Amber14 force field under periodic boundary conditions. Counterions (Na⁺ and Cl⁻) were added at a physiological concentration (0.15 M) to neutralize the net charge of the system. All simulations were carried out at pH 7.4 and 298 K in the NVT (constant number of particles, volume, and temperature) ensemble. Temperature was regulated using a Langevin thermostat with a friction coefficient of 0.1 ps^-1^. Prior to the production run, the system underwent energy minimization (10,000 steps of steepest descent followed by 10,000 steps of conjugate gradient) to relieve steric clashes. The production simulation was performed for 50 ns with an integration time step of 2.5 fs, and trajectory coordinates were saved every 100 ps for subsequent analysis (e.g., root mean square deviation [RMSD], root mean square fluctuation [RMSF]). The binding strength between the receptor and the ligand was estimated using the built-in BindEnergy scoring function in YASARA. The binding energy was assessed by calculating the energy difference between the ligand in the infinitely distant state (unbound state) and the bound state. It was evaluated using the same force field and was reported in calories per mole. The more positive the binding energy is, the more favorable the interaction of the chosen force field is^18,19^.

### Statistical analyses

For animal experiments, outliers were identified using Grubs’ test. Mice with severe weight loss (>20% of initial body weight), tumor ulceration or necrosis, or severe clinical symptoms were excluded from the analysis together with all related data. Missing data was handled by complete case analysis without imputation. All quantitative data were expressed as the mean ± standard error of the mean (S.E.M.). For comparisons between two groups, Student’s t‑test or Mann‑Whitney U test was used accordingly. For three or more groups, one‑way ANOVA with Dunnett’s post‑test was applied for data with normal distribution and equal variances; Welch’s ANOVA with Dunnett’s T3 was used for unequal variances, and Kruskal‑Wallis with Dunn’s post‑test for non‑parametric data. For repeated measures over time, a mixed‑effects model with Geisser‑Greenhouse correction was employed. Post hoc comparisons versus the Model group were corrected for false discovery rate using the Benjamini‑Krieger‑Yekutieli procedure (FDR = 0.05). All statistical analyses were performed using GraphPad Prism software (version 10.0) or the R programming language (version 4.2.0). Statistical significance was defined as follows: ^#^/*, *P* < 0.05; ^##^/**, *P* < 0.01; ^###^/***, *P* < 0.001. ^#^ vs. Control group; * vs. Model group.

## Supplementary information

Supplementary Information

## Data Availability

The minimal dataset required to interpret and replicate the findings of this study, including group-level summarized animal experimental data and statistical results, sample-level abundance matrices of differential taxa from 16S rRNA sequencing, sample-level quantitative data of differential metabolites from fecal metabolomics, and sample-level expression matrices of differential genes from spleen transcriptome, are provided in the supplementary tables. Raw sequencing reads of 16S rRNA and spleen transcriptome, as well as full mass spectrometry raw files, are not publicly available due to the complexity of raw data organization and formatting for public deposition, but are available from the corresponding author upon reasonable academic request.
