# Supplementary material for: Atractylodes macrocephala polysaccharide orchestrates anti-tumor immunity via a dual-network mechanism targeting the gut microbiota and spleen
Source: NPJ Biofilms Microbiomes. 2026 Jun 26;12:125. doi: 10.1038/s41522-026-01013-8 (PMC13309544; doi:10.1038/s41522-026-01013-8)
Supplement: Supplementary file 1 — Supplementary Information [file 41522_2026_1013_MOESM1_ESM.pdf]

Atractylodes macrocephala Polysaccharide Orchestrates Anti-Tumor Immunity via a Dual-Network Mechanism Targeting the Gut Microbiota and Spleen

[Author Names]

Yajing Shuai[a, #], Jieyu Xing[a, #], Xiaoxian Liu[a], Zijing Song[a], Siqi Lin[a], Chengyu Lu[b, *], Weiwei Zeng[c, *], Guixiang Wang[a, *]

[a] School of Pharmacy, Guangdong Pharmaceutical University, Guangzhou 510006, Guangdong, China

[b] Department of Gynecology, Shenzhen Hospital of Southern Medical University, Shenzhen 518112, Guangdong, China

[c] Department of Pharmacy, Shenzhen Longgang Second People's Hospital, Shenzhen 518112, Guangdong, China

[#] These authors contributed equally to this work.

[*] Corresponding author

Chengyu Lu, Department of Gynecology, Shenzhen Hospital of Southern Medical University, Shenzhen 518112, Guangdong, China

E-mail address: Luchengyu1833@163.com

Weiwei Zeng, Department of Pharmacy, Shenzhen Longgang Second People's Hospital, No.175, Jihua Road, Longgang District, ShenZhen, 518112, China.

E-mail address: zwwspring@126.com

Guixiang Wang, School of Pharmacy, Guangdong Pharmaceutical University, 280 Wai Huan Dong Road, Guangzhou 510006, China

E-mail address: wgx306@126.com

SUPPLEMENTARY MATERIAL

PAMK showed tumor growth inhibitory effects in CT26 tumor-bearing mice

In this experiment, two different administration timings were designed to assess the therapeutic efficacy of PAMK: one received treatment starting at the time of tumor inoculation (PAMK groups), and the other received treatment from 14 days prior to tumor inoculation (Pre-PAMK groups). Overall, compared with the Model group, tumor growth was markedly suppressed in most PAMK-treated mice and positive control group (ASP), and the Pre-PAMK groups exhibited better inhibitory effects than the PAMK groups. At the same dose, the final tumor volume and weight in the Pre-PAMK groups were smaller and lighter than the PAMK groups (**Supplementary Fig. 1a-c)**, respectively, indicating that the Pre-PAMK intervention had a better effect. Specifically, the Pre-PAMK250 group (250 mg/kg) performed better than the Pre-PAMK125 group (125 mg/kg), and although most mice in the 500 mg/kg group responded better than the 250 mg/kg group, some individuals in this group showed no intervention effect, resulting in an overall weaker efficacy in the 500 mg/kg group compared with the 250 mg/kg group and even 125 mg/kg group (**Supplementary Fig. 1**a-c).

In terms of body weight (**Supplementary Fig. 1d**), mice in the Control group gained weight steadily over time, the Model group showed a similar pattern to the control group in the early period, but weight gain plateaued and even slightly decreased in the later stage of the experiment (D8–D14) due to increased tumor burden. Mice in the ASP group exhibited consistently lower body weight than both Control and Model groups, with slow weight fluctuation during D–6 to D0, and no further weight gain in the late tumor stage (D8–D14), similar to the Model group. All PAMK groups gained weight comparably to the Control and Model groups before tumor inoculation, but weight gain ceased after tumor development. Specifically, the PAMK125 group showed a slight weight reduction during D8–D14 due to large tumor burden, resembling the Model group. The PAMK250 group maintained body weight between the model and control groups, whereas the PAMK500 and PAMK125 groups exhibited curves nearly overlapping with the Model group. At the same dose, all Pre-PAMK groups showed higher body weights than the corresponding PAMK groups. Pre-PAMK125 and

Pre-PAMK250 groups remained close to the Control group throughout the experiment, with Pre-PAMK125 even exceeding the Control. The Pre-PAMK500 group displayed considerable fluctuations, with transient body weight loss observed after the first administration and after tumor inoculation (D–12, D4), followed by recovery. Statistical analysis revealed no significant differences in body weight among all groups (all $P > 0.05$ vs. Control).

In addition, compared with the Control group, the thymus index of mice in the Model group was significantly decreased ($P<0.01$, **Supplementary Fig. 1e**), which was attributed to tumor-induced systemic immunosuppression and immune organ atrophy. Following treatment with PAMK or ASP, the thymus index increased, but the improvement of PAMK, Pre-PAMK groups and ASP varied greatly among groups (all $P > 0.05$ vs. Control). Compared with the Control group, the spleen index of mice in the Model group was significantly increased ($P<0.05$,), as commonly observed in tumor-bearing hosts. Following treatment with PAMK or ASP, the thymus index decreased, and the improvement of PAMK, Pre-PAMK groups and ASP varied greatly among groups, only the Pre-PAMK250 group showing the most pronounced effect ($P<0.05$, **Supplementary Fig. 1f**).

These results indicate that PAMK exerted certain inhibitory effects on colon cancer growth in CT26 tumor-bearing mice. PAMK treatments tended to alleviate tumor-burden-induced alterations in body weight and thymus index, and spleen index. Notably, pre-treatment with PAMK (Pre-PAMK125 and Pre-PAMK250) produced more favorable effects and warrants further investigation.

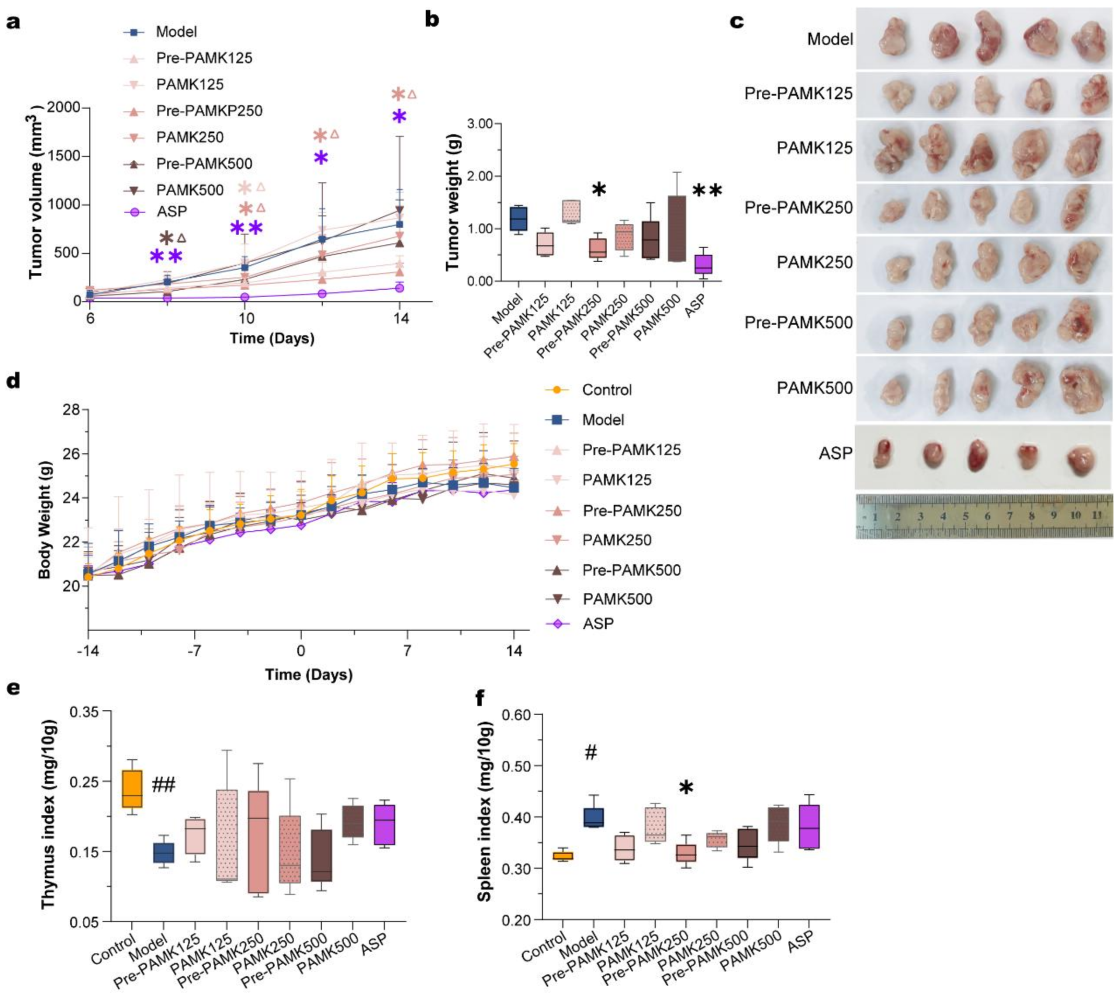

Supplementary Fig. 1 Polysaccharide of *Atractylodes macrocephala Koidz* (PAMK) showed tumor growth inhibitory effects in CT26 tumor-bearing mice (n = 5). **a.** Mice tumor volume changes; **b**. Tumor weight; **c.** Image of mice-derived tumor tissues; **d.** Mice body weight changes during the experiment; **e.** Thymus indices of mice; **f.** Spleen indices of mice. Statistical significance is indicated as: $^{\#}$/*, $P < 0.05$; $^{\#\#}$/**, $P < 0.01$; $^{\#\#\#}$/***, $P < 0.001$. $^{\#}$ vs. Control, * vs. Model.

Characterization of PAMK

1. Purity

- **Total carbohydrate content**: Determined by the phenol-sulfuric acid method using D-glucose as the standard. The total carbohydrate content of PAMK was 94.82%.
- **Protein content**: Measured by BCA assay with bovine serum albumin (BSA) as the standard. The protein content of PAMK was 4.2%, indicating low protein contamination.
- **Endotoxin**：0.01907 EU/mg (Limulus Amebocyte Lysate, LAL Test).

2. Characterization of structure

- **UV-Vis spectroscopy**: Result showed no characteristic absorption peaks at 260 nm or 280 nm.
- **FT-IR spectroscopy**: The FT-IR spectrum of PAMK showed characteristic absorption peaks of polysaccharides：3356.13 cm^{-1} (O-H stretching vibration), 2935.65 cm^{-1} (C-H stretching vibration), 1639.49 cm^{-1} (C=O stretching vibrations of uronic acids), 1130.28 cm^{-1} (C-O-H vibrations), 1029.98 cm^{-1} (pyranose ring characteristics), 937.40 cm^{-1} (C-O-C asymmetric vibrations), and 821.67 cm^{-1} (α/β-type glycosidic linkage).
- **Monosaccharide composition (**by Ion Chromatography**)**: The results showed that PAMK was composed of fructose (95.22%), glucose (3.99%), arabinose (0.61%), and xylose (0.18%).

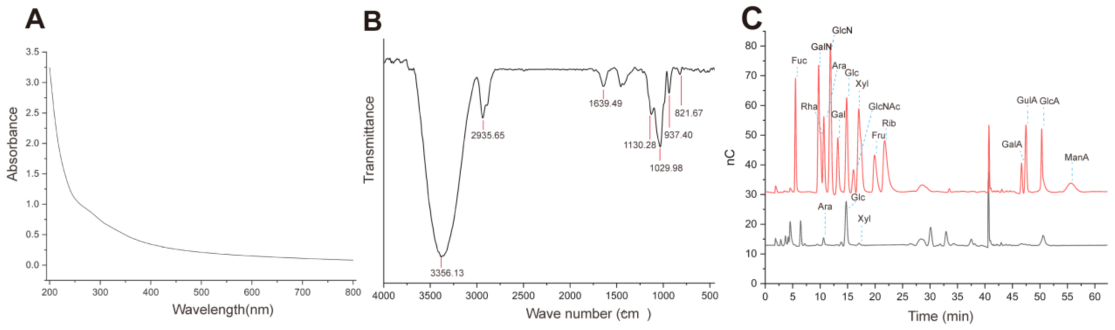

Characterization of PAMK. (A) UV-Vis absorption spectrum; (B) Fourier-transform infrared spectroscopy (FTIR) spectrum; (C) Ion chromatographic profile of monosaccharide composition, with the upper panel showing the standard curve and the lower panel showing the sample chromatogram.

Data Availability

The data presented herein are from another unpublished manuscript by our research group. but the data are available from the corresponding author on reasonable request.

statistic report:

Fig. S1 a

Group1 vs Group 2	Mean 1	Mean 2	Mean Diff.	95% CI of diff.	SE of diff.	N1	N2	t	DF	Individual P Value	Effectsize(Cohen's d)	Summary
**D6**												
Pre-PAMK125 vs. Model	68.06	74.18	-6.118	-58.46–46.23	20.14	5	5	0.3038	4.6600	0.7744	0.28	ns
PAMK125 vs. Model	90.98	74.18	16.8	-41.91 ~ 75.51	20.71	5	5	0.8113	5.1370	0.4531	0.72	ns
Pre-PAMKP250 vs.	84.12	74.18	9.941	-44.44 ~ 64.32	20.18	5	5	0.4925	4.6970	0.6445	0.45	ns
PAMK250 vs. Model	118.6	74.18	44.4	-14.09 ~ 102.89	25.2	5	5	1.762	7.7500	0.1173	1.27	ns
Pre-PAMK500 vs. Model	58.54	74.18	-15.64	-69.51 ~ 38.23	19.83	5	5	0.7887	4.3970	0.4707	0.75	ns
PAMK500 vs. Model	99.38	74.18	25.21	-29.54 ~ 79.96	22.71	5	5	1.11	6.6440	0.3057	0.86	ns
ASP vs. Model	37.42	74.18	-36.76	-91.01 ~ 17.49	20.1	5	5	1.829	4.6240	0.1317	1.70	ns
**D8**												
Pre-PAMK125 vs. Model	144	204.7	-60.7	-144.99 ~ 23.59	35.87	5	5	1.692	6.1400	0.1404	1.37	ns
PAMK125 vs. Model	229.9	204.7	25.15	-89.99 ~ 140.29	49.92	5	5	0.5037	7.6930	0.6286	0.36	ns
Pre-PAMKP250 vs.	129.6	204.7	-75.11	-160.61 ~ 10.39	34.75	5	5	2.161	5.6160	0.077	1.82	ns
PAMK250 vs. Model	183.1	204.7	-21.64	-104.11 ~ 60.83	34.53	5	5	0.6268	5.5080	0.5559	0.53	ns
Pre-PAMK500 vs. Model	105.1	204.7	-99.62	-183.71 ~ -15.53	33.49	5	5	2.975	4.9800	0.0311	2.67	*
PAMK500 vs. Model	191.2	204.7	-13.53	-157.94 ~ 130.88	62.14	5	5	0.2177	6.4840	0.8343	0.17	ns
ASP vs. Model	38.72	204.7	-166	-254.95 ~ -77.05	32.12	5	5	5.169	4.2750	0.0055	5.00	**
**D10**												
Pre-PAMK125 vs. Model	196.1	353.7	-157.6	-301.94 ~ -13.26	55	5	5	2.865	5.5800	0.031	2.43	*
PAMK125 vs. Model	400.1	353.7	46.36	-202.11 ~ 294.83	100.4	5	5	0.4617	6.3890	0.6596	0.37	ns
Pre-PAMKP250 vs.	171.8	353.7	-181.9	-329.94 ~ -33.86	54.15	5	5	3.359	5.3130	0.0183	2.91	*
PAMK250 vs. Model	253.3	353.7	-100.4	-255.94 ~ 55.14	66.98	5	5	1.5	7.8890	0.1726	1.07	ns
Pre-PAMK500 vs. Model	231.6	353.7	-122.1	-328.99 ~ 84.79	88.91	5	5	1.374	7.0580	0.2115	1.03	ns
PAMK500 vs. Model	400	353.7	46.27	-342.10 ~ 434.64	142.9	5	5	0.3239	5.1000	0.7589	0.29	ns
ASP vs. Model	48.18	353.7	-305.5	-436.50 ~ -174.50	50.94	5	5	5.998	4.2730	0.0031	5.80	**
**D12**												
Pre-PAMK125 vs. Model	305.2	648.4	-343.2	-740.77 ~ 54.37	147.7	5	5	2.323	4.8900	0.069	2.10	ns
PAMK125 vs. Model	739.9	648.4	91.57	-294.11 ~ 477.25	161.9	5	5	0.5657	6.4110	0.5908	0.45	ns
Pre-PAMKP250 vs.	230.7	648.4	-417.7	-812.94 ~ -22.46	143	5	5	2.92	4.3420	0.0391	2.80	*
PAMK250 vs. Model	484.4	648.4	-164	-550.11 ~ 222.11	168.5	5	5	0.9729	6.9830	0.3631	0.74	ns
Pre-PAMK500 vs. Model	467.7	648.4	-180.7	-710.00 ~ 348.60	233.3	5	5	0.7746	7.4220	0.4625	0.57	ns
PAMK500 vs. Model	625.5	648.4	-22.89	-762.74 ~ 716.96	303.4	5	5	0.07545	6.0190	0.9423	0.06	ns
ASP vs. Model	84.42	648.4	-564	-989.55 ~ -138.45	140.6	5	5	4.011	4.0610	0.0155	3.98	*
**D14**												
Pre-PAMK125 vs. Model	395.4	798.4	-402.9	-842.11 ~ 36.31	165.2	5	5	2.439	4.3820	0.0658	2.33	ns
PAMK125 vs. Model	866.2	798.4	67.81	-414.52 ~ 550.14	200.1	5	5	0.3389	7.3360	0.7442	0.25	ns
Pre-PAMKP250 vs.	309.6	798.4	-488.7	-944.04 ~ -33.36	166.2	5	5	2.941	4.4790	0.0368	2.78	*
PAMK250 vs. Model	678.4	798.4	-120	-600.64 ~ 360.64	213.4	5	5	0.5623	7.8360	0.5897	0.40	ns
Pre-PAMK500 vs. Model	609.1	798.4	-189.2	-770.12 ~ 391.72	255.8	5	5	0.7399	7.6820	0.4813	0.53	ns
PAMK500 vs. Model	943.9	798.4	145.5	-842.14 ~ 1133.14	377.7	5	5	0.3853	5.7020	0.714	0.32	ns
ASP vs. Model	141.7	798.4	-656.7	-1139.15 ~ -174.25	163.8	5	5	4.01	4.2360	0.0143	3.90	*

Fig. S1 b

Group1 vs Group 2	Mean 1	Mean 2	Mean Diff.	95% CI of diff.	SE of diff.	n1	n2	t	DF	Adjusted P Value	Effectsize (Cohen's d)	Summary
Model vs. Pre-PAMK125	1.186	0.7033	0.4823	-0.02217 to 0.9868	0.146	5	5	3.303	7.997	0.0619	2.34	ns
Model vs. PAMK125	1.186	1.292	-0.1061	-0.6085 to 0.3963	0.1454	5	5	0.7297	7.994	0.98	0.52	ns
Model vs. Pre-PAMK250	1.186	0.6137	0.5719	0.08852 to 1.055	0.1399	5	5	4.087	7.907	0.0209	2.91	*
Model vs. PAMK250	1.186	0.8568	0.3288	-0.2193 to 0.8770	0.1587	5	5	2.072	7.851	0.3416	1.48	ns
Model vs. Pre-PAMK500	1.186	0.7885	0.3971	-0.4290 to 1.223	0.2192	5	5	1.812	6.151	0.4856	1.46	ns
Model vs. PAMK500	1.186	0.9147	0.2709	-1.108 to 1.650	0.3409	5	5	0.7948	4.816	0.9638	0.72	ns
Model vs. ASP	1.186	0.3065	0.8791	0.3837 to 1.374	0.1434	5	5	6.13	7.975	0.0018	4.34	**

Fig. S1 d

Group1 vs Group 2	Mean 1	Mean 2	Mean Diff.	95% CI of diff.	SE of diff.	N1	N2	t	DF	Individual P Value	Effectsize (Cohen's d)	Summary
D-14												
Model vs. Control	20.56	20.42	0.14	-1.2999 to 1.5799	0.6731	5	5	0.208	5.424	0.8428	0.18	ns
Model vs. Pre-PAMK125	20.56	20.56	0	-2.3898 to 2.3898	1.119	5	5	0	6.947	>0.9999	0.00	ns
Model vs. PAMK125	20.56	20.54	0.02	-1.4498 to 1.4898	0.7101	5	5	0.02817	6.309	0.9784	0.02	ns
Model vs. Pre-PAMK250	20.56	20.52	0.04	-1.6497 to 1.7297	0.8343	5	5	0.04795	7.922	0.9629	0.03	ns
Model vs. PAMK250	20.56	20.54	0.02	-1.5201 to 1.5601	0.7511	5	5	0.02663	7.099	0.9795	0.02	ns
Model vs. Pre-PAMK500	20.56	20.48	0.08	-1.5299 to 1.6899	0.7855	5	5	0.1018	7.564	0.9215	0.07	ns
Model vs. PAMK500	20.56	20.66	-0.1	-1.5799 to 1.3799	0.7044	5	5	0.142	6.183	0.8916	0.11	ns
Model vs. ASP	20.56	20.48	0.08	-1.3498 to 1.5098	0.6488	5	5	0.1233	4.795	0.9069	0.11	ns
D-12												
Model vs. Control	21.14	20.8	0.34	-1.1199 to 1.7999	0.6875	5	5	0.4946	5.827	0.639	0.41	ns
Model vs. Pre-PAMK125	21.14	21.44	-0.3	-3.1399 to 2.5399	1.322	5	5	0.227	6.068	0.8279	0.18	ns
Model vs. PAMK125	21.14	21.02	0.12	-1.3498 to 1.5898	0.7099	5	5	0.169	6.347	0.871	0.13	ns
Model vs. Pre-PAMK250	21.14	21.52	-0.38	-1.9598 to 1.1998	0.7772	5	5	0.489	7.492	0.6389	0.36	ns
Model vs. PAMK250	21.14	21.12	0.02	-1.4899 to 1.5299	0.7355	5	5	0.02719	6.863	0.9791	0.02	ns
Model vs. Pre-PAMK500	21.14	20.52	0.62	-0.9598 to 2.1998	0.7861	5	5	0.7887	7.592	0.4542	0.57	ns
Model vs. PAMK500	21.14	20.88	0.26	-1.2599 to 1.7799	0.7523	5	5	0.3456	7.15	0.7396	0.26	ns
Model vs. ASP	21.14	20.68	0.46	-0.9998 to 1.9198	0.6473	5	5	0.7106	4.799	0.5103	0.65	ns
D-10												
Model vs. Control	21.8	21.48	0.32	-0.7999 to 1.4399	0.5535	5	5	0.5781	7.093	0.5811	0.43	ns
Model vs. Pre-PAMK125	21.8	21.9	-0.1	-2.6199 to 2.4199	1.196	5	5	0.08359	5.322	0.9364	0.07	ns
Model vs. PAMK125	21.8	21.88	-0.08	-1.4898 to 1.3298	0.6895	5	5	0.116	7.877	0.9105	0.08	ns
Model vs. Pre-PAMK250	21.8	22.1	-0.3	-1.5498 to 0.9498	0.6156	5	5	0.4873	7.924	0.6392	0.35	ns
Model vs. PAMK250	21.8	21.4	0.4	-1.0199 to 1.8199	0.7021	5	5	0.5697	7.809	0.5849	0.41	ns
Model vs. Pre-PAMK500	21.8	21.02	0.78	-0.7899 to 2.3499	0.7819	5	5	0.9975	7.259	0.3506	0.74	ns
Model vs. PAMK500	21.8	21.18	0.62	-0.5699 to 1.8099	0.5936	5	5	1.044	7.748	0.3278	0.75	ns
Model vs. ASP	21.8	21	0.8	-0.3199 to 1.9199	0.5235	5	5	1.528	6.306	0.1749	1.22	ns
D-8												
Model vs. Control	22.22	22.06	0.16	-0.7199 to 1.0399	0.4359	5	5	0.3671	7.82	0.7233	0.26	ns
Model vs. Pre-PAMK125	22.22	22.56	-0.34	-2.7599 to 2.0799	1.158	5	5	0.2935	4.704	0.7816	0.27	ns
Model vs. PAMK125	22.22	22.36	-0.14	-1.4099 to 1.1299	0.6164	5	5	0.2271	6.78	0.827	0.17	ns
Model vs. Pre-PAMK250	22.22	22.62	-0.4	-1.5699 to 0.7699	0.5637	5	5	0.7096	7.292	0.5	0.53	ns
Model vs. PAMK250	22.22	21.62	0.6	-0.3799 to 1.5799	0.4856	5	5	1.236	7.959	0.2518	0.88	ns
Model vs. Pre-PAMK500	22.22	21.74	0.48	-1.2599 to 2.2199	0.8343	5	5	0.5754	5.442	0.588	0.49	ns
Model vs. PAMK500	22.22	22.16	0.06	-1.0299 to 1.1499	0.5404	5	5	0.111	7.527	0.9145	0.08	ns
Model vs. ASP	22.22	21.76	0.46	-0.4599 to 1.3799	0.455	5	5	1.011	7.974	0.3417	0.72	ns
D-6												
Model vs. Control	22.74	22.52	0.22	-0.9699 to 1.4099	0.5916	5	5	0.3719	7.998	0.7196	0.26	ns
Model vs. Pre-PAMK125	22.74	22.82	-0.08	-2.4299 to 2.2699	1.125	5	5	0.0711	5.232	0.946	0.06	ns
Model vs. PAMK125	22.74	22.68	0.06	-1.2199 to 1.3399	0.634	5	5	0.09463	7.843	0.927	0.07	ns
Model vs. Pre-PAMK250	22.74	22.84	-0.1	-1.3499 to 1.1499	0.6182	5	5	0.1618	7.926	0.8755	0.11	ns
Model vs. PAMK250	22.74	22.46	0.28	-1.0599 to 1.6199	0.6597	5	5	0.4244	7.672	0.6829	0.31	ns
Model vs. Pre-PAMK500	22.74	22.34	0.4	-1.1999 to 1.9999	0.7831	5	5	0.5108	6.717	0.6258	0.39	ns
Model vs. PAMK500	22.74	22.44	0.3	-1.0399 to 1.6399	0.6589	5	5	0.4553	7.677	0.6615	0.33	ns
Model vs. ASP	22.74	22.1	0.64	-0.3199 to 1.5999	0.4632	5	5	1.382	5.838	0.2177	1.14	ns

D-4												
Model vs. Control	22.88	22.82	0.06	-0.9399 to 1.0599	0.4958	5	5	0.121	7.416	0.9069	0.09	ns
Model vs. Pre-PAMK125	22.88	23.22	-0.34	-2.2399 to 1.5599	0.9316	5	5	0.365	4.896	0.7304	0.33	ns
Model vs. PAMK125	22.88	23.08	-0.2	-1.2999 to 0.8999	0.5393	5	5	0.3709	6.934	0.7218	0.28	ns
Model vs. Pre-PAMK250	22.88	23.3	-0.42	-1.4299 to 0.5899	0.4944	5	5	0.8496	7.431	0.4221	0.62	ns
Model vs. PAMK250	22.88	22.66	0.22	-0.9899 to 1.4299	0.5908	5	5	0.3724	6.434	0.7216	0.29	ns
Model vs. Pre-PAMK500	22.88	22.7	0.18	-1.2299 to 1.5899	0.6785	5	5	0.2653	5.799	0.8	0.22	ns
Model vs. PAMK500	22.88	22.96	-0.08	-1.3899 to 1.2299	0.634	5	5	0.1262	6.089	0.9037	0.10	ns
Model vs. ASP	22.88	22.42	0.46	-0.5499 to 1.4699	0.4968	5	5	0.9259	7.404	0.3837	0.68	ns
D-2												
Model vs. Control	23.02	23.04	-0.02	-1.2099 to 1.1699	0.584	5	5	0.03425	7.341	0.9736	0.03	ns
Model vs. Pre-PAMK125	23.02	23.3	-0.28	-2.0899 to 1.5299	0.9008	5	5	0.3108	5.34	0.7677	0.27	ns
Model vs. PAMK125	23.02	23.1	-0.08	-1.4199 to 1.2599	0.6598	5	5	0.1212	6.645	0.9071	0.09	ns
Model vs. Pre-PAMK250	23.02	23.52	-0.5	-1.5599 to 0.5599	0.5261	5	5	0.9504	7.852	0.3703	0.68	ns
Model vs. PAMK250	23.02	22.86	0.16	-0.9599 to 1.2799	0.5568	5	5	0.2874	7.599	0.7815	0.21	ns
Model vs. Pre-PAMK500	23.02	22.94	0.08	-1.4199 to 1.5799	0.7335	5	5	0.1091	6.11	0.9166	0.09	ns
Model vs. PAMK500	23.02	23.22	-0.2	-1.4799 to 1.0799	0.6299	5	5	0.3175	6.905	0.7602	0.24	ns
Model vs. ASP	23.02	22.58	0.44	-0.6099 to 1.4899	0.5223	5	5	0.8424	7.878	0.4244	0.60	ns
D0												
Model vs. Control	23.22	23.22	0	-1.1999 to 1.1999	0.5778	5	5	0	5.747	>0.9999	0.00	ns
Model vs. Pre-PAMK125	23.22	23.54	-0.32	-1.9499 to 1.3099	0.788	5	5	0.4061	4.883	0.7019	0.37	ns
Model vs. PAMK125	23.22	23.24	-0.02	-1.0999 to 1.0599	0.5301	5	5	0.03773	6.112	0.9711	0.03	ns
Model vs. Pre-PAMK250	23.22	23.78	-0.56	-1.5899 to 0.4699	0.5077	5	5	1.103	6.318	0.3103	0.88	ns
Model vs. PAMK250	23.22	23.14	0.08	-0.7799 to 0.9399	0.4231	5	5	0.1891	7.328	0.8552	0.14	ns
Model vs. Pre-PAMK500	23.22	23.28	-0.06	-1.4899 to 1.3699	0.6898	5	5	0.08698	5.181	0.9339	0.08	ns
Model vs. PAMK500	23.22	23	0.22	-0.9099 to 1.3499	0.5571	5	5	0.3949	5.893	0.7068	0.33	ns
Model vs. ASP	23.22	22.76	0.46	-0.3299 to 1.2499	0.3912	5	5	1.176	7.737	0.2745	0.85	ns
D2												
Model vs. Control	23.62	23.88	-0.26	-1.3899 to 0.8699	0.5485	5	5	0.4741	5.616	0.6533	0.40	ns
Model vs. Pre-PAMK125	23.62	23.8	-0.18	-2.0499 to 1.6899	0.8997	5	5	0.2001	4.551	0.8501	0.19	ns
Model vs. PAMK125	23.62	23.44	0.18	-0.7999 to 1.1599	0.4806	5	5	0.3745	6.161	0.7206	0.30	ns
Model vs. Pre-PAMK250	23.62	24.22	-0.6	-1.9699 to 0.7699	0.6571	5	5	0.9131	5.084	0.4024	0.81	ns
Model vs. PAMK250	23.62	23.54	0.08	-0.5999 to 0.7599	0.3421	5	5	0.2339	7.914	0.821	0.17	ns
Model vs. Pre-PAMK500	23.62	23.6	0.02	-1.3899 to 1.4299	0.6851	5	5	0.02919	4.99	0.9778	0.03	ns
Model vs. PAMK500	23.62	23.26	0.36	-0.8699 to 1.5899	0.6058	5	5	0.5943	5.297	0.5768	0.52	ns
Model vs. ASP	23.62	23.28	0.34	-0.3199 to 0.9999	0.3283	5	5	1.036	7.994	0.3307	0.73	ns
D4												
Model vs. Control	24.18	24.24	-0.06	-1.4099 to 1.2899	0.6648	5	5	0.09025	7.24	0.9305	0.07	ns
Model vs. Pre-PAMK125	24.18	24.68	-0.5	-2.2999 to 1.2999	0.896	5	5	0.558	5.738	0.5979	0.47	ns
Model vs. PAMK125	24.18	23.76	0.42	-0.4799 to 1.3199	0.445	5	5	0.9439	6.353	0.3797	0.75	ns
Model vs. Pre-PAMK250	24.18	24.6	-0.42	-1.7299 to 0.8899	0.6468	5	5	0.6493	7.396	0.5358	0.48	ns
Model vs. PAMK250	24.18	23.9	0.28	-0.7199 to 1.2799	0.4964	5	5	0.5641	7.654	0.5888	0.41	ns
Model vs. Pre-PAMK500	24.18	23.44	0.74	-0.8699 to 2.3499	0.8068	5	5	0.9172	6.189	0.3934	0.74	ns
Model vs. PAMK500	24.18	23.52	0.66	-0.5699 to 1.8899	0.6098	5	5	1.082	7.703	0.3118	0.78	ns
Model vs. ASP	24.18	23.84	0.34	-0.6399 to 1.3199	0.4827	5	5	0.7044	7.409	0.5027	0.52	ns
D6												
Model vs. Control	24.38	24.86	-0.48	-1.6399 to 0.6799	0.5612	5	5	0.8552	5.503	0.4281	0.73	ns
Model vs. Pre-PAMK125	24.38	25.04	-0.66	-2.0899 to 0.7699	0.6863	5	5	0.9617	4.965	0.3807	0.86	ns
Model vs. PAMK125	24.38	24.14	0.24	-0.5099 to 0.9899	0.3728	5	5	0.6437	7.492	0.539	0.47	ns
Model vs. Pre-PAMK250	24.38	25.12	-0.74	-1.6699 to 0.1899	0.4569	5	5	1.619	6.361	0.1537	1.28	ns
Model vs. PAMK250	24.38	24.18	0.2	-1.0799 to 1.4799	0.6291	5	5	0.3179	5.168	0.763	0.28	ns
Model vs. Pre-PAMK500	24.38	23.9	0.48	-1.0799 to 2.0399	0.7572	5	5	0.6339	4.78	0.5552	0.58	ns
Model vs. PAMK500	24.38	23.98	0.4	-0.6299 to 1.4299	0.5018	5	5	0.7971	5.925	0.4561	0.65	ns
Model vs. ASP	24.38	23.82	0.56	-0.3699 to 1.4899	0.4481	5	5	1.25	6.461	0.2548	0.98	ns
D8												
Model vs. Control	24.7	24.9	-0.2	-1.3899 to 0.9899	0.5874	5	5	0.3405	6.213	0.7447	0.27	ns
Model vs. Pre-PAMK125	24.7	25.08	-0.38	-2.0799 to 1.3199	0.8375	5	5	0.4537	5.013	0.669	0.41	ns
Model vs. PAMK125	24.7	24.4	0.3	-0.5199 to 1.1199	0.4099	5	5	0.7319	7.982	0.4851	0.52	ns
Model vs. Pre-PAMK250	24.7	25.48	-0.78	-1.9799 to 0.4199	0.5886	5	5	1.325	6.204	0.2318	1.06	ns
Model vs. PAMK250	24.7	24.54	0.16	-0.6099 to 0.9299	0.3855	5	5	0.4151	7.953	0.6891	0.29	ns
Model vs. Pre-PAMK500	24.7	24.32	0.38	-1.4299 to 2.1899	0.8907	5	5	0.4266	4.886	0.6878	0.39	ns
Model vs. PAMK500	24.7	23.94	0.76	-0.5999 to 2.1199	0.6698	5	5	1.135	5.658	0.3023	0.95	ns
Model vs. ASP	24.7	24.32	0.38	-0.5299 to 1.2899	0.4521	5	5	0.8405	7.64	0.4261	0.61	ns
D10												
Model vs. Control	24.58	25.16	-0.58	-1.7099 to 0.5499	0.5577	5	5	1.04	7.653	0.3301	0.75	ns
Model vs. Pre-PAMK125	24.58	25.34	-0.76	-2.4499 to 0.9299	0.8355	5	5	0.9097	5.628	0.4003	0.77	ns
Model vs. PAMK125	24.58	24.3	0.28	-0.7199 to 1.2799	0.4964	5	5	0.5641	8	0.5882	0.40	ns
Model vs. Pre-PAMK250	24.58	25.52	-0.94	-2.2899 to 0.4099	0.6448	5	5	1.458	6.843	0.1892	1.11	ns
Model vs. PAMK250	24.58	24.9	-0.32	-1.3299 to 0.6899	0.5044	5	5	0.6344	7.989	0.5435	0.45	ns
Model vs. Pre-PAMK500	24.58	24.72	-0.14	-1.9299 to 1.6499	0.8836	5	5	0.1584	5.438	0.8798	0.14	ns
Model vs. PAMK500	24.58	24.44	0.14	-1.2099 to 1.4899	0.6686	5	5	0.2094	6.641	0.8404	0.16	ns
Model vs. ASP	24.58	24.34	0.24	-1.0299 to 1.5099	0.6293	5	5	0.3814	6.982	0.7143	0.29	ns
D12												
Model vs. Control	24.68	25.3	-0.62	-1.8999 to 0.6599	0.6351	5	5	0.9762	7.6	0.359	0.71	ns
Model vs. Pre-PAMK125	24.68	25.48	-0.8	-2.6899 to 1.0899	0.9278	5	5	0.8623	5.681	0.4234	0.72	ns
Model vs. PAMK125	24.68	24.34	0.34	-0.7099 to 1.3899	0.5244	5	5	0.6484	7.867	0.5352	0.46	ns
Model vs. Pre-PAMK250	24.68	25.7	-1.02	-2.2699 to 0.2299	0.6224	5	5	1.639	7.699	0.1414	1.18	ns
Model vs. PAMK250	24.68	24.96	-0.28	-1.3399 to 0.7799	0.5301	5	5	0.5282	7.911	0.6118	0.38	ns
Model vs. Pre-PAMK500	24.68	25.1	-0.42	-2.29996 to 1.4599	0.9227	5	5	0.4552	5.701	0.6658	0.38	ns
Model vs. PAMK500	24.68	24.68	0	-1.5499 to 1.5499	0.7641	5	5	0	6.565	>0.9999	0.00	ns
Model vs. ASP	24.68	24.22	0.46	-0.6999 to 1.6199	0.5795	5	5	0.7938	7.956	0.4503	0.56	ns
D14												
Model vs. Control	24.46	25.54	-1.08	-2.2243 to 0.0643	0.5685	5	5	1.9	7.975	0.0941	1.35	ns
Model vs. Pre-PAMK125	24.46	25.34	-0.88	-2.8427 to 1.0827	0.9691	5	5	0.908	5.496	0.4019	0.77	ns
Model vs. PAMK125	24.46	24.14	0.32	-0.6632 to 1.3032	0.485	5	5	0.6598	7.349	0.5295	0.49	ns
Model vs. Pre-PAMK250	24.46	25.88	-1.42	-2.6630 to -0.1770	0.6132	5	5	2.316	7.726	0.0504	1.67	ns
Model vs. PAMK250	24.46	25.06	-0.6	-1.6837 to 0.4837	0.5378	5	5	1.116	7.976	0.297	0.79	ns
Model vs. Pre-PAMK500	24.46	24.9	-0.44	-2.1630 to 1.2830	0.8483	5	5	0.5187	6.008	0.6225	0.42	ns
Model vs. PAMK500	24.46	24.6	-0.14	-1.4033 to 1.1233	0.629	5	5	0.2226	7.603	0.8297	0.16	ns
Model vs. ASP	24.46	24.36	0.1	-1.0132 to 1.2132	0.5543	5	5	0.1804	8	0.8613	0.13	ns

Fig. S1 e

Group1 vs Group 2	Mean 1	Mean 2	Mean Diff.	95% CI of diff.	SE of diff.	n1	n2	t	DF	Adjusted P Value	Effectsize (Cohen's d)	Summary
Model vs. Control	0.1478	0.2371	-0.08926	-0.1489 to -	0.01545	5	5	5.779	6.266	0.0074	4.62	**
Model vs. Pre-PAMK125	0.1478	0.1734	-0.02552	-0.0780 to 0.0270	0.0143	5	5	1.784	6.666	0.5261	1.38	ns
Model vs. PAMK125	0.1478	0.1604	-0.01256	-0.1836 to 0.1585	0.03701	5	5	0.3395	4.344	0.9999	0.33	ns
Model vs. Pre-PAMK250	0.1478	0.1701	-0.02226	-0.1907 to 0.1462	0.03645	5	5	0.6108	4.355	0.9932	0.59	ns
Model vs. PAMK250	0.1478	0.1483	-0.0004654	-0.1205 to 0.1196	0.02896	5	5	0.01607	4.575	>0.9999	0.02	ns
Model vs. Pre-PAMK500	0.1478	0.1393	0.008533	-0.0762 to 0.0933	0.02045	5	5	0.4172	5.221	0.9995	0.37	ns
Model vs. PAMK500	0.1478	0.1918	-0.04396	-0.0930 to 0.0051	0.01338	5	5	3.285	7.044	0.0814	2.48	ns
Model vs. ASP	0.1478	0.189	-0.04111	-0.0995 to 0.0173	0.01514	5	5	2.715	6.365	0.1877	2.15	ns

Notes: Cohen's d was calculated as d=2t/SQRT(df) to represent effect size; 95% CI, 95% confidence interval of mean difference; **P < 0.01, *P < 0.05, ns: not significant.

Fig. S1 f

Group1 vs Group 2	Mean 1	Mean 2	Mean Diff.	95% CI of diff.	SE of diff.	n1	n2	t	DF	Adjusted P Value	Effectsize (Cohen's d)	Summary
Control vs. Model	0.3228	0.3968	-0.074	-0.1393 to -0.0087	0.01248	5	5	5.929	5.167	0.0298	5.22	*
Model vs. Pre-PAMK125	0.3968	0.3388	0.05794	-0.01336 to 0.1292	0.0163	5	5	3.554	7.997	0.1393	2.51	ns
Model vs. PAMK125	0.3968	0.3808	0.01594	-0.0726 to 0.1044	0.01938	5	5	0.8221	7.425	>0.9999	0.60	ns
Model vs. Pre-PAMK250	0.3968	0.3286	0.06816	0.0004 to 0.1360	0.0155	5	5	4.398	7.87	0.0486	3.14	*
Model vs. PAMK250	0.3968	0.356	0.04079	-0.02412 to 0.1057	0.01343	5	5	3.037	6.384	0.3006	2.40	ns
Model vs. Pre-PAMK500	0.3968	0.3473	0.04952	-0.03031 to 0.1293	0.01825	5	5	2.713	7.732	0.3852	1.95	ns
Model vs. PAMK500	0.3968	0.3866	0.01015	-0.08069 to 0.1010	0.01989	5	5	0.51	7.278	>0.9999	0.38	ns
Model vs. ASP	0.3968	0.3803	0.01651	-0.09535 to 0.1284	0.02314	5	5	0.7132	6.432	>0.9999	0.56	ns

Fig. 1b

Group1 vs Group 2	Mean 1	Mean 2	Mean Diff.	95% CI of diff.	SE of diff.	N1	N2	t	DF	Individual P Value	Effectsize (Cohen's d)	Summary
D6												
Model vs. Pre-PAMK125	86.49	42.98	43.51	26.3715 to 60.6485	7.866	8	8	5.532	12.24	0.0001	3.16	***
Model vs. Pre-PAMK250	86.49	30.89	55.59	39.5698 to 71.6102	7.081	8	8	7.852	9.374	<0.0001	5.13	***
D8												
Model vs. Pre-PAMK125	91.93	51.93	40.01	12.5902 to 67.4298	12.82	8	8	3.12	13.3	0.0079	1.71	**
Model vs. Pre-PAMK250	91.93	42.14	49.8	19.2269 to 80.3731	14.3	8	8	3.482	14	0.0037	1.86	**
D10												
Model vs. Pre-PAMK125	155	84.53	70.5	7.6610 to 133.3390	29.35	8	8	2.402	13.89	0.0309	1.29	*
Model vs. Pre-PAMK250	155	81.16	73.87	11.4801 to	28.92	8	8	2.554	13.8	0.0231	1.38	*
D12												
Model vs. Pre-PAMK125	241.4	150.2	91.12	-4.9601 to	44.28	8	8	2.058	13.38	0.0597	1.13	ns
Model vs. Pre-PAMK250	241.4	132.1	109.2	29.3202 to	37.53	8	8	2.911	13.88	0.0115	1.56	*
D14												
Model vs. Pre-PAMK125	356.3	251.4	104.9	-70.5306 to	77.58	8	8	1.352	9.392	0.208	0.88	ns
Model vs. Pre-PAMK250	356.3	172.2	184.1	85.6565 to	45.83	8	8	4.017	13.72	0.0013	2.17	**

Fig. 1d Dunnett's multiple comparisons test

Group1 vs Group 2	Mean 1	Mean 2	Mean Diff.	95.00% CI of diff.	SE of diff.	n1	n2	q	DF	Adjusted P Value	Effectsize (Cohen's d)	Summary
Model vs. Pre-PAMK125	0.3473	0.2157	0.1315	0.006113 to 0.2570	0.05291	8	8	2.486	21	0.0393	1.08	*
Model vs. Pre-PAMK250	0.3473	0.172	0.1753	0.04988 to 0.3007	0.05291	8	8	3.313	21	0.0063	1.45	**

Fig. 1f

Group1 vs Group 2	Mean 1	Mean 2	Mean Diff.	95.00% CI of diff.	SE of diff.	N1	N2	t	DF	Individual P Value	Effectsize (Cohen's d)	Summary
D-14												
Model vs. Control	19	19.06	-0.0625	-1.134 to 1.009	0.3955	8	8	0.158	11.22	0.8772	0.09	ns
Model vs. Pre-PAMK125	19	19.06	-0.0625	-0.8015 to 0.6765	0.2809	8	8	0.2225	14	0.8271	0.12	ns
Model vs. Pre-PAMK250	19	19.03	-0.025	-0.7335 to 0.6835	0.2691	8	8	0.0929	13.9	0.9273	0.05	ns
D-12												
Model vs. Control	20.25	20.21	0.0375	-1.018 to 1.093	0.4006	8	8	0.0936	13.79	0.9268	0.05	ns
Model vs. Pre-PAMK125	20.25	20.56	-0.3125	-1.314 to 0.6893	0.3808	8	8	0.8207	13.99	0.4256	0.44	ns
Model vs. Pre-PAMK250	20.25	20.15	0.1	-0.8731 to 1.073	0.3698	8	8	0.2704	13.99	0.7908	0.14	ns
D-10												
Model vs. Control	20.95	20.75	0.2	-1.014 to 1.414	0.461	8	8	0.4339	13.91	0.671	0.23	ns
Model vs. Pre-PAMK125	20.95	21.09	-0.1375	-1.183 to 0.9081	0.3944	8	8	0.3487	13.12	0.7329	0.19	ns
Model vs. Pre-PAMK250	20.95	21.04	-0.0875	-1.119 to 0.9445	0.3882	8	8	0.2254	12.85	0.8252	0.13	ns
D-8												
Model vs. Control	21.15	21.26	-0.1125	-1.326 to 1.101	0.4602	8	8	0.2445	13.68	0.8105	0.13	ns
Model vs. Pre-PAMK125	21.15	21.64	-0.4875	-1.425 to 0.4496	0.3459	8	8	1.41	11.21	0.1858	0.84	ns
Model vs. Pre-PAMK250	21.15	21.65	-0.5	-1.500 to 0.4998	0.377	8	8	1.326	13.11	0.2074	0.73	ns
D-6												
Model vs. Control	21.3	21.56	-0.2625	-1.608 to 1.083	0.5095	8	8	0.5152	13.56	0.6147	0.28	ns
Model vs. Pre-PAMK125	21.3	22.06	-0.7625	-2.010 to 0.4851	0.4644	8	8	1.642	11.89	0.1268	0.95	ns
Model vs. Pre-PAMK250	21.3	21.96	-0.6625	-1.932 to 0.6070	0.4754	8	8	1.393	12.43	0.1879	0.79	ns
D-4												
Model vs. Control	21.93	22.05	-0.125	-1.597 to 1.347	0.5586	8	8	0.2238	13.79	0.8262	0.12	ns
Model vs. Pre-PAMK125	21.93	22.26	-0.3375	-1.632 to 0.9565	0.4738	8	8	0.7123	10.65	0.4916	0.44	ns
Model vs. Pre-PAMK250	21.93	22.14	-0.2125	-1.474 to 1.049	0.4495	8	8	0.4728	9.089	0.6475	0.31	ns
D-2												
Model vs. Control	22.06	22.23	-0.1625	-1.484 to 1.159	0.4985	8	8	0.326	13.1	0.7496	0.18	ns
Model vs. Pre-PAMK125	22.06	22.64	-0.575	-1.823 to 0.6726	0.4621	8	8	1.244	11.48	0.2382	0.73	ns
Model vs. Pre-PAMK250	22.06	22.19	-0.125	-1.351 to 1.101	0.4494	8	8	0.2782	10.72	0.7862	0.17	ns
D0												
Model vs. Control	22.35	22.39	-0.0375	-1.321 to 1.246	0.4875	8	8	0.07692	13.89	0.9398	0.04	ns
Model vs. Pre-PAMK125	22.35	22.74	-0.3875	-1.541 to 0.7661	0.4305	8	8	0.9001	12.11	0.3856	0.52	ns
Model vs. Pre-PAMK250	22.35	22.6	-0.25	-1.405 to 0.9053	0.4314	8	8	0.5796	12.15	0.5728	0.33	ns
D2												
Model vs. Control	22.73	23.05	-0.325	-1.493 to 0.8432	0.4423	8	8	0.7348	13.55	0.475	0.40	ns
Model vs. Pre-PAMK125	22.73	23.3	-0.575	-1.486 to 0.3361	0.3406	8	8	1.688	12.25	0.1166	0.96	ns
Model vs. Pre-PAMK250	22.73	22.64	0.0875	-0.8552 to 1.030	0.3554	8	8	0.2462	13.08	0.8094	0.14	ns
D4												
Model vs. Control	23.36	23.51	-0.15	-1.346 to 1.046	0.4533	8	8	0.3309	13.68	0.7457	0.18	ns
Model vs. Pre-PAMK125	23.36	24.01	-0.65	-1.611 to 0.3109	0.3602	8	8	1.804	12.53	0.0953	1.02	ns
Model vs. Pre-PAMK250	23.36	23.63	-0.2625	-1.218 to 0.6925	0.3574	8	8	0.7344	12.36	0.4764	0.42	ns
D6												
Model vs. Control	23.56	24.09	-0.525	-1.615 to 0.5649	0.4133	8	8	1.27	13.73	0.2251	0.69	ns
Model vs. Pre-PAMK125	23.56	23.93	-0.3625	-1.294 to 0.5695	0.3529	8	8	1.027	13.55	0.3223	0.56	ns
Model vs. Pre-PAMK250	23.56	23.55	0.0125	-0.9318 to 0.9568	0.358	8	8	0.03491	13.7	0.9727	0.02	ns
D8												
Model vs. Control	23.81	24.25	-0.4375	-1.641 to 0.7662	0.4506	8	8	0.971	12.38	0.3501	0.55	ns
Model vs. Pre-PAMK125	23.81	24.23	-0.4125	-1.238 to 0.4125	0.3089	8	8	1.335	12.41	0.2057	0.76	ns
Model vs. Pre-PAMK250	23.81	23.93	-0.1125	-1.020 to 0.7946	0.3444	8	8	0.3266	13.88	0.7488	0.18	ns
D10												
Model vs. Control	24.1	24.28	-0.175	-1.540 to 1.190	0.5147	8	8	0.34	13.12	0.7392	0.19	ns
Model vs. Pre-PAMK125	24.1	24.7	-0.6	-1.635 to 0.4354	0.3896	8	8	1.54	12.89	0.1477	0.86	ns
Model vs. Pre-PAMK250	24.1	24.45	-0.35	-1.349 to 0.6487	0.3718	8	8	0.9414	11.89	0.3652	0.55	ns
D12												
Model vs. Control	23.79	24.45	-0.6625	-1.884 to 0.5588	0.4638	8	8	1.428	13.91	0.1753	0.77	ns
Model vs. Pre-PAMK125	23.79	24.25	-0.4625	-1.493 to 0.5679	0.387	8	8	1.195	12.71	0.2539	0.67	ns
Model vs. Pre-PAMK250	23.79	24.23	-0.4375	-1.441 to 0.5658	0.3738	8	8	1.17	11.96	0.2646	0.68	ns
D14												
Model vs. Control	23.76	24.49	-0.725	-1.904 to 0.4540	0.4482	8	8	1.618	14	0.128	0.86	ns
Model vs. Pre-PAMK125	23.76	24.31	-0.55	-1.565 to 0.4655	0.3796	8	8	1.449	12.25	0.1724	0.83	ns
Model vs. Pre-PAMK250	23.76	24.29	-0.525	-1.513 to 0.4632	0.3652	8	8	1.438	11.3	0.1776	0.86	ns

Notes: Cohen's d was calculated as d=2t/SQRT(df) to represent effect size; 95% CI, 95% confidence interval of mean difference; **P < 0.01, *P < 0.05, ns: not significant.

Fig. 1g

Group1 vs Group 2	Mean 1	Mean 2	Mean Diff.	95.00% CI of diff.	SE of diff.	n1	n2	q	DF	Adjusted P Value	Effectsize (Cohen's d)	Summary
Model vs. Control	0.19	0.2492	-0.05922	-0.09106 to -	0.01282	8	8	4.618	28	0.0002	1.75	***
Model vs. Pre-PAMK125	0.19	0.2042	-0.01426	-0.04610 to	0.01282	8	8	1.112	28	0.5548	0.42	ns
Model vs. Pre-PAMK250	0.19	0.2149	-0.02497	-0.05682 to	0.01282	8	8	1.947	28	0.1494	0.74	ns

Fig. 1h

Group1 vs Group 2	Mean 1	Mean 2	Mean Diff.	95.00% CI of diff.	SE of diff.	n1	n2	q	DF	Adjusted P Value	Effectsize (Cohen's d)	Summary
Model vs. Control	0.3778	0.3319	0.04592	0.01724 to 0.07459	0.01155	8	8	3.976	28	0.0013	1.50	**
Model vs. Pre-PAMK125	0.3778	0.3506	0.02716	-0.001511 to	0.01155	8	8	2.352	28	0.0663	0.89	ns
Model vs. Pre-PAMK250	0.3778	0.3441	0.03366	0.004984 to	0.01155	8	8	2.915	28	0.0186	1.10	*

Notes: Cohen's d was calculated as d=2t/SQRT(df) to represent effect size; 95% CI, 95% confidence interval of mean difference; **P < 0.01, *P < 0.05, ns: not significant.

Fig. 2a Dunnett's multiple comparisons test

Group1 vs Group 2	Mean 1	Mean 2	Mean Diff.	95.00% CI of diff.	SE of diff.	n1	n2	q	DF	Adjusted P Value	Effectsize (Cohen's d)	Summary
Model vs. Control	3.07	4.013	-0.9429	-1.580 to -0.3060	0.254	7	7	3.712	24	0.003	1.52	**
Model vs. Pre-PAMK125	3.07	3.347	-0.2771	-0.9140 to 0.3597	0.254	7	7	1.091	24	0.5701	0.45	ns
Model vs. Pre-PAMK250	3.07	4.747	-1.677	-2.314 to -1.040	0.254	7	7	6.602	24	<0.0001	2.70	***

Fig. 2b-CD4+ Dunn's multiple comparisons test

Group1 vs Group 2	Mean 1	Mean 2	Mean Diff.	95% CI of diff.	SE of diff.	n1	n2	q	DF	Adjusted P Value	Effectsize (Cohen's d)	Summary
Model vs. Control	74.16	77.44	-3.286	-6.104 to -0.4671	1.124	7	7	2.922	24	0.1424	1.19	ns
Model vs. Pre-PAMK125	74.16	71.8	2.357	-0.4615 to 5.176	1.124	7	7	2.096	24	0.581	0.86	ns
Model vs. Pre-PAMK250	74.16	77.3	-3.143	-5.962 to -0.3242	1.124	7	7	2.795	24	0.1424	1.14	ns

Fig. 2b-CD8+

Group1 vs Group 2	Mean 1	Mean 2	Mean Diff.	95% CI of diff.	SE of diff.	n1	n2	q	DF	Adjusted P Value	Effectsize (Cohen's d)	Summary
Model vs. Control	13.46	8.459	4.999	2.674 to 7.324	0.9275	7	7	5.389	24	0.0006	2.20	***
Model vs. Pre-PAMK125	13.46	12.05	1.407	-0.9179 to 3.732	0.9275	7	7	1.517	24	0.8288	0.62	ns
Model vs. Pre-PAMK250	13.46	10.8	2.654	0.3292 to 4.979	0.9275	7	7	2.862	24	0.1369	1.17	ns

Fig. 2c

Group1 vs Group 2	Mean 1	Mean 2	Mean Diff.	95% CI of diff.	SE of diff.	n1	n2	q	DF	Adjusted P Value	Effectsize (Cohen's d)	Summary
Model vs. Control	27.89	39.08	-11.19	-18.54 to -3.836	2.96	8	8	3.779	28	0.0021	1.43	**
Model vs. Pre-PAMK125	27.89	27.86	0.025	-7.326 to 7.376	2.96	8	8	0.00845	28	>0.9999	0.00	ns
Model vs. Pre-PAMK250	27.89	31.56	-3.675	-11.03 to 3.676	2.96	8	8	1.241	28	0.4707	0.47	ns

Fig. 2d

Group1 vs Group 2	Mean 1	Mean 2	Mean Diff.	95% CI of diff.	SE of diff.	n1	n2	q	DF	Adjusted P Value	Effectsize (Cohen's d)	Summary
Model vs. Control	8.076	20.95	-12.87	-23.17 to -2.569	4.109	7	7	3.132	24	0.0123	1.28	*
Model vs. Pre-PAMK125	8.076	21.16	-13.08	-23.38 to -2.781	4.109	7	7	3.184	24	0.0109	1.30	*
Model vs. Pre-PAMK250	8.076	20.43	-12.35	-22.65 to -2.051	4.109	7	7	3.006	24	0.0164	1.23	*

Fig. 2e

Group1 vs Group 2	Mean 1	Mean 2	Mean Diff.	95% CI of diff.	SE of diff.	n1	n2	q	DF	Adjusted P Value	Effectsize (Cohen's d)	Summary
Model vs. Control	7.028	6.107	0.9217	-3.217 to 5.061	1.629	6	6	0.5657	20	0.8957	0.25	ns
Model vs. Pre-PAMK125	7.028	5.138	1.89	-2.249 to 6.029	1.629	6	6	1.16	20	0.5267	0.52	ns
Model vs. Pre-PAMK250	7.028	5.323	1.705	-2.434 to 5.844	1.629	6	6	1.047	20	0.6015	0.47	ns

Fig. 2k spleen

Group1 vs Group 2	Mean 1	Mean 2	Mean Diff.	95% CI of diff.	SE of diff.	n1	n2	q	DF	Adjusted P Value	Effectsize (Cohen's d)	Summary
Model vs. Control	5.296	9.718	-4.421	-5.694 to -3.149	0.5008	6	6	8.828	20	<0.0001	3.95	***
Model vs. Pre-PAMK125	5.296	6.212	-0.9159	-2.188 to 0.3564	0.5008	6	6	1.829	20	0.1938	0.82	ns
Model vs. Pre-PAMK250	5.296	7.765	-2.469	-3.741 to -1.196	0.5008	6	6	4.929	20	0.0002	2.20	***

Fig. 2k tumor

Group1 vs Group 2	Mean 1	Mean 2	Mean Diff.	95% CI of diff.	SE of diff.	n1	n2	q	DF	Adjusted P Value	Effectsize (Cohen's d)	Summary
Model vs. Pre-PAMK125	0.6457	1.034	-0.3879	-0.6537 to -0.1221	0.1247	6	6	3.111	15	0.0133	1.61	*
Model vs. Pre-PAMK250	0.6457	1.002	-0.3561	-0.6219 to -0.0903	0.1247	6	6	2.857	15	0.0221	1.48	*

Fig. 2f Dunn's multiple comparisons test

Group1 vs Group 2	Mean 1	Mean 2	Mean Diff.	95% CI of diff.	SE of diff.	n1	n2	q	DF	Adjusted P Value	Effectsize (Cohen's d)	Summary
Model vs. Pre-PAMK125	10.37	18.56	-8.188	-11.52 to -4.854	1.367	6	6	5.991	15	0.0299	3.09	*
Model vs. Pre-PAMK250	10.37	21.2	-10.84	-14.17 to -7.503	1.367	6	6	7.929	15	0.0013	4.09	**

Fig. 2g-CD4+ Dunn's multiple comparisons test

Group1 vs Group 2	Mean 1	Mean 2	Mean Diff.	95% CI of diff.	SE of diff.	n1	n2	q	DF	Adjusted P Value	Effectsize (Cohen's d)	Summary
Model vs. Pre-PAMK125	8.522	10.42	-1.897	-3.314 to -0.4795	0.5811	6	6	3.264	15	0.0401	1.69	*
Model vs. Pre-PAMK250	8.522	10.58	-2.057	-3.474 to -0.6391	0.5811	6	6	3.539	15	0.0221	1.83	*

Fig. 2g-CD8+

Group1 vs Group 2	Mean 1	Mean 2	Mean Diff.	95% CI of diff.	SE of diff.	n1	n2	q	DF	Adjusted P Value	Effectsize (Cohen's d)	Summary
Model vs. Pre-PAMK125	13.81	10.44	3.365	-0.2991 to 7.030	1.502	6	6	2.24	15	0.0727	1.16	ns
Model vs. Pre-PAMK250	13.81	10.97	2.833	-0.8310 to 6.498	1.502	6	6	1.886	15	0.1375	0.97	ns

Fig. 2h normal distribution unequal variances Dunnett's T3 multiple comparisons test

Group1 vs Group 2	Mean 1	Mean 2	Mean Diff.	95% CI of diff.	SE of diff.	n1	n2	q	DF	Adjusted P Value	Effectsize (Cohen's d)	Summary
Model vs. Pre-PAMK125	8.502	9.095	-0.5924	-3.064 to 1.879	1.013	6	6	0.5847	15	0.8479	0.30	ns
Model vs. Pre-PAMK250	8.502	9.271	-0.7683	-3.240 to 1.703	1.013	6	6	0.7584	15	0.7313	0.39	ns

Fig. 2i non-normal distribution equal variances

Group1 vs Group 2	Mean 1	Mean 2	Mean Diff.	95% CI of diff.	SE of diff.	n1	n2	q	DF	Adjusted P Value	Effectsize (Cohen's d)	Summary
Model vs. Pre-PAMK125	7.175	18.33	-11.16	-21.92 to -0.3974	4.411	6	6	2.529	15	0.2088	1.31	ns
Model vs. Pre-PAMK250	7.175	54.95	-47.78	-58.54 to -37.01	4.411	6	6	10.83	15	0.0004	5.59	***

Fig. 2j

Group1 vs Group 2	Mean 1	Mean 2	Mean Diff.	95% CI of diff.	SE of diff.	n1	n2	q	DF	Adjusted P Value	Effectsize (Cohen's d)	Summary
Model vs. Pre-PAMK125	29.32	33.88	-4.567	-14.76 to 5.622	4.177	6	6	1.093	15	0.4611	0.56	ns
Model vs. Pre-PAMK250	29.32	38.1	-8.783	-18.97 to 1.406	4.177	6	6	2.103	15	0.0936	1.09	ns

Fig. 2l

Group1 vs Group 2	Mean 1	Mean 2	Mean Diff.	95% CI of diff.	SE of diff.	n1	n2	q	DF	Adjusted P Value	Effectsize (Cohen's d)	Summary
Model vs. Control	21.7	42.21	-20.51	-31.97 to -9.054	4.614	8	8	4.445	28	0.0004	1.68	***
Model vs. Pre-PAMK125	21.7	30.06	-8.363	-19.82 to 3.096	4.614	8	8	1.812	28	0.1915	0.68	ns
Model vs. Pre-PAMK250	21.7	38.31	-16.61	-28.07 to -5.154	4.614	8	8	3.6	28	0.0034	1.36	**

Fig. 3d Paired t test normal distribution

Time	Mean of Model	Mean of PrePAMK	Mean Diff.	SE of diff.	n1	n2	t ratio	df	q value	P value	Effectsize (Cohen's d)	Summary
D6	51.93	49.6	2.335	12.08	8	8	0.1933	7	0.87856	0.852191	0.15	ns
D8	83.39	77.84	5.548	18.04	8	8	0.3074	7	0.87856	0.767454	0.23	ns
D10	129.9	125.5	4.38	25.77	8	8	0.1699	7	0.87856	0.869861	0.13	ns
D12	216.2	198.1	18.08	35.56	8	8	0.5085	7	0.87856	0.626749	0.38	ns
D14	321.8	298.9	22.82	36.12	8	8	0.6318	7	0.87856	0.547566	0.48	ns

Fig. 3e Unpaired t test normal distribution

Mean of Model	Mean of Pre-PAMK	Mean Diff. ± SEM	95% CI of diff.	R squared (eta squared)	n1	n2	t	df	P value	Effectsize (Cohen's d)	summary
0.3591	0.3341	-0.0249 ± 0.0503	-0.1328 to 0.0829	0.01727	8	8	0.496	14	0.6276	0.27	ns

Notes: Cohen's d was calculated as d=2t/SQRT(df) to represent effect size; 95% CI, 95% confidence interval of mean difference; ***P < 0.001, **P < 0.01, *P < 0.05, ns: not significant.

Fig. 3g normal distribution — equal variances — Unpaired t test

Mean of Model	Mean of Pre-PAMK	Mean Diff. ± SEM	95% CI	R squared (eta squared)	n1	n2	t	df	P value	Effectsize (Cohen‘s d)	summary
0.4156	0.4053	-0.01032 ± 0.006946	-0.0252 to 0.0046	0.1361	8	8	1.485	14	0.1596	0.79	ns

Fig. 3h normal distribution — equal variances — Unpaired t test

Mean of Model	Mean of Pre-PAMK	Mean Diff. ± SEM	95% CI	R squared (eta squared)	n1	n2	t	df	P value	Effectsize (Cohen‘s d)	summary
4.323	4.488	0.1650 ± 0.5301	-1.016 to 1.346	0.009596	6	6	0.3113	10	0.762	0.20	ns

Fig. 3i CD4 normal distribution — equal variances — Unpaired t test

Mean of Model	Mean of Pre-PAMK	Mean Diff. ± SEM	95% CI	R squared (eta squared)	n1	n2	t	df	P value	Effectsize (Cohen‘s d)	summary
83.72	84.25	0.5333 ± 0.5435	-0.6778 to 1.744	0.08782	6	6	0.9812	10	0.3496	0.62	ns

Fig. 3i CD8 normal distribution — equal variances — Unpaired t test

Mean of Model	Mean of Pre-PAMK	Mean Diff. ± SEM	95% CI	R squared (eta squared)	n1	n2	t	df	P value	Effectsize (Cohen‘s d)	summary
9.537	8.792	-0.7450 ± 0.3832	-1.599 to 0.1089	0.2743	6	6	1.944	10	0.0805	1.23	ns

Fig. 3j normal distribution — equal variances — Unpaired t test

Mean of Model	Mean of Pre-PAMK	Mean Diff. ± SEM	95% CI	R squared (eta squared)	n1	n2	t	df	P value	Effectsize (Cohen‘s d)	summary
52.73	55.32	2.583 ± 3.595	-5.427 to 10.59	0.04909	6	6	0.7185	10	0.4889	0.45	ns

Fig. 3k normal distribution — equal variances — Unpaired t test

Mean of Model	Mean of Pre-PAMK	Mean Diff. ± SEM	95% CI	R squared (eta squared)	n1	n2	t	df	P value	Effectsize (Cohen‘s d)	summary
8.597	8.732	0.1350 ± 1.339	-2.849 to 3.119	0.001015	6	6	0.1008	10	0.9217	0.06	ns

Fig. 3p normal distribution — equal variances — Unpaired t test

Mean of Model	Mean of Pre-PAMK	Mean Diff. ± SEM	95% CI	R squared (eta squared)	n1	n2	t	df	P value	Effectsize (Cohen‘s d)	summary
8.811	9.631	0.8201 ± 0.3910	-0.05105 to 1.691	0.3055	6	6	2.098	10	0.0623	1.33	ns

Fig. 3p tumor normal distribution — unequal variances — Unpaired t test with Welch's correction

Mean of Model	Mean of Pre-PAMK	Mean Diff. ± SEM	95% CI	R squared (eta squared)	n1	n2	t	df	P value	Effectsize (Cohen‘s d)	summary
1.277	1.249	-0.02760 ± 0.1757	-0.4541 to 0.3989	0.003964	6	6	0.1571	6.203	0.8802	0.13	ns

Fig. 3l non-normal distribution — equal variances — Mann Whitney test

Mean of Model	Mean of Pre-PAMK	Diff.: Actual	Diff.: Hodges-Lehmann	95.89% CI of diff.	n1	n2	Sum of ranks in column A,B	Mann-Whitney U	P value	summary
12.24	12.6	0.3639	0.7747	-1.515 to 5.503	6	6	33 , 45	12	0.3939	ns

Fig. 3m CD4 normal distribution — equal variances — Unpaired t test

Mean of Model	Mean of Pre-PAMK	Mean Diff. ± SEM	95% CI	R squared (eta squared)	n1	n2	t	df	P value	Effectsize (Cohen‘s d)	summary
10.73	11.25	0.5225 ± 1.367	-2.523 to 3.568	0.0144	6	6	0.3822	10	0.7103	0.24	ns

Fig. 3m CD8 normal distribution — equal variances — Unpaired t test

Mean of Model	Mean of Pre-PAMK	Mean Diff. ± SEM	95% CI	R squared (eta	n1	n2	t	df	P value	Effectsize (Cohen‘s	summary
8.766	9.028	0.2619 ± 1.060	-2.100 to 2.623	0.006069	6	6	0.2471	10	0.8098	0.16	ns

Fig. 3n non-normal distribution — equal variances — Mann Whitney test

Mean of Model	Mean of Pre-PAMK	Diff.: Actual	Diff.: Hodges-Lehmann	95.89% CI of diff.	n1	n2	Sum of ranks in column A,B	Mann-Whitney U	P value	summary
8.689	9.958	1.269	0.9259	-1.884 to 2.100	6	6	33 , 45	12	0.3939	ns

Fig. 3o normal distribution — equal variances — Unpaired t test

Mean of Model	Mean of Pre-PAMK	Mean Diff. ± SEM	95% CI	R squared (eta	n1	n2	t	df	P value	Effectsize (Cohen‘s	summary
10.78	11.15	0.5225 ± 1.367	-2.523 to 3.568	0.007676	6	6	0.2718	10	0.7866	0.17	ns

Fig. 3q normal distribution — equal variances — Unpaired t test

Mean of Model	Mean of Pre-PAMK	Mean Diff. ± SEM	95% CI	R squared (eta	n1	n2	t	df	P value	Effectsize (Cohen‘s	summary
18.61	20.61	0.5225 ± 1.367	-2.523 to 3.568	0.04098	8	8	0.7735	14	0.4521	0.41	ns

Fig. 4b normal distribution — False Discovery Rate (FDR) — Paired t test

Time	Mean of FMT-Model	Mean of FMT-PrePAMK	Diff.	SE of diff.	n1	n2	t ratio	df	q value	P value	Effectsize (Cohen‘s d)	Summary
D6	56.99	34.48	22.51	17.04	8	8	1.322	7	0.263499	0.227864	1.00	ns
D8	96.84	70.87	25.97	21.24	8	8	1.223	7	0.263499	0.26089	0.92	ns
D10	161.3	106.5	54.83	32.53	8	8	1.686	7	0.22844	0.135707	1.27	ns
D12	285.6	164.8	120.7	42.4	8	8	2.847	7	0.099198	0.024777	2.15	*
D14	357.5	221.6	135.9	53.74	8	8	2.529	7	0.099198	0.039286	1.91	*

Fig. 4d normal distribution — equal variances — Unpaired t test

Mean of Model	Mean of Pre-PAMK	Mean Diff. ± SEM	95% CI	R squared (eta	n1	n2	t	df	P value	Effectsize (Cohen‘s	summary
0.3111	0.2084	-0.1027 ± 0.04921	-0.2083 to 0.002805	0.2374	8	8	2.088	14	0.0556	1.12	ns

Fig. 4f normal distribution — equal variances — Unpaired t test

Mean of Model	Mean of Pre-PAMK	Mean Diff. ± SEM	95% CI	R squared (eta	n1	n2	t	df	P value	Effectsize (Cohen‘s	summary
0.3553	0.3315	-0.02384 ± 0.008295	-0.04163 to -0.006046	0.371	8	8	2.874	14	0.0123	1.54	*

Fig. 4g normal distribution — equal variances — Unpaired t test

Mean of Model	Mean of Pre-PAMK	Mean Diff. ± SEM	95% CI	R squared (eta	n1	n2	t	df	P value	Effectsize (Cohen‘s	summary
3.572	4.973	1.402 ± 0.5663	0.1399 to 2.663	0.3799	6	6	2.475	10	0.328	1.57	*

Fig. 4h CD4+ normal distribution — equal variances — Unpaired t test

Mean of Model	Mean of Pre-PAMK	Mean Diff. ± SEM	95% CI	R squared (eta	n1	n2	t	df	P value	Effectsize (Cohen‘s	summary
84.68	86.53	1.850 ± 0.5126	0.7869 to 2.913	0.3719	6	6	3.609	10	0.0016	2.28	**

Fig. 4h CD8+ normal distribution — equal variances — Unpaired t test

Mean of Model	Mean of Pre-PAMK	Mean Diff. ± SEM	95% CI	R squared (eta	n1	n2	t	df	P value	Effectsize (Cohen‘s	summary
8.452	7.248	-1.203 ± 0.6627	-2.680 to 0.2731	0.248	6	6	1.816	10	0.0994	1.15	ns

Fig. 4i normal distribution — equal variances — Unpaired t test

Mean of Model	Mean of Pre-PAMK	Mean Diff. ± SEM	95% CI	R squared (eta	n1	n2	t	df	P value	Effectsize (Cohen‘s	summary
6.687	11.44	4.753 ± 1.153	2.184 to 7.323	0.6295	6	6	4.122	10	0.0021	2.61	**

Fig. 4m spleen non-normal distribution — equal variances — Mann Whitney test

Mean of Model	Mean of Pre-PAMK	Diff.: Actual	Diff.: Hodges-Lehmann	95.89% CI of diff.	n1	n2	Sum of ranks in column A,B	Mann-Whitney U	P value	summary
10.56	11.1	0.5452	1.025	-0.1840 to 4.517	6	6	31 , 47	10	0.2403	ns

Fig. 4m normal distribution — equal variances — Unpaired t test

Mean of Model	Mean of Pre-PAMK	Mean Diff. ± SEM	95% CI	R squared (eta	n1	n2	t	df	P value	Effectsize (Cohen‘s	summary
1.181	1.437	0.2558 ± 0.1202	-0.01192 to 0.5236	0.3119	6	6	2.129	10	0.0591	1.35	ns

Fig. 4j normal distribution — equal variances — Unpaired t test

Mean of Model	Mean of Pre-PAMK	Mean Diff. ± SEM	95% CI	R squared (eta	n1	n2	t	df	P value	Effectsize (Cohen‘s	summary
9.769	16.92	7.147 ± 1.091	4.717 to 9.578	0.8111	6	6	6.553	10	<0.0001	4.14	***

Fig. 4K CD4+ normal distribution — equal variances — Unpaired t test

Mean of Model	Mean of Pre-PAMK	Mean Diff. ± SEM	95% CI	R squared (eta	n1	n2	t	df	P value	Effectsize (Cohen‘s	summary
10.79	12.53	1.744 ± 0.6791	0.2306 to 3.257	0.3973	6	6	2.568	10	0.028	1.62	*

Fig. 4k CD8+ normal distribution — unequal variances — Unpaired t test with Welch's correction

Mean of Model	Mean of Pre-PAMK	Mean Diff. ± SEM	95% CI	R squared (eta	n1	n2	corrected t	corrected df	P value	Effectsize (Cohen‘s	summary
9.141	8.96	-0.1811 ± 0.7124	-1.942 to 1.580	0.01109	6	6	0.2542	5.76	0.8082	0.21	ns

Fig. 4l normal distribution — equal variances — Unpaired t test

Mean of Model	Mean of Pre-PAMK	Mean Diff. ± SEM	95% CI	R squared (eta	n1	n2	t	df	P value	Effectsize (Cohen‘s	summary
8.03	11.97	3.940 ± 1.669	0.2214 to 7.659	0.3579	6	6	2.361	10	0.0399	1.49	*

Fig. 4n normal distribution — equal variances — Unpaired t test

Mean of Model	Mean of Pre-PAMK	Mean Diff. ± SEM	95% CI	R squared (eta	n1	n2	t	df	P value	Effectsize (Cohen‘s	summary
20.25	27.06	6.813 ± 2.540	1.365 to 12.26	0.3395	8	8	2.682	14	0.0179	1.43	*

Fig. 7h *Mmp9* normal distribution — unequal variances — Unpaired t test with Welch's correction

Mean of Model	Mean of Pre-PAMK	Mean Diff. ± SEM	95% CI	R squared (eta	n1	n2	corrected t	corrected df	P value	Effectsize (Cohen‘s	summary
2.479	0.6209	-1.858 ± 0.6014	-4.427 to 0.7111	0.8256	3	3	3.089	2.015	0.0899	4.35	ns

Ccl19 normal distribution — equal variances — Unpaired t test

Mean of Model	Mean of Pre-PAMK	Mean Diff. ± SEM	95% CI	R squared (eta	n1	n2	t	df	P value	Effectsize (Cohen‘s	summary
0.7388	0.961	0.2222 ± 0.02781	0.1450 to 0.2994	0.941	3	3	7.99	4	0.0013	7.99	**

mt-Atp6 normal distribution — equal variances — Unpaired t test

Mean of Model	Mean of Pre-PAMK	Mean Diff. ± SEM	95% CI	R squared (eta	n1	n2	t	df	P value	Effectsize (Cohen‘s	summary
0.6029	1.358	0.7546 ± 0.1578	0.3165 to 1.193	0.8511	3	3	4.782	4	0.0088	4.78	**

Cxcl9 normal distribution — unequal variances — Unpaired t test with Welch's correction

Mean of Model	Mean of Pre-PAMK	Mean Diff. ± SEM	95% CI	R squared (eta	n1	n2	corrected t	corrected df	P value	Effectsize (Cohen‘s	summary
0.4024	1.301	0.8984 ± 0.1880	0.1102 to 1.687	0.9174	3	3	4.778	2.056	0.0389	6.66	*

mt-Co3 normal distribution — equal variances — Unpaired t test

Mean of Model	Mean of Pre-PAMK	Mean Diff. ± SEM	95% CI	R squared (eta	n1	n2	t	df	P value	Effectsize (Cohen‘s	summary
0.5135	1.79	1.277 ± 0.1441	0.8768 to 1.677	0.9515	3	3	8.861	4	0.0009	8.86	***

Per2 normal distribution — unequal variances — Unpaired t test with Welch's correction

Mean of Model	Mean of Pre-PAMK	Mean Diff. ± SEM	95% CI	R squared (eta	n1	n2	corrected t	corrected df	P value	Effectsize (Cohen‘s	summary
0.1837	1.387	1.203 ± 0.2913	-0.03497 to 2.441	0.8939	3	3	4.131	2.026	0.0527	5.80	ns

Notes: Cohen's d was calculated as d=2t/SQRT(df) to represent effect size; 95% CI, 95% confidence interval of mean difference; ***P < 0.001, **P < 0.01, *P < 0.05, ns: not significant.

Fig. 5g **group-specific enriched and differential taxa of gut microbiota （Comparision between Model and Control group）**

Group	Taxonomy	Abundace	LDA_score	P-value
Control	k__Bacteria;p__Bacteroidota;c__Bacteroidia;o__Bacteroidales;f__Rikenellaceae;g__Alistipes	5.6921	5.3592	0.0008
Control	k__Bacteria;p__Bacteroidota;c__Bacteroidia;o__Bacteroidales;f__Rikenellaceae	5.5640	5.2214	0.0008
Control	k__Bacteria;p__Bacteroidota;c__Bacteroidia;o__Bacteroidales;f__Bacteroidaceae; g__Bacteroides; s__Bacteroides_acidifaciens	5.7153	5.1829	0.0033
Control	k__Bacteria;p__Firmicutes;c__Bacilli;o__Lactobacillales;f__Lactobacillaceae; g__Ligilactobacillus;s__Lactobacillus_murinus	5.2294	4.6353	0.0087
Control	k__Bacteria;p__Bacteroidota;c__Bacteroidia;o__Bacteroidales;f__Bacteroidaceae	5.1329	4.4573	0.0209
Control	k__Bacteria;p__Firmicutes;c__Bacilli;o__Lactobacillales	4.8607	4.4275	0.0046
Control	k__Bacteria;p__Firmicutes;c__Bacilli;o__Lactobacillales;f__Lactobacillaceae	4.8440	4.3438	0.0023
Control	k__Bacteria;p__Bacteroidota;c__Bacteroidia;o__Bacteroidales;f__Tannerellaceae; g__Parabacteroides	4.7179	4.2102	0.0063
Control	k__Bacteria;p__Bacteroidota;c__Bacteroidia;o__Bacteroidales;f__Tannerellaceae	4.5813	4.1827	0.0016
Control	k__Bacteria;p__Firmicutes;c__Clostridia;o__unidentified_Clostridia;f__Oscillospiraceae	4.7135	4.0288	0.0157
Control	k__Bacteria;p__unidentified_Bacteria;c__Campylobacterota;o__Campylobacterales; f__Helicobacteraceae;g__Helicobacter; s__Helicobacter_typhlonius	4.2694	3.9295	0.0008
Model	k__Bacteria;p__Bacteroidota;c__Bacteroidia;o__Bacteroidales;f__Muribaculaceae	5.6022	5.1734	0.0008
Model	k__Bacteria;p__Bacteroidota;c__Bacteroidia;o__Bacteroidales;f__Prevotellaceae; g__Alloprevotella	5.3115	4.9900	0.0008
Model	k__Bacteria;p__Bacteroidota;c__Bacteroidia;o__Bacteroidales;f__Prevotellaceae	5.1234	4.8004	0.0008
Model	k__Bacteria;p__Bacteroidota;c__Bacteroidia;o__Bacteroidales;f__Bacteroidaceae; g__Bacteroides;s__Bacteroides_sartorii	5.0516	4.6597	0.0008
Model	k__Bacteria;p__Bacteroidota;c__Bacteroidia;o__Bacteroidales;f__Muribaculaceae; g__Muribaculum; s__Muribaculum_intestinale	4.8017	4.4753	0.0008
Model	k__Bacteria;p__Bacteroidota;c__Bacteroidia;o__Bacteroidales;f__Marinifilaceae;g__Odoribacter	4.7317	4.3589	0.0008
Model	k__Bacteria;p__unidentified_Bacteria;c__Campylobacterota;o__Campylobacterales;f__Helicobacteraceae;g__Helicobacter	4.7583	4.2553	0.0063
Model	k__Bacteria;p__Firmicutes;c__Clostridia;o__unidentified_Clostridia;f__Lachnospiraceae;g__unidentified_Lachnospiraceae; s__Clostridiales_bacterium_CIEAF_020	4.5952	4.2072	0.0011
Model	k__Bacteria;p__Firmicutes;c__Clostridia;o__unidentified_Clostridia;f__Lachnospiraceae;g__unidentified_Lachnospiraceae	4.5364	4.1347	0.0023
Model	k__Bacteria;p__Bacteroidota;c__Bacteroidia;o__Bacteroidales;f__Muribaculaceae;g__Muribaculum	4.4101	4.1124	0.0008
Model	k__Bacteria;p__Proteobacteria;c__Gammaproteobacteria;o__Enterobacterales;f__Enterobacteriaceae; g__unidentified_Enterobacteriaceae;s__Escherichia_coli	4.3171	4.0651	0.0082
Model	k__Bacteria;p__Firmicutes;c__Clostridia;o__unidentified_Clostridia;f__Peptostreptococcaceae; g__Romboutsia	4.3832	3.9959	0.0033
Model	k__Bacteria;p__Firmicutes;c__Clostridia;o__Clostridiales;f__Clostridiaceae; g__unidentified_Clostridiaceae;s__metagenome	4.2964	3.9934	0.0199
Model	k__Bacteria;p__Firmicutes;c__Clostridia;o__unidentified_Clostridia;f__Lachnospiraceae;g__Blautia	4.3402	3.9579	0.0063
Model	k__Bacteria;p__Firmicutes;c__Bacilli;o__Mycoplasmatales;f__Mycoplasmataceae;g__Mycoplasma	4.2150	3.9260	0.0006

Fig. 5h **group-specific enriched and differential taxa of gut microbiota （Comparision between Pre-PAMK and Model group）**

Group	Taxonomy	Abundace	LDA_score	P-value
Model	k__Bacteria;p__Bacteroidota;c__Bacteroidia;o__Bacteroidales;f__Prevotellaceae	5.1234	4.6362	0.0117
Model	k__Bacteria;p__Bacteroidota;c__Bacteroidia;o__Bacteroidales;f__Prevotellaceae;g__Alloprevotella	5.3115	4.9033	0.0016
Model	k__Bacteria;p__Firmicutes;c__Bacilli;o__Lactobacillales;f__Lactobacillaceae;g__Limosilactobacillus;s__Lactobacillus_reuteri	4.3344	3.9211	0.0046
Model	k__Bacteria;p__Firmicutes;c__Clostridia;o__Clostridiales;f__Clostridiaceae;g__unidentified_Clostridiaceae;s__metagenome	4.2964	4.0422	0.0006
Model	k__Bacteria;p__Firmicutes;c__Clostridia;o__unidentified_Clostridia;f__Lachnospiraceae;g__Blautia	4.3402	3.9915	0.0008
Model	k__Bacteria;p__Firmicutes;c__Clostridia;o__unidentified_Clostridia;f__Peptostreptococcaceae;g__Romboutsia	4.3832	3.9059	0.0117
Pre-PAMK	k__Bacteria;p__Bacteroidota;c__Bacteroidia;o__Bacteroidales;f__Muribaculaceae	5.7140	4.8212	0.0357
Pre-PAMK	k__Bacteria;p__Bacteroidota;c__Bacteroidia;o__Bacteroidales;f__Rikenellaceae;g__Alistipes	4.7021	4.1147	0.0357

Supplementary Table 1 Group-specific enriched and differential taxa of gut microbiota （original data, reads)

Taxonomy	C1	C2	C3	C4	C5	C6	C7	C8	A1	A2	A3	A4	A5	A6	A7	A8	B1	B2	B3	B4	B5	B6	B7	B8
k__Bacteria;p__Bacteroidota;c__Bacteroidia;o__Bacteroidales;f__Bacteroidaceae;g__Bacteroides;s__Bacteroides_sartorii;	92	379	266	178	241	92	178	223	740	858	739	3645	312	1085	556	362	530	190	252	2571	884	2091	1337	464
k__Bacteria;p__Proteobacteria;c__Gammaproteobacteria;o__Enterobacterales;f__Enterobacteriaceae;g__unidentified_Enterobacteriaceae;s__Escherichia_coli;	12	11	175	0	2	0	0	0	62	39	35	10	1	7	8	1	18	117	4	30	449	6	15	676
k__Bacteria;p__unidentified_Bacteria;c__Campylobacterota;o__Campylobacterales;f__Helicobacteraceae;g__Helicobacter;s__Helicobacter_typhlonius;	168	80	325	75	168	485	206	85	0	0	0	0	0	0	0	0	0	0	0	5	19	29	30	14
k__Bacteria;p__Firmicutes;c__Bacilli;o__Lactobacillales;f__Lactobacillaceae;g__Ligilactobacillus;s__Lactobacillus_murinus;	10647	2297	2244	989	1592	1062	1111	853	651	1023	223	314	312	420	1116	1243	646	1192	294	541	324	748	426	501
k__Bacteria;p__Firmicutes;c__Bacilli;o__Lactobacillales;f__Lactobacillaceae;g__Limosilactobacillus;s__Lactobacillus_reuteri;	518	402	154	415	549	161	943	430	25	21	46	126	19	29	68	87	158	31	66	189	235	347	225	232
k__Bacteria;p__Firmicutes;c__Clostridia;o__unidentified_Clostridia;f__Lachnospiraceae;g__unidentified_Lachnospiraceae;s__Clostridiales_bacterium_CIEAF_020;	1	6	17	8	5	26	99	80	905	70	53	37	1017	49	62	45	120	58	514	191	560	502	536	154
k__Bacteria;p__Firmicutes;c__Clostridia;o__unidentified_Clostridia;f__Peptostreptococcaceae;g__Romboutsia;	42	1	306	0	4	0	2	8	26	70	30	8	18	7	300	30	2315	305	218	110	122	95	117	657
k__Bacteria;p__Firmicutes;c__Bacilli;o__Mycoplasmatales;f__Mycoplasmataceae;g__Mycoplasma;	0	0	0	0	0	3	1	0	109	87	60	14	12	74	59	146	103	48	27	198	625	806	554	195
k__Bacteria;p__Firmicutes;c__Clostridia;o__Clostridiales;f__Clostridiaceae;g__unidentified_Clostridiaceae;s__metagenome;	242	6	233	0	0	6	0	0	0	0	2	0	0	0	0	4	238	29	56	49	53	29	174	677
k__Bacteria;p__Firmicutes;c__Clostridia;o__unidentified_Clostridia;f__Lachnospiraceae;g__Blautia	484	33	439	2	25	56	48	22	50	7	19	6	32	53	58	38	512	745	229	99	470	176	355	931
k__Bacteria;p__Bacteroidota;c__Bacteroidia;o__Bacteroidales;f__Prevotellaceae;g__Alloprevotella	149	210	343	161	279	820	397	212	118	269	1036	713	926	1538	380	1317	3383	8314	5224	5365	1403	2175	3298	4297
k__Bacteria;p__Bacteroidota;c__Bacteroidia;o__Bacteroidales;f__Muribaculaceae;g__Muribaculum	0	6	0	4	2	6	7	14	204	478	378	394	214	800	346	809	637	330	788	328	563	585	393	476
k__Bacteria;p__Firmicutes;c__Clostridia;o__unidentified_Clostridia;f__Lachnospiraceae;g__unidentified_Lachnospiraceae	281	347	328	70	252	201	352	399	1526	135	163	154	1323	150	159	157	589	198	607	395	1046	1058	888	644
k__Bacteria;p__unidentified_Bacteria;c__Campylobacterota;o__Campylobacterales;f__Helicobacteraceae;g__Helicobacter	337	560	517	534	727	1349	395	829	4037	958	592	1432	1746	1092	759	1259	1789	1049	1013	294	905	1175	2349	472
k__Bacteria;p__Bacteroidota;c__Bacteroidia;o__Bacteroidales;f__Marinifilaceae;g__Odoribacter	141	421	314	462	478	464	430	564	1815	1085	1670	1045	1899	780	1208	1551	570	752	1009	435	2080	2188	1005	361
k__Bacteria;p__Bacteroidota;c__Bacteroidia;o__Bacteroidales;f__Tannerellaceae;g__Parabacteroides	2074	2434	2265	2502	1338	2220	1114	1330	171	291	458	351	297	621	422	216	258	201	1330	286	190	314	366	335
k__Bacteria;p__Bacteroidota;c__Bacteroidia;o__Bacteroidales;f__Tannerellaceae	2074	2434	2265	2502	1338	2220	1114	1330	171	291	458	351	297	621	422	216	258	201	1330	286	190	314	366	335
k__Bacteria;p__Bacteroidota;c__Bacteroidia;o__Bacteroidales;f__Bacteroidaceae;g__Bacteroides;s__Bacteroides_acidifaciens	10032	7884	10136	4468	5907	4834	3558	3422	2410	1800	5168	6034	2115	4457	1948	2506	2078	405	3333	6507	968	1571	1807	1148
k__Bacteria;p__Firmicutes;c__Bacilli;o__Lactobacillales	12043	3312	2547	1802	2836	1600	2563	1812	717	1220	536	810	358	609	1344	1475	1050	1311	458	1913	1138	1795	1199	1508
k__Bacteria;p__Firmicutes;c__Bacilli;o__Lactobacillales;f__Lactobacillaceae	11501	3189	2496	1697	2642	1568	2548	1748	704	1217	534	802	358	606	1333	1467	1031	1295	449	1082	1004	1729	1025	1162
k__Bacteria;p__Bacteroidota;c__Bacteroidia;o__Bacteroidales;f__Bacteroidaceae	10798	8636	10503	4764	6285	5138	3869	3886	3391	2905	6305	10023	2586	6883	6308	3132	3559	727	3873	9713	2494	4423	3754	2474
k__Bacteria;p__Bacteroidota;c__Bacteroidia;o__Bacteroidales;f__Rikenellaceae	7386	15593	11393	29271	21809	17717	20303	24767	1149	1388	2645	888	1422	1147	2219	1321	809	666	2325	1093	1740	2236	1254	1415
k__Bacteria;p__Bacteroidota;c__Bacteroidia;o__Bacteroidales;f__Rikenellaceae;g__Alistipes	6783	15219	11126	28935	21534	17417	19974	24271	476	778	2178	629	712	732	852	431	350	387	790	485	499	893	511	722
k__Bacteria;p__Firmicutes;c__Clostridia;o__unidentified_Clostridia;f__Oscillospiraceae	1708	1868	3623	1245	2912	2784	3604	2922	2873	691	1365	978	2946	1301	1821	1641	1545	1555	1552	1198	1665	2072	1327	1633
k__Bacteria;p__Bacteroidota;c__Bacteroidia;o__Bacteroidales;f__Prevotellaceae	151	270	350	164	291	864	398	218	457	567	2683	2167	6238	3474	1291	5610	4440	10967	6799	8682	4396	5537	5556	7498
k__Bacteria;p__Bacteroidota;c__Bacteroidia;o__Bacteroidales;f__Muribaculaceae	1588	10257	2394	6787	2725	8027	2213	4518	16791	36091	28005	29954	16865	29937	29568	27096	22582	19578	21378	22287	16593	16471	23481	20782

Note: A1-A8: Samples in Pre-PAMK group; B1-B8: Sample in Model group; C1-C8: Sample in Control group;

Fig. 6 **Intergroup representitive differential metabolites**

Comparision		Palmitoleic acid	Dodecanoic acid	α-Linolenic acid	FFA(18:1)	11β-Hydroxy-testosterone	Testosterone glucuronide	Episterone	17α-Estradiol	Estradiol
Model vs Control	VIP	1.1882	1.2807	1.0844	-	-	1.4181	1.2708	-	1.3053
	P-value	0.0041	0.0019	0.0112	-	-	0.0001	0.0035	-	0.0000
	Log2FC	1.6187	2.3502	0.8690	-	-	5.6288	2.3296	-	-1.5116
	Type （in Model）	up	up	up	-	-	up	up	-	down
Pre-PAMK vs Model	VIP	-	2.2203	1.7782	1.5881	2.2406	1.9593	1.8182	1.8743	1.6039
	P-value	-	0.0023	0.0140	0.0247	0.0063	0.0005	0.0296	0.0223	0.0280
	Log2FC	-	-2.0602	-0.8959	-0.7123	-3.7062	-1.9490	-1.0599	2.7113	0.8902
	Type （in Pre-PAMK）	-	down	down	down	down	down	down	up	up

Supplementary Table 2 Intergroup representitive differential metabolites (original data)

Mode	C18_positive	C18_positive	C18_negative	C18_negative	C18_negative	C18_positive	C18_negative	C18_positive	C18_positive
Index	MW0056738	MW0054288	MW0140011	MEDP0904	MW0012082	MW0063777	MW0161573	MEDP1620	MW0012684
Compounds	Palmitoleic acid	Dodecanoic acid	alpha-Linolenic acid	FFA(18:1)	11beta-Hydroxytestosterone	Testosterone glucuronide	Episterone	17alpha-Estradiol	Estradiol
Class I	FA	FA	FA	FA	Aldehyde,Ketones,Esters	Hormones and hormone related compounds	Aldehyde,Ketones,Esters	Hormones and hormone related compounds	Hormones and hormone related compounds
Class II	FFA	FFA	FFA	FFA	Ketones	Hormones and hormone related compounds	Ketones	Hormones and hormone related compounds	Hormones and hormone related compounds
Formula	C16H30O2	C12H24O2	C18H30O2	C18H34O2	C19H28O3	C25H36O8	C28H44O	C18H24O2	C18H24O2
Q1 (Da)	2.19E+02	4.39E+02	2.77E+02	5.64E+02	3.03E+02	4.87E+02	4.31E+02	2.37E+02	3.19E+02
Molecular weight (Da)	2.54E+02	2.00E+02	2.78E+02	2.82E+02	3.04E+02	4.64E+02	3.96E+02	2.72E+02	2.72E+02
RT (min)	9.00E+00	1.05E+01	9.66E+00	1.20E+01	9.60E+00	6.37E+00	9.60E+00	2.06E+00	6.84E+00
Adduct	M+H-2H2O	2M+K	M-H	2M-H	M-H	M+Na	M+Cl	M+H-2H2O	M+HCOO+2H
Mass error	1.30E+00	4.56E+00	2.45E+00	2.44E+00	2.35E+00	1.09E+01	1.25E+01	8.97E+00	8.00E-01
Level	3	2	3	1	3	2	3	2	3
score	9.94E-01	7.02E-01	8.29E-01	9.54E-01	6.21E-01	6.91E-01	7.60E-01	6.60E-01	6.11E-01
LC mode	rp	rp	rp	rp	rp	rp	rp	rp	rp
CAS	373-49-9	143-07-7	463-40-1	112-80-1	-	1180-25-2	-	57-91-0	50-28-2
PubChem	4.46E+05	3.89E+03	5.28E+06	4.46E+05	1.15E+05	1.08E+05	1.48E+07	6.86E+04	5.76E+03
HMDB	HMDB0003229	HMDB0000638	HMDB0001388	HMDB0000207	HMDB0060339	HMDB0003193	-	HMDB0000429	HMDB0000151
Metlin	1.88E+02	5.61E+03	6.21E+03	-	-	2.79E+03	-	5.42E+03	2.63E+02
cpd_ID	C08362	C02679	C06427	C00712	C18075	C11134	C22122	C02537	C00951
1A1	3876.6959	10314.3429	16008.6208	132143.3965	246.7367	441.8302	6471.5415	6143.6644	21429.6711
1A2	1413.8234	26753.3658	9172.2062	205549.0392	13.4078	1644.9983	5202.8832	26563.5682	12209.3191
1A3	3075.7542	7554.8957	7054.5848	149360.9612	29.3335	3689.7485	7955.0496	33532.2256	34639.5420
1A4	2622.7420	5343.6644	3521.0773	116762.4548	175.8438	80.0077	10732.8645	35166.5589	14353.1223
1A5	3496.4551	2950.1868	5367.6455	99081.5744	309.7676	198.6740	14424.6476	63722.6329	13055.4049
1A6	1610.2831	8464.9818	7445.7405	165328.3082	205.2787	7104.6219	9098.7007	14231.9647	39368.5609
1A7	1550.9756	11282.4873	7508.0925	155648.2734	30.0521	8624.9735	5233.5670	13974.6238	16725.3534
1A8	4018.5772	11684.3387	14110.4938	249916.6552	106.1071	131.1989	6446.5981	1843.7671	24261.0449
1B1	1410.5735	16541.9715	14044.2810	111054.2628	1026.8353	5826.3882	13103.1900	2170.4342	5901.5905
1B2	2783.6918	22258.4313	12215.2198	211972.4868	3867.2100	12855.7751	36299.2465	2074.3727	7821.7662
1B3	4731.9098	73298.5746	11694.9547	266562.4536	1611.5377	12029.4467	23580.0797	320.6634	10593.5361
1B4	7174.5450	53382.6281	14474.2806	247981.8784	206.3577	6259.3180	11913.8462	4204.0387	13875.4342
1B5	3479.1665	71776.4667	13987.9299	443158.1467	1731.4817	16664.7840	9167.0712	1456.6277	14902.5509
1B6	4877.2420	40281.4843	30303.9019	263465.7448	1090.7681	9293.1998	18956.4632	3828.1307	9516.6262
1B7	3417.7468	35017.3627	19770.3973	205713.3007	3459.3904	10539.4461	9675.6646	4589.8021	18367.3594
1B8	2637.6244	39223.2787	14116.4339	337151.6740	1579.2802	11152.0223	13997.8256	11157.9887	14001.9146
1C1	1215.5899	8281.3329	6654.4910	197686.9709	817.0329	167.3714	1994.1541	2419.6263	33280.9653
1C2	1726.8693	10383.5851	12050.6122	182779.1755	6.5168	162.7644	2965.0502	14723.7275	37003.1469
1C3	799.4650	8297.3369	5952.3747	244033.1936	32.3636	274.7389	2638.7482	2420.5824	42179.1752
1C4	2061.7570	14004.5611	10223.1465	231725.3765	504.1792	140.3625	2625.5804	5660.1154	34306.8788
1C5	1258.2621	10133.6384	7736.8555	171923.9741	630.8622	295.5897	3101.4631	2413.8761	31549.2803
1C6	839.6099	3714.2786	8146.6553	191681.6491	291.8906	384.5440	1889.8858	7185.1824	32588.7097
1C7	1059.4218	7373.2661	11861.3370	161951.2713	1195.3775	74.0176	2476.7841	1426.3461	30015.8184
1C8	974.8838	6800.8161	8887.8320	228579.9305	38.8362	210.7815	9501.9212	10662.8299	29896.8887
QC01	2807.1458	20842.9824	8554.5983	216385.7511	647.7586	4833.7707	6736.2958	5741.0323	19810.2281
QC02	2828.9335	20824.4481	9215.2966	216480.7015	661.2771	4904.0256	6726.6131	5683.8166	19813.2757
QC03	2806.4521	20314.2910	9247.4599	218238.8834	694.9376	4712.3165	6864.2118	5734.6171	19896.1802
kegg_map	ko00061,ko01100	ko00061,ko00333,ko01100,ko01110	ko00592,ko01040,ko01100,ko01110	ko00061,ko00073,ko01040	-	-	ko00100,ko01100,ko01110	-	ko01100

Note: 1A1-1A8: Samples in Pre-PAMK group; 1B1-1B8: Sample in Model group; 1C1-1C8: Sample in Control group;

Fig. 7b Representitive differentially expressed genes (DEGs) in mouse spleen.

Gene name	Control 1	Control 2	Control 3	Model 1	Model 2	Model 3	Pre-PAMK250 1	Pre-PAMK 250 2	Pre-PAMK 250 3	Control vs Model		Model vs Pre-PAM250	
										log_2Fold Change	p value	log_2Fold Change	p value
mt-Atp6	42.0632	41.1435	26.5376	36.0769	36.2599	33.628	92.4766	58.5152	62.0223	-0.06826	0.63729	1.03234	0.00000
Serpina1a	4.0778	3.9759	5.9075	3.0438	1.9429	3.0823	8.9123	7.0018	5.5404	-0.80852	0.00035	1.43369	0.00000
Ccl19	66.8204	55.8964	50.827	34.0047	41.8233	34.7961	81.0444	53.1774	72.3194	-0.666	0.00000	0.9242	0.00000
Hp	13.2771	14.0296	14.1548	20.6263	24.6907	23.4076	12.0182	15.2541	12.81	0.71119	0.00000	-0.75571	0.00000
Fam107a	5.8038	5.4206	3.5726	0.7989	1.5377	0.8782	2.2752	2.4316	2.7073	-2.21997	0.00000	1.22864	0.00000
Bnc1	0.8668	0.2179	0.1881	1.51	1.8657	1.1954	0.324	0.4493	0.8046	1.84147	0.00785	-1.51016	0.00000
Tnxb	16.8542	12.7375	13.821	13.9539	16.221	14.0596	7.0425	9.8454	10.422	0.01156	0.89724	-0.67479	0.00000
Rgs13	3.9459	3.9023	2.5031	2.2351	2.0498	2.1167	5.1793	3.653	5.1438	-0.71185	0.00213	1.14865	0.00000
Slit3	3.5961	1.6728	1.818	3.4034	4.3175	2.9215	1.038	2.2356	1.5201	0.57631	0.12277	-1.12705	0.00000
Adamtsl1	0.6355	0.2282	0.2109	0.4887	0.9453	0.5488	0.169	0.2553	0.2992	0.87894	0.12890	-1.43162	0.00000
Trf	73.8375	67.0616	82.7898	135.0645	120.6774	121.3884	53.9207	84.7154	86.9504	0.73451	0.00000	-0.72007	0.00000
Foxp3	4.9722	3.6284	3.8806	2.3884	3.5724	2.833	6.9816	4.5599	5.0552	-0.51997	0.00123	0.93925	0.00000
Gdf6	1.2747	1.4436	1.9792	5.6116	5.2277	5.7436	3.1203	3.5276	3.5381	1.8025	0.00000	-0.68036	0.00000
Pcx	3.8617	2.8913	4.3797	5.5523	6.5994	6.2592	3.4351	4.3579	3.5132	0.71011	0.00000	-0.68107	0.00000
Icos	7.8753	6.5831	6.4162	5.0703	5.756	4.573	7.9052	7.2649	8.9889	-0.45679	0.00020	0.67299	0.00001
H1f2	19.3771	16.6845	23.269	51.2814	44.8483	40.5876	24.9693	25.8474	34.6659	1.18512	0.00000	-0.65402	0.00001
Slc6a4	5.2301	6.1623	5.92	5.0689	4.4187	6.1274	3.0758	2.8078	3.3241	-0.16674	0.20416	-0.74123	0.00001
Galnt15	2.2445	0.8137	0.9741	2.8277	5.5914	2.6365	1.6223	1.7369	1.7253	1.44843	0.00926	-1.09807	0.00001
Glis3	0.8337	0.9995	1.1127	1.8357	1.6668	1.6853	0.6463	1.1356	0.9566	0.79676	0.00000	-0.89728	0.00001
Cmtm4	5.4373	4.8848	6.287	6.4247	5.8534	6.5102	3.1968	4.3843	4.5016	0.16025	0.10687	-0.61482	0.00002
Tox	1.9311	1.6337	1.5721	1.2335	1.5203	1.3124	2.2837	1.9691	2.3021	-0.35415	0.01736	0.71114	0.00002
Tenm4	0.7319	0.6561	0.8129	1.7755	1.7885	1.751	0.6658	1.328	0.9969	1.25489	0.00000	-0.80768	0.00003
Ntn1	1.797	1.083	1.3447	2.41	2.8972	2.3156	1.0683	1.4908	1.7126	0.83669	0.00000	-0.81231	0.00003
Osr1	3.1609	0.4701	0.2757	2.218	4.7378	2.0499	0.9879	1.0178	1.3207	1.21105	0.24489	-1.41393	0.00004
Wnt2b	3.8627	0.6935	0.7026	1.8662	3.4155	1.5557	0.5691	0.7133	1.3379	0.38086	0.65581	-1.36155	0.00005
Cxcl9	16.8793	17.0666	15.1605	7.4504	10.0313	8.8239	14.6746	10.9465	15.5966	-0.91783	0.00000	0.66956	0.00005
Hspb1	21.5456	14.6479	21.597	12.6913	20.9297	17.7494	9.3887	7.0877	11.0093	-0.1838	0.30537	-0.88241	0.00006
Fcgr1	2.6179	2.7946	3.5002	4.926	4.7259	5.3516	2.2523	3.3504	3.0563	0.73409	0.00001	-0.76986	0.00006
Ptgis	5.5698	1.4566	1.6138	3.8714	4.8097	3.9921	1.7491	1.9865	2.9336	0.5505	0.37072	-0.90386	0.00006
Fbln1	5.2328	2.5013	1.6123	2.9703	4.2399	3.1601	1.642	1.6128	2.4112	0.14356	0.77345	-0.85041	0.00007
Mmp9	2.1389	3.1422	2.6484	5.2729	4.1487	5.2447	2.2738	3.5949	2.4209	0.8662	0.00000	-0.79949	0.00006
Krt19	8.1611	1.3089	1.9541	4.762	7.945	4.1433	1.6346	1.7288	3.589	0.56192	0.48762	-1.25538	0.00007
Tox2	2.5748	3.1983	3.1517	2.0129	2.1749	2.5673	5.3665	3.5707	3.3053	-0.41949	0.01612	0.8795	0.00007
Spon2	0.8394	0.8733	1.0367	5.4517	4.6424	5.0386	2.8493	3.0504	3.4997	2.44333	0.00000	-0.66381	0.00008
Hcar2	2.7685	2.2393	3.3001	6.9799	9.6822	9.3733	4.4111	5.4205	5.5701	1.63374	0.00000	-0.73643	0.00008
Adamts2	1.2612	1.2275	0.9152	1.8332	2.3292	1.4729	1.0618	1.0586	1.1866	0.70772	0.00005	-0.74498	0.00008
Zfhx4	0.1289	0.0401	0.0165	0.0947	0.2297	0.103	0.0333	0.0301	0.0416	1.20285	0.16263	-2.00289	0.00010
F13a1	3.4788	3.1119	2.3583	3.1204	4.0426	3.692	1.7993	2.7529	1.9752	0.26276	0.06584	-0.71177	0.00010
Cldn15	4.7284	1.6232	1.6578	4.029	5.8652	3.8893	1.4001	2.0048	3.0574	0.7776	0.14327	-1.0703	0.00011
Nbl1	17.6673	5.016	6.4696	11.7317	16.7966	11.6502	6.2815	6.3096	10.0926	0.45891	0.41699	-0.80332	0.00011
Ces1d	3.0281	0.3485	2.5793	6.2225	7.2071	5.8707	4.5783	2.9736	3.7317	1.68742	0.03355	-0.7522	0.00011
Gm14453	8.9464	8.6933	12.6413	12.2799	13.3757	14.4657	6.3268	7.32	7.9931	0.38983	0.05322	-0.86863	0.00011
Klra2	1.0426	1.2792	0.9822	1.978	2.1097	1.6528	0.6452	0.8586	0.8625	0.77465	0.00355	-1.25213	0.00011
Cxadr	1.7851	0.9318	1.4205	1.8652	2.5327	2.0561	1.1741	1.3143	1.4454	0.62867	0.00127	-0.69203	0.00012
Per2	7.4768	6.4265	7.0388	1.763	2.0229	1.75	2.9474	2.9086	2.516	-1.9373	0.00000	0.62001	0.00014
Epha4	0.1966	0.1748	0.1607	0.4661	0.4488	0.4025	0.1158	0.2023	0.21	1.28835	0.00001	-1.29094	0.00015
Clec3b	32.6165	7.5384	9.0828	18.1435	26.6861	15.6537	9.5814	7.6313	14.4677	0.29519	0.66504	-0.91071	0.00018
Lamb1	1.4717	0.9548	0.7519	1.4864	2.131	1.2706	0.7585	1.0231	0.9795	0.60664	0.00546	-0.80021	0.00018
Trmo	6.1215	10.3491	9.6679	6.9529	9.061	12.133	14.4816	14.7227	15.3065	0.08695	0.79981	0.67983	0.00020
Col6a6	1.1011	0.1907	0.1977	0.4096	1.3106	0.5305	0.2736	0.2352	0.3215	0.59993	0.51980	-1.41814	0.00020
Rims3	3.5684	4.6354	5.7616	8.5045	9.111	10.2707	4.1181	7.2143	6.2634	0.97686	0.00002	-0.6444	0.00020
Cd14	27.1361	29.4384	34.9503	32.4501	39.5918	31.9383	13.2986	18.6063	28.2932	0.16427	0.13587	-0.76825	0.00020
Vcl	6.9762	6.7583	7.7759	8.5602	9.0643	10.3407	3.3388	7.413	5.4497	0.36165	0.00008	-0.7682	0.00022
Abca6	0.6599	0.3598	0.2494	0.5318	0.7521	0.5618	0.1944	0.3214	0.2022	0.52682	0.10572	-1.33554	0.00022
Flrt2	2.9731	1.3773	1.509	2.4098	3.5799	2.58	1.0271	1.9541	1.7599	0.53954	0.15826	-0.83256	0.00022
Muc16	0.9482	0.153	0.2299	0.3834	0.8041	0.5219	0.2102	0.2215	0.3956	0.36451	0.65430	-1.02778	0.00029
Il17rb	2.6692	1.7946	1.6902	0.8489	1.3802	1.2694	1.9977	2.3541	1.9431	-0.82693	0.00042	0.86813	0.00032
Grem2	3.329	0.7391	0.9402	2.0049	3.876	2.0663	0.9654	1.0388	1.734	0.66543	0.35432	-1.06664	0.00035
Mllt11	7.6544	7.7776	6.7307	5.3937	5.1534	5.4806	9.8531	7.4724	6.9328	-0.48569	0.00012	0.62091	0.00035
Gm47547	1.7142	1.6825	2.1186	3.4799	3.4029	2.9871	0.9672	2.4609	1.3275	0.82101	0.00001	-1.02749	0.00035
Plxdc2	3.0623	1.8523	2.1318	2.6226	3.4504	2.8825	1.6116	1.9827	2.1837	0.33258	0.04379	-0.61014	0.00036
mt-Co3	53.7013	44.2856	34.7419	38.3793	39.2283	42.8147	91.1863	64.3727	43.0852	-0.15525	0.22266	0.74692	0.00037
Nkain4	6.868	1.215	1.4628	3.0301	7.7205	3.1227	0.6589	1.3153	2.5986	0.54165	0.53414	-1.58006	0.00039
